# Analysis of Nidogen-1/Laminin γ1 Interaction by Cross-Linking, Mass Spectrometry, and Computational Modeling Reveals Multiple Binding Modes

**DOI:** 10.1371/journal.pone.0112886

**Published:** 2014-11-11

**Authors:** Philip Lössl, Knut Kölbel, Dirk Tänzler, David Nannemann, Christian H. Ihling, Manuel V. Keller, Marian Schneider, Frank Zaucke, Jens Meiler, Andrea Sinz

**Affiliations:** 1 Department of Pharmaceutical Chemistry and Bioanalytics, Institute of Pharmacy, Martin Luther University Halle-Wittenberg, Halle (Saale), Germany; 2 Department of Chemistry and Center for Structural Biology, Vanderbilt University, Nashville, TN, United States of America; 3 Center for Biochemistry, Medical Faculty, University of Cologne, Cologne, Germany; 4 Research Group Artificial Binding Proteins, Institute of Biochemistry and Biotechnology, Martin Luther University Halle-Wittenberg, Halle (Saale), Germany; University of Queensland, Australia

## Abstract

We describe the detailed structural investigation of nidogen-1/laminin γ1 complexes using full-length nidogen-1 and a number of laminin γ1 variants. The interactions of nidogen-1 with laminin variants γ1 LEb2–4, γ1 LEb2–4 N836D, γ1 short arm, and γ1 short arm N836D were investigated by applying a combination of (photo-)chemical cross-linking, high-resolution mass spectrometry, and computational modeling. In addition, surface plasmon resonance and ELISA studies were used to determine kinetic constants of the nidogen-1/laminin γ1 interaction. Two complementary cross-linking strategies were pursued to analyze solution structures of laminin γ1 variants and nidogen-1. The majority of distance information was obtained with the homobifunctional amine-reactive cross-linker *bis*(sulfosuccinimidyl)glutarate. In a second approach, UV-induced cross-linking was performed after incorporation of the diazirine-containing unnatural amino acids photo-leucine and photo-methionine into laminin γ1 LEb2–4, laminin γ1 short arm, and nidogen-1. Our results indicate that Asn-836 within laminin γ1 LEb3 domain is not essential for complex formation. Cross-links between laminin γ1 short arm and nidogen-1 were found in all protein regions, evidencing several additional contact regions apart from the known interaction site. Computational modeling based on the cross-linking constraints indicates the existence of a conformational ensemble of both the individual proteins and the nidogen-1/laminin γ1 complex. This finding implies different modes of interaction resulting in several distinct protein-protein interfaces.

## Introduction

**Figure 1:**
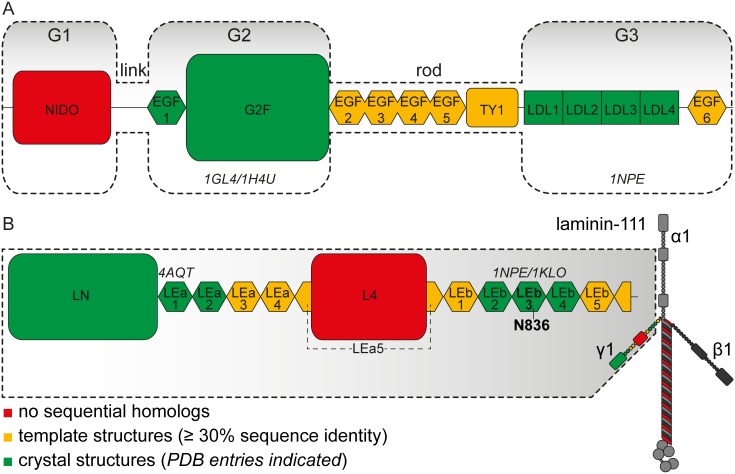
Arrangement of (A) nidogen-1 and (B) laminin γ1 short arm. The domains are color-coded with respect to the availability of crystal structures (green), template structures for comparative modeling (yellow) or neither of both (red). PDB IDs of the respective crystal structures are indicated in italics. (A) Nidogen-1 domain assignments are according to the UniProt KB entry P10493. Additionally, the historic domain names (G1–link–G2–rod–G3) are given. (B) Laminin domain designations follow the nomenclature of Aumailley *et al.* [5]. Laminin γ1 short arm is part of the heterotrimeric protein laminin-111, the overall structure of which is schematically depicted.

Laminins are the major non-collagenous proteins of basement membranes that are known to form networks through crucial non-covalent self-interactions [1], [2]. Each member of the laminin protein family consists of three polypeptide chains with one copy of the α, β, and γ chain. Since the discovery of laminin [3], several nomenclatures have been developed, which are, however, not always completely systematic [4]. In this work, we apply the laminin nomenclature introduced by Aumailley *et al.* [5]. Electron microscopic studies of laminin-111 (α1-, ß1-, and γ1-subunits) reveal a cross-shaped protein structure [6] with three subunits being connected within the central part according to a coiled-coil arrangement (‘long arm’). The *N*-terminal regions of the laminin subunits are free (‘short arms’) (Figure 1). The globular domains at the *N*-termini of all three chains (LN domains) are required for efficient polymerization as deletion mutants with two or fewer LN domains fail to form networks [2]. Furthermore, laminin-111 harbors more centrally located globular domains (α1 L4a, α1 L4b, β1 LF, and γ1 L4) as well as several ‘laminin-type epidermal growth factor-like’ (LE) modules. Three-dimensional structures of certain laminin domains are available, such as X-ray structures of the *C*-terminal LG domains (PDB entries 2JD4, 2WJS, 1QU0, 1DYK), the nidogen-binding region γ1 LEb2–4 (PDB entries 1KLO, 1NPE), and the α5, ß1, and γ1 LN domains (PDB entries 2Y38, 4AQT, 4AQS), the latter of which were found to be in good agreement with previously reported computational models [7].

Nidogens (entactins) are sulfated monomeric glycoproteins that are ubiquitously present in basement membranes of higher organisms. While invertebrates possess only one nidogen, two nidogen isoforms, namely nidogen-1 (ca. 135 kDa) and nidogen-2 (ca. 150 kDa), have been identified in vertebrates [8]–[10]. Both isoforms exhibit a similar repertoire of binding partners [11], resulting in basement formation even after knocking out one nidogen isoform [12]. Electron microscopic studies have revealed nearly identical arrangements of nidogen-1 and -2 [9], [13]. Both comprise three globular domains (G1–G3), which are connected by two rod-shaped domains (‘link’ and ‘rod’ regions) (Figure 1). Sequence analyses have confirmed the modular structures of nidogens. Identified motifs, such as epidermal growth factor (EGF)-like sequences, are also present in a number of other proteins of the extracellular matrix [14]. X-ray structures are available for G2 and G3 domains of nidogen-1 (PDB entries 1GL4, 1H4U, 1NPE). Nidogen is considered to be a stabilizer and adaptor protein within the basement membrane. In addition to isoform-specific interactions of nidogen-1 with fibulin-1 and -2 as well as of nidogen-2 with tropoelastin and type XVIII collagen, both nidogens bind to the essential basement membrane proteins perlecan, type IV collagen, and laminin [14].

Initial studies of nidogen-1 revealed an extraordinarily strong interaction with laminin [15] and led to the elucidation of a 1∶1 stoichiometry between nidogen-1 and laminin in the complex [16]. The *C*-terminal G3 domain of nidogen-1 [16], [17] as well as the EGF-like motif LEb3 in laminin γ1 [18]–[21] were identified as binding regions. A weaker nidogen-1 binding has been observed in laminin γ2 and γ3 [14]. Using radioligand binding assays with variants of this domain, amino acids Asp-834, Asn-836, and Val-838 were identified to be essential for the interaction of the laminin γ1 LEb3 domain with nidogen-1. The exchange of Asn-836 against aspartic acid resulted in a drastic decrease of nidogen-1 binding with a 25,000-fold loss in affinity [22]. The sequence numbering used here follows the amino acid sequences shown in Figure S1 with Asp-834, Asn-836, and Val-838 corresponding to Asp-800, Asn-802, and Val-804 in the classical laminin γ1 numbering [23].

In 2003, the three-dimensional structure of the complex between the G3 domain of nidogen-1 and LEb domains 2–4 of laminin γ1 was elucidated [24]. The G3 domain exhibits a ß-propeller composed of six LDL receptor YWTD modules, creating a concave interface for the amino acids Asp-834, Asn-836, and Val-838 within the loop of laminin γ1 LEb3. Complex formation is enhanced by additional interactions of laminin γ1 LEb2 with the ß-propeller.

Recently, the interaction of full-length nidogen-1 with laminin γ1 short arm has been investigated by size-exclusion chromatography, dynamic light scattering, and small-angle X-ray scattering [25]. These studies indicate that the interaction is mediated solely by the *C*-terminal domains, while the remaining regions of both proteins do not participate in complex formation.

For our studies investigating the interaction between nidogen-1 and laminin γ1 short arm, we chose an alternative approach providing 3D-structural insights into proteins. This strategy relies on chemical cross-linking and a subsequent mass spectrometry (MS)-based analysis of the created products [26], [27]. Structural information can be obtained by the insertion of a chemical cross-linker between two functional groups within a protein. The cross-linker has a defined length and is connected via covalent bonds to functional groups of amino acid side chains, allowing the cross-linked amino acids to be identified after enzymatic digestion. This chemical cross-linking approach is also applied to study protein-protein interfaces. The sequence separation of cross-linked amino acids, combined with the cross-linker length, impose a distance constraint on the 3D structure of the protein complex [7], [28]–[31]. Analysis of cross-linked peptides by MS makes use of several advantages: First, the mass of the protein or the protein complex under investigation is theoretically unlimited as the proteolytic peptides of the cross-linked proteins are analyzed – in case a “bottom-up” strategy is employed for MS protein analysis. Second, the experiment is rapid and requires very low (10^−14^–10^−15^ mol) amounts of protein. Finally, as the cross-linking reaction can be executed in a native-like environment, protein structure and flexibility are accurately reflected. It is possible to study membrane proteins, post-translational modifications as well as splice variants. The broad range of cross-linking reagents with different specificities (primary amines, sulfhydryls, or carboxylic acids) and the wide range of distances (0 Å up to 20 Å) allow a setup of fine-tuned experimental strategies.

However, despite the straightforwardness of the cross-linking approach, the identification of the cross-linked products can be cumbersome due to the complexity of the reaction mixtures. Several strategies have been employed to enrich cross-linker-containing species by affinity chromatography or to facilitate the identification of the cross-linked products, e.g. by using MS/MS cleavable cross-linkers or isotope-labeled cross-linkers or proteins [26], [27], [32].

By combining sparse distance constraints from disulfide bonds and cross-links imposed by *bis*(sulfosuccinimidyl)glutarate (BS^2^G), MS identification of the cross-linked products, and computational modeling we predicted a galactose-binding domain-like fold for laminin ß1 and γ1 LN domains [7]. This fold was later confirmed by crystal structures of the α5 LN-LE1–2 [33], the β1 LN-LEa1–4, and the γ1 LN-LEa1–2 fragments [34].

In this work, we extend the cross-linking tool box from the exclusive use of amine-reactive cross-linkers towards the incorporation of photo-reactive amino acids that can deliver valuable short-distance information [35]. Combined with a mass spectrometric analysis of the created cross-links and computational modeling, we were able to gain detailed insights into the interaction mechanisms between full-length nidogen-1 and the laminin variants γ1 LEb2–4, γ1 LEb2–4 N836D, γ1 short arm, and γ1 short arm N836D. Our results suggest the existence of multiple nidogen-1/laminin γ1 interfaces in addition to the known interaction site.

## Materials and Methods

### Materials

The cross-linking reagent BS^2^G and the photo-amino acids (photo-methionine and photo-leucine) were obtained from Thermo Fisher Scientific. The proteases trypsin (porcine), chymotrypsin, and GluC were obtained from Promega, all other chemicals were purchased from Sigma. Solvents used for nano-high performance liquid chromatography (HPLC) were spectroscopic grade (Uvasol, VWR). Milli-Q water was produced by a TKA Pacific system with X-CAD dispenser from Thermo Electron LED GmbH (part of Thermo Fisher Scientific).

### Expression and Purification of Nidogen-1 and Laminin γ1 Variants

Genes encoding all proteins (murine amino acid sequences, see Figure S1) were expressed in human embryonic kidney (HEK) 293 EBNA cells with *N*-terminal (His)_6_-tag (nidogen-1) or with *N*- or *C*-terminal double Strep tag II (laminin γ1 variants γ1 LEb2–4, γ1 LEb2–4 N836D, γ1 short arm, and γ1 short arm N836D). Incorporation of photo-reactive amino acids was achieved by growing the cells in a Leu- and Met-depleted medium (DMEM-LM, Thermo Fisher Scientific) to which photo-Met and photo-Leu were added. Strep II-tagged proteins were purified using Strept-Actin sepharose matrix (IBA), (His)_6_-tagged nidogen-1 was purified with Nickel-NTA Superflow matrix (GE Healthcare) using an ÄKTA FPLC system (GE Healthcare). Amino acid sequences were confirmed by peptide fragment fingerprint analysis.

### Surface Plasmon Resonance Spectroscopy (SPR)

Experiments were conducted at 25°C using a Biacore T100 instrument (GE Healthcare). Nidogen-1 was immobilized by covalent coupling on a Series S Sensor Chip CM 5 (GE Healthcare) using amine-coupling chemistry. A 1∶1 mixture of 100 mM *N*-hydroxysulfosuccinimide and 400 mM 1-ethyl-3-(3-dimethylaminopropyl)carbodiimide hydrochloride (Pierce) was passaged over the sensor chip surface for 7 min at a flow rate of 10 µl/min to activate the matrix. For protein immobilization, a solution of 300 nM nidogen-1 in 10 mM sodium acetate (pH 5.5) was injected (contact time 35 min, flow rate 10 µl/min). Remaining activated groups were blocked by injecting 1 M ethanolamine (pH 8.5) for 7 min at a flow rate of 10 µl/min. As reference, a flow cell was prepared in the same manner omitting the injection of protein solution.

Binding assays using all laminin variants (wild type and N836D) as mobile analytes were performed as single-cycle kinetic experiments [36] at a constant flow rate of 30 µl/min. The analyte was diluted in running buffer (20 mM HEPES, 100 mM NaCl, 0.05% (*v/v*) Tween 20, pH 7.5) and 1 min injections of these dilutions were applied with increasing concentrations. Individual analyte injections were followed by a 40.6 min flow of running buffer to allow for partial dissociation. Blank cycles, where running buffer was injected instead of analyte solution, were performed prior to each analyte cycle to facilitate double referencing. After completion of each analysis cycle, the sensor chip surface was extensively washed with running buffer to achieve complete dissociation of the analyte. Response data were doubly referenced and kinetic parameters were determined as described previously [36] using BIAevaluation software 4.1.1 (GE Healthcare) to fit a 1∶1 binding model to the data.

### Enyzme-Linked Immunosorbent Assays (ELISA)

ELISA-based binding assays were performed in 96-well plates (Nunc Maxisorp, Thermo Fisher Scientific). Laminin fragments were coated overnight at 4°C with a concentration of 10 µg/ml in 50 µl Tris-buffered saline (TBS) per well (50 mM Tris-HCl, 150 mM NaCl, pH 7.4). The supernatant was discarded after coating and the plates were washed once with a TBS-T solution (TBS with 0,05% (*v/v*) Tween-20) with a volume of 400 µl/well. Unspecific binding sites were blocked with 50 µl/well of 1% (*w/v*) bovine serum albumin (BSA) in TBS-T solution for 1 h at room temperature. Increasing concentrations of nidogen-1 ranging from 0.03 nM to 234 nM in TBS-T/1% BSA were added to the wells until saturation was reached. After ligand incubation for 1 h at room temperature plates were washed three times with TBS-T solution. Nidogen-1 was detected with mouse monoclonal antibodies directed against the (His)_6_-tag (Qiagen, dilution 1∶2000). Antibodies were added in 50 µl TBS-T/1% BSA per well and incubated for 1 h at room temperature. Plates were then washed three times with TBS-T solution and the secondary horseradish peroxidase-conjugated α-mouse IgG antibodies raised in rabbit (Dako, 1∶2000 diluted in TBS-T/1% BSA) were added. Plates were washed three times with TBS-T solution and one time with water. For signal detection, 50 µl/well tetramethylbenzidine solution (1-Step ultra TMB-ELISA solution, Thermo Fisher Scientific) was used. The color reaction was stopped with 50 µl/well of 10% H_2_SO_4_ solution and absorption at 450 nm was determined in an ELISA reader (Tecan), subtracting the background (0 nM ligand). As a second control, increasing ligand concentrations were added to uncoated wells. All measurements were performed in triplicates. K_d_ values were calculated using Origin v6.0.

### Cross-Linking Reactions

Cross-linking reactions were conducted with 1 to 10 µM protein solutions in 20 mM HEPES, 100 mM NaCl, pH 7.5. Freshly prepared stock solutions of the homobifunctional amine-reactive *N*-hydroxysuccinimide ester BS^2^G (in dimethylsulfoxide) were added in 200-fold molar excess to the protein solution. The reactions were conducted at room temperature and were quenched after 30 min by adding NH_4_HCO_3_ to a final concentration of 20 mM.

For photo-cross-linking, nidogen-1 (0.5 µM) was incubated with laminin γ1 short arm (1 µM) or γ1 LEb2–4 (10 µM) for 20 min at room temperature. Then, the samples were irradiated with UV-A light (max. 360 nm) at 8000 mJ/cm^2^ in a home-built device [37].

### In Gel Digestion

After SDS-PAGE of the cross-linking reaction mixtures, bands of interest were excised, reduced with dithiothreitol, alkylated with iodoacetamide, and digested. For *in situ* digestion, either GluC or Chymotrypsin was added and gel pieces were incubated at 4°C for 1 h before trypsin was added (enzyme:substrate ratio 1∶100). The digestion was performed overnight at 37°C. Peptides were extracted and samples were concentrated in a vacuum concentrator to volumes between 60 to 120 µl before LC/MS/MS analysis.

### In Solution Digestion

For *in solution* digestion, proteins were incubated with acetone (–20°C, 1 h) to precipitate them from solution. The pellet was dried, solubilized with 1.6 M urea, reduced, alkylated, and digested with a mixture of GluC and trypsin (enzyme:substrate ratio 1∶50).

### Nano-HPLC/Nano-ESI-LTQ-Orbitrap-MS/MS

Fractionation of peptide mixtures was carried out on an Ultimate nano-HPLC system (Thermo Fisher Scientific) using reversed phase C18 columns (precolumn: Acclaim PepMap, 300 µm **•** 5 mm, 5 µm, 100 Å, separation column: Acclaim PepMap, 75 µm **•** 250 mm, 3 µm, 100 Å, Thermo Fisher Scientific). After washing the peptides on the precolumn for 15 min with water containing 0.1% trifluoroacetic acid, peptides were eluted and separated using gradients from 0 to 40% B (gradient times varying between 30 to 90 min), 40 to 100% B (1 min), and 100% B (gradient times varying between 11 to 30 min), with solvent A: 5% ACN containing 0.1% formic acid, and solvent B: 80% ACN containing 0.08% formic acid. The nano-HPLC system was directly coupled to the nano-ESI source (Proxeon) of an LTQ-Orbitrap XL hybrid mass spectrometer (Thermo Fisher Scientific). Data were acquired in data-dependent MS/MS mode: Each high-resolution full scan (*m/z* 350 to 2000, R = 60,000) in the orbitrap was followed by five product ion scans in the LTQ (collision-induced dissociation with 35% normalized collision energy) of the five most intense signals in the full-scan mass spectrum (isolation window 1.5 u). Dynamic exclusion (exclusion duration 120 sec, exclusion window −1 to 2 u) was enabled to allow detection of less abundant ions. Data acquisition was controlled via XCalibur 2.0.7 (Thermo Fisher Scientific) in combination with DCMS link 2.0.

### Identification of Cross-Linked Products

Cross-linked products were analyzed with StavroX v2.0.6 [38]. MS and MS/MS data were automatically analyzed and annotated. All cross-links were manually validated. A maximum mass deviation of 3 ppm between calculated and experimental precursor masses was applied as well as a signal-to-noise ratio of ≥2. Primary amino groups (Lys side chains and *N*-termini) were considered as cross-linking sites for BS^2^G; all amino acid residues were regarded as potential sites for UV-A-induced cross-linking of photo-Met and photo-Leu. Oxidation of Met was set as variable modification for all cross-linked proteins. Additionally, carbamidomethylation was included as fixed modification for Cys. Two missed cleavage sites were considered for each amino acid (Lys and Arg for trypsin; Tyr, Trp, and Phe for chymptrypsin; Glu for GluC).

### Identification of Templates for Computational Modeling

The sequences of nidogen-1 and laminin γ_1_ short arm were split into separate domains as defined by the UniProt Knowledgebase (www.uniprot.org, nidogen-1: entry P10493, laminin γ1: entry P02468) and modeled independently. The domains were subdivided in three classes according to the availability of structural models or templates (Figure 1). The first class comprises the nidogen-1 LDL-receptor class B repeats (G3 domain), EGF-like domain 1 and G2 ß-barrel domain as well as the laminin γ1 LN, LEa1, LEa2, and LEb2–4 domains, all of which have been characterized by X-ray crystallography. The remaining EGF-like domains of nidogen-1 and laminin γ1 short arm as well as the nidogen-1 thyroglobulin type-1 (TY1) domain were assigned to the second class because a DELTA-BLAST [39] search run had led to the identification of homologous domains with existing crystal structures that could serve as templates for comparative modeling. Finally, the third class comprises the nidogen-1 G1 domain and the laminin γ1 L4 domain, for which we did not identify any sequential homologues. Both domains were searched against the threading servers PHYRE2 [40], HHPred [41], PSIPRED (pDomTHREADER and GenTHREADER) [42], and I-TASSER [43] for fold recognition.

All following modeling experiments were performed with Rosetta v3.4. The Rosetta total scores reported herein were calculated using the score12 full-atom scoring function. Full command lines for each step are included in Files S1 and S2.

### Comparative Modeling Based on Sequential Homologous Templates

After comparing the DELTA-BLAST sequences of potential template structures with the actual sequences of the corresponding PDB entries, sequence alignments of all target and template sequences were performed with ClustalW 2.1 [44]. Only templates showing ≥30% sequence identity were used for comparative modeling. The corresponding alignments are shown in Figure S2.

The LE domain crystal structures (PDB entries 4AQS, 4AQT, 1NPE, 1KLO, 2Y38) were considered as templates for the remaining LE domains, namely LEa3–5, LEb1 and LEb5. LEa5 is split in two parts by the L4 domain (Figure 1). The N-terminal LEa5.1 was not modeled as an individual domain since it comprises only ten residues. Similarly, LEb6 was not modeled as an individual domain since only nine residues are contained in the laminin γ1 short arm fragment used here.

We identified EGF-like domains in 14 X-ray structures (PDB entries 1GL4, 1YO8, 1SZB, 1TOZ, 1UZJ, 2BO2, 3P5B, 1NFU, 3H5C, 3POY, 3QCW, 3S94, 3V64 and 2W86) as templates for nidogen-1 EGF2–6. The nidogen-1 TY1 domain was modeled using TY1 domains within the crystal structures 1ICF and 2DSR as templates. Threading of the primary sequences onto the 3D template structures, modeling of missing loop regions, and clustering of the created models were carried out as reported previously [45]. For each template/target sequence pair, 1000 models were constructed. All structural models for one target sequence were ranked according to their Rosetta total score and the best-scoring 10% were used for clustering with automated detection of the clustering radius by Rosetta. An overview of all clusters is given in Table S1. The best-scoring structures within the top three clusters were considered as final models.

### Comparative Modeling of Laminin γ1 L4

Using the threading servers listed above, 21 potential structural homologues were identified for the L4 domain, 13 of which exhibit a ß-sandwich topology and carbohydrate-binding activity. Structural alignment with MUSTANG [46] revealed that these templates share a common topology that is in accordance with the PSIPRED [47] and JUFO9D [48] secondary structure prediction for L4. Hence, we hypothesize that the laminin γ1 L4 domain shares the galactose-binding domain-like fold and built the comparative model based on these templates. ClustalW 2.1 alignments of the laminin γ1 L4 domain with all 13 templates identified by fold recognition (PDB entries 1GU3, 1GUI, 1K42, 1CX1, 1WKY, 1WMX, 3OEA, 2ZEW, 2ZEZ, 1DYO, 3F95, 3ZXJ, 1D7B) revealed sequence identities of 3–14%. Hence, the alignments had to be adjusted manually to guarantee for correctly aligned secondary structure elements (Figure S3). Adjustments were based on PSIPRED secondary structure prediction for L4, DSSP secondary structure annotations of the template structures [49] and manual inspection of all templates in Pymol v1.5 (Schrödinger LLC). After optimization of the alignments, comparative modeling was performed as described above. For each template, 1000–2000 models were generated (25,296 models in total) and the top 10% were selected for clustering (Table S2). To obtain informative clustering results, long loop regions (>5 residues) were not considered for root-mean square deviation (RMSD) calculation. The clustering radius was set to 2 Å.

### *De Novo* Folding of the Nidogen-1 NIDO Domain

No likely structural homologues of the nidogen-1 NIDO domain were identified by fold recognition. Hence, models were generated by *de novo* folding using Rosetta AbinitioRelax within the Rosetta Topology Broker framework [50]. Initially, Rosetta fragment picker was used to create a fragment library consisting of the primary sequence split into overlapping 3-mers and 9-mers, each of them represented by 200 peptide structures, mimicking the entire distribution of conformations these segments are likely to adopt in a protein structure [45], [51]. Full atom refinement during *de novo* modeling resulted in partial unfolding of the created models. Therefore, we generated 11,902 centroid models, omitting full-atom refinement after running AbinitioRelax. To filter for structures that are likely to occur in nature we pursued two complementary strategies.

First, the 10% best-scoring models were compared to a precompiled PISCES library of structurally diverse PDB models (soluble proteins, sequence ID <25%, resolution <2.0 Å) [52] using MAMMOTH [53]. This served to evaluate whether the domain adopts a known fold. Structural homologues for two of the generated models were identified (>50% of target sequence aligned, MAMMOTH Z-score >5).

Second, we generated 10,000 to 20,000 models for sequences of six homologous NIDO domains present in other organisms. Homologous domains were identified with DELTA-BLAST. Non-redundant sequences with an identity of >75% to the murine NIDO domain were selected and manually compared using Jalview [54]. The sequences of *Sarcophilus harrisii*, *Rattus norvegicus*, *Homo sapiens*, *Bos taurus*, *Cricetulus griseus*, and *Felis catus* were chosen, each of them showing differences in diverse regions that are otherwise conserved (Figure S4). To identify common topologies among the different NIDO domains, models were ranked based on their total centroid score as well as their strand pairing energy score and MAMMOTH was used to compare the best 1% models of each homologue with the best 10% models of the murine NIDO domain. Models of murine NIDO were regarded as representatives of a common topology when significant structural similarities (MAMMOTH Z-score >5) to at least one model of each homologue were found.

As a result, 109 candidate models were identified and selected for full-atom refinement. To prevent distortion of secondary structure element arrangement, side chains and peptide backbone were relaxed sequentially, keeping one of both fixed (‘-relax:bb_move false’ or ‘-relax:chi_move false’) before a final round of refinement in thorough relax mode was performed on the complete structure. We generated 50 models per input model, which were inspected by visualization with Pymol, ranked according to their Rosetta total score, and clustered with a radius of 1.5 Å. The best-scoring output structure for each template and the best-scoring structures representing the top 20 clusters were kept for further analysis. The two candidate models, identified by comparison with the PISCES library, were processed similarly. For each of them, the five best-scoring models and the best-scoring models representing individual clusters were included in the final list of potential NIDO models, resulting in a total of 132 models. These models were combined and re-scored (Table S3).

### Incorporation of Cross-Linking Constraints into the Nidogen-1 G3/Laminin γ1 LEb2–4 Experimental Structure

We identified eleven BS^2^G- and two photo-cross-links within the experimental structure of the nidogen-1 G3/laminin γ1 LEb2–4 complex (PDB entry 1NPE), which were used as distance constraints for adapting the 3D structure. The cross-linked residues and their respective Cα–Cα distances are listed in Table 1. The maximum Cα–Cα distance for lysine-lysine cross-links was calculated to 26 Å by adding the lysine side chain length (2×6.3 Å), the distance spanned by BS^2^G (7.5 Å [55]) and a tolerance of 5.9 Å to account for structural flexibility [56]. Acceptable Cα–Cα distances for the “zero-length” photo-cross-links varied depending on the side-chain length of the cross-linked residue. Fulfillment of cross-linking restraints was evaluated with a flat harmonic scoring function that renders an energy penalty when the Euclidean distance between cross-linked residues exceeds the allowed Cα–Cα distance [56]. A Rosetta constraint file containing all cross-links was created as described elsewhere [57]. The standard deviation granted for each cross-link was 1 Å. Additionally, distances of the hydrogen bonds formed by Asp-834, Asn-836, and Val-838 in the laminin γ1 LEb3 domain were restrained as they have been reported to be essential for high-affinity interaction [23], [24]. Structural refinement was carried out by generating 800 models using the Rosetta Relax application with an atom pair constraint scoring weight of 1.0 (‘-constraints:cst_fa_weight 1.0’). Structures ranking among the top 20 in terms of total score and atom-pair constraint score (reflecting the fulfillment of distance constraints) were selected as potential models of the nidogen-1 G3/laminin γ1 LEb2–4 core complex (Table S4).

**Table 1:** Overview of cross-links within the nidogen-1 G3/laminin γ1 LEb2–4 complex.

	Cα–Cα distances (Å)		
		model	model
cross-linked lysines	1NPE	(best atom-pair constraint score)	(best total score)
K-948 × K-953	10.4	10.9	11.1
K-1128 × K-1165	13.3	12.3	16.0
K-1072 × K-1128	16.7	19.1	16.2
K-948 × K-1144	17.9	16.4	17.6
K-850 (laminin) × K-1072 (nidogen-1)	20.9	17.5	16.9
K-948 × K-1152	22.2	21.2	22.2
K-1032 × K-1072	27.1	27.1	27.0
K-961 × K-1072	28.7	28.0	28.2
K-864 (laminin) × K-1152 (nidogen-1)	32.2	22.4	27.1
K-850 (laminin) × K-953 (nidogen-1)	33.0	29.5	29.4
K-1032 × K-1152	35.8	35.4	35.4
Photo-L-990 × Arg-1038	24.7	23.4	23.5
Photo-L-844 (laminin) × K-1072 (nidogen-1)	33.8	19.4	20.8

Cα–Cα distances of cross-linked residues were determined for the unmodified crystal structure (PDB entry 1NPE) as well as for the Rosetta models with the best Rosetta total score and atom-pair constraint score, respectively (shown in Figure 6). For intermolecular contacts, residues are assigned to the respective protein. All other cross-links are located within nidogen-1.

## Results

As there are only very limited structural data available for full-length nidogen-1/laminin γ1 complexes, we sought to investigate the complexes created between nidogen-1 and laminin variants γ1 LEb2–4, γ1 LEb2–4 N836D, γ1 short arm, and γ1 short arm N836D by applying a combination of chemical cross-linking and high-resolution nano-HPLC/nano-ESI-LTQ-Orbitrap mass spectrometry. In the laminin γ1 short arm and γ1 LEb2–4 N836D point mutants, the Asn residue, which is crucial for nidogen binding, was exchanged for an acidic Asp. The obtained distance constraints were then used to identify previously unknown nidogen/laminin interfaces, to generate 3D-structural models of all their individual domains, and to generate a refined model of the high-affinity nidogen-1/laminin γ1 binding site based on the known 3D structure. In addition, SPR and ELISA assays allowed us to derive kinetic constants of the nidogen-1/laminin γ1 interaction. Together, the data presented herein shed new light on the mechanisms underlying nidogen/laminin interaction.

### Deriving Kinetic Constants of the Nidogen/Laminin Interaction

The binding affinities between nidogen-1 and laminin γ1 short arm and γ1 LEb2–4 were investigated with surface plasmon resonance spectroscopy by measuring single-cycle kinetics [36]. Representative examples of the obtained sensorgrams are depicted in Figure S5A. Apparent K_d_ values were determined to be in the (sub-)nanomolar range, confirming high binding affinities between nidogen-1 and laminins (Table S5). However, the nidogen-1 binding activity of laminin γ1 short arm variant was found to be one order of magnitude lower than that of laminin γ1 LEb2–4, which is almost exclusively caused by differences in the association phase. Nidogen-1 binding was also investigated for N836D variants of laminin γ1 short arm and γ1 LEb2–4. When analyte concentrations were increased ca. 100-fold compared to the laminin γ1 ‘wild-type’ variants, signals were detected for nidogen-1/laminin γ1 LEb2–4 N836D binding (Figure S5A), which, however, did not allow to derive kinetic constants. For nidogen-1 and laminin γ1 short arm N836D, no interaction was detected by SPR.

In addition, the binding affinities between nidogen-1 and laminin γ1 short arm, γ1 LEb2–4 and their respective N836D variants were investigated by ELISA-based binding assays (Figure S5B). These assays revealed no differences in binding affinities of the laminin γ1 short arm and the LEb2–4 fragment to nidogen-1. Both interactions showed strong binding with apparent K_d_ values of 1.1 and 1.4 nM (Table S5). Binding of the respective N836D variants to nidogen-1 showed a considerable loss of binding affinity with apparent K_d_-values of 34 and 45 nM (γ1 LE2–4 N836D and γ1 short arm N836D, respectively).

### Cross-Linking of Nidogen-1/Laminin γ1 Complexes

**Figure 2:**
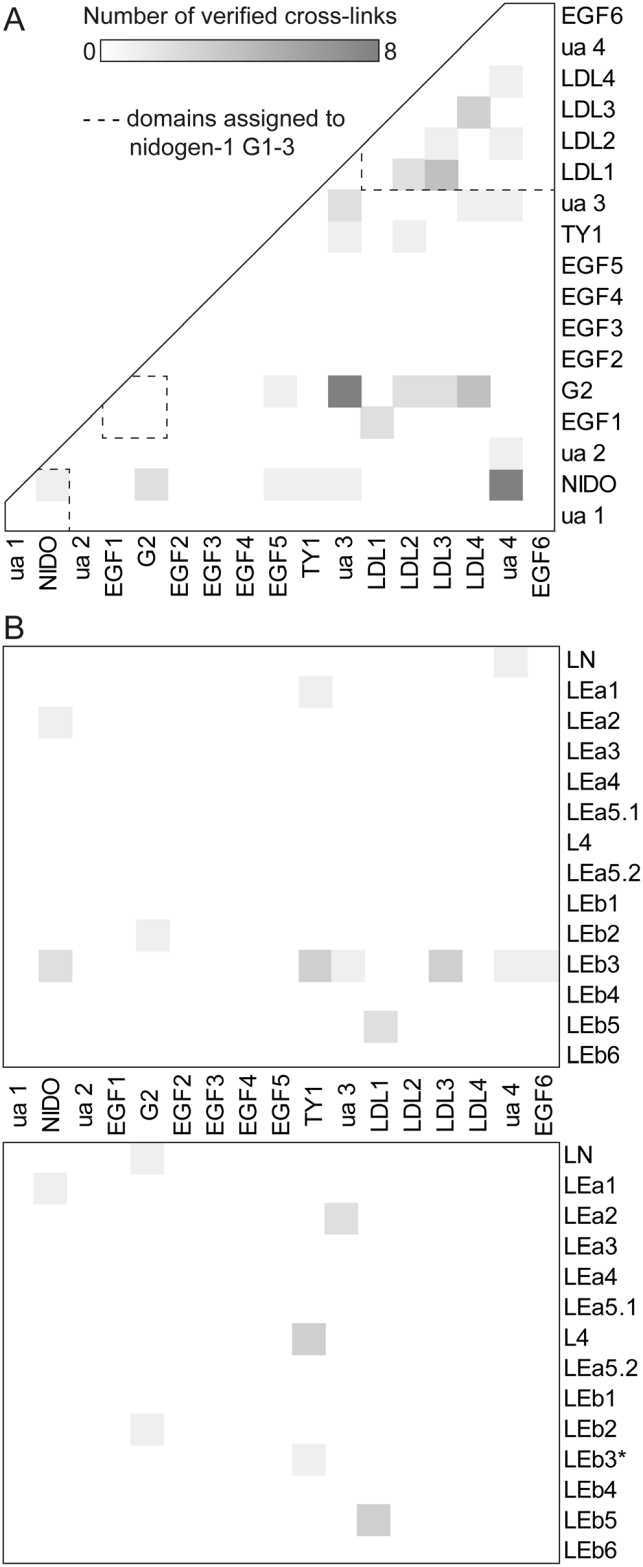
Diagonal plots of all cross-links identified. Cross-links are assigned to domains based on the UniProt KB entry P10493 (nidogen-1) and the laminin nomenclature of Aumailley *et al.* [5]. Unannotated areas within the sequences are named ‘ua’. Corresponding to the number of inter-domain contacts, areas of intersection are color-coded from white (none) to dark grey (maximum). (A) Intramolecular cross-links within nidogen-1. The globular domains G1, G2, and G3 are denoted by dotted lines. Cross-links located nearby the diagonal border represent contacts between domains being close to each other in the protein sequence. (B) Cross-links between nidogen-1 and laminin γ1 wild type (upper panel) as well as N836D variants (lower panel). The LEb3 domain, bearing the N836D mutation, is marked with an asterisk.

For gaining insights into the interaction between nidogen-1 and laminin γ1 variants on the molecular level, the proteins were cross-linked using the homobifunctional cross-linker BS^2^G. Additionally, we pursued a complementary approach by incorporating the unnatural diazirine-containing amino acids photo-Met and photo-Leu instead of methionine and leucine [58] into nidogen-1, laminin γ1 LEb2–4, and laminin γ1 short arm. MS/MS analysis of the non-cross-linked proteins revealed 13–25% of all leucines and methionines to be partially replaced by their photo-reactive counterparts. After UV-A-induced or BS^2^G-mediated cross-linking, the reaction mixtures were separated by SDS-PAGE and analyzed by LC/MS/MS. Experiments were conducted in the presence of varying laminin concentrations to optimize the efficiency of heterodimer formation between nidogen-1 and laminin. The verified cross-links are summarized in Figure 2 and in Tables S6 and S7.

We confirmed 47 intramolecular BS^2^G cross-links within nidogen-1, delivering 26 non-redundant distance constraints. Although the efficiency of photo-amino acid incorporation at the partially modified Leu and Met sites was moderate (∼35% for photo-Met, ∼3% for photo-Leu, see Figure S6), we identified two additional non-redundant photo-cross-links within nidogen-1, one connecting the link region with the G3 domain and one within the G3 domain, highlighting the sensitivity of our cross-linking/MS approach.

**Figure 3:**
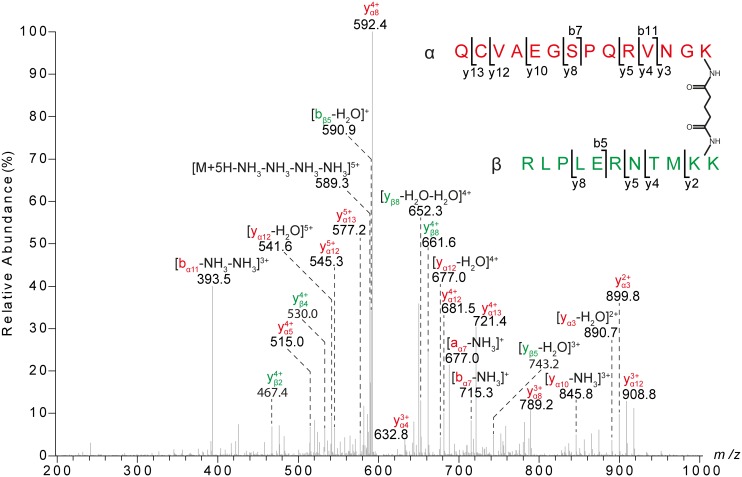
Nano-HPLC/nano-ESI-LTQ-Orbitrap-MS/MS analysis of cross-linked peptides derived from nidogen-1 G2 and G3 domain. The cross-linked product comprises amino acids 407–420 of the G2 domain (α-peptide, red) and 939–949 of the G3 domain (β-peptide, green), in which K-407 is connected to K-948/949.

More than half of the intermolecular cross-links were inter-domain contacts, in which all globular nidogen-1 domains were connected with each other (Figure 2A). The majority of distance constraints were found in the G3 domain as well as between G2 and G3 domains, while the ‘link’ region (ua 2) and EGF domains 2–4 within the ‘rod’ region were not involved in any cross-link. As an example, the fragment ion mass spectrum representing a cross-link between the G2 and the G3 domain of nidogen-1 is shown in Figure 3.

Ten out of 19 non-redundant BS^2^G cross-links between nidogen-1 and laminin γ1 were exclusively found in experiments with laminin γ1 wild type, six only in experiments with N836D variants, and three for both proteins. Additionally, one intermolecular contact between nidogen-1 G3 and laminin γ1 LEb3 was reproducibly identified in two consecutive photo-cross-linking experiments. With the exception of the link region, all nidogen-1 domains were involved in intermolecular cross-links with the nidogen binding motif of laminin γ1 (LEb2–4) as well as with additional regions within laminin γ1 short arm (Figure 2B). However, when laminin γ1 LEb2–4 N836D variants were used as interaction partners, the distribution of cross-links changed considerably with only two nidogen-1/laminin γ1 LEb2–4 contacts being identified (Figure 2C). Instead, L4 and LEb5 domains were repeatedly found to be cross-linked to nidogen-1.

Examining the intramolecular laminin γ1 cross-links underpins the complementarity of the two cross-linking strategies applied. Whereas only one BS^2^G cross-link within laminin γ1 variants was identified (LEb2 with LEb5), UV-A irradiation revealed connections of the L4 domain to LEa1 and LEa2 domains as well as one contact between the LEa4 and LEb6 domains. Notably, we exclusively identified cross-links between sequentially non-adjacent laminin domains.

### Structural Characterization of Laminin γ1 and Nidogen-1 Domains by Comparative Modeling

Structures of the laminin γ1 LEa3–5.2, LEb1, and LEb5 domains as well as for the nidogen-1 domains EGF2–6 and TY1 were derived by comparative modeling (Figure S7). Template structures were identified by sequence homology search using DELTA-BLAST. Since we did not observe cross-links within these domains only known disulfide linkages served as distance constraints. ‘Laminin-type EGF-like’ (LE) domains exhibit a characteristic disulfide linkage pattern that is different from classical EGF-like domains [21], [24], [33], [34]. Hence, only crystal structures of LE domains were used as templates for the remaining LE modules and nidogen-1 EGF domain models were based on the structures of classical EGF-like repeats.

**Figure 4:**
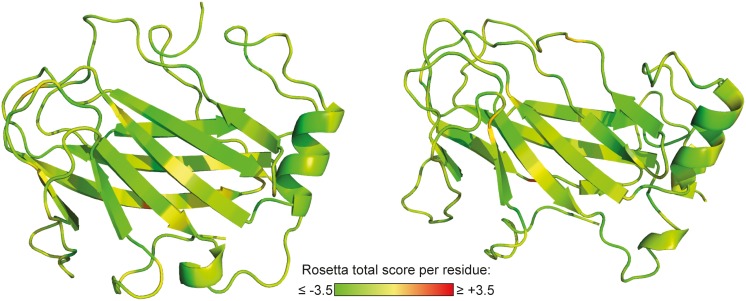
Structural models for laminin γ1 L4. Shown are the two best-scoring models generated by comparative modeling based on 13 structural homologues that have been identified by fold recognition. The residues are colored according to their Rosetta total score. Scores below zero (yellow-green color) indicate energetically favorable conformations.

Structural templates for the laminin γ1 L4 domain were identified by fold recognition as described in ‘Materials & Methods’. A topology dominated by ß-sheets was predicted by PSIPRED [47] (Figure S8A) and JUFO9D [48]. In compliance with this prediction, 13 potential templates with a common ß-sandwich topology were identified by the applied threading servers (Figure S8B). Comparative modeling of laminin γ1 L4 was thus based on these structural homologues. The best 10% of all generated models were clustered and further validated by generating score-vs-RMSD plots, assuming each of the best-scoring models of the top five clusters as the native structure (RMSD = 0). Convergence of the models towards a minimum Rosetta total score and RMSD underpins the validity of the structures with the lowest score (Figure S8C). The two final models, both adopting a galactose-binding domain-like fold, match the secondary structure predictions for the L4 domain and exhibit energetically favorable residue conformations throughout the structure (Figure 4).

### Generating an *Ab Initio* Model of the Nidogen-1 G1 Domain

**Figure 5:**
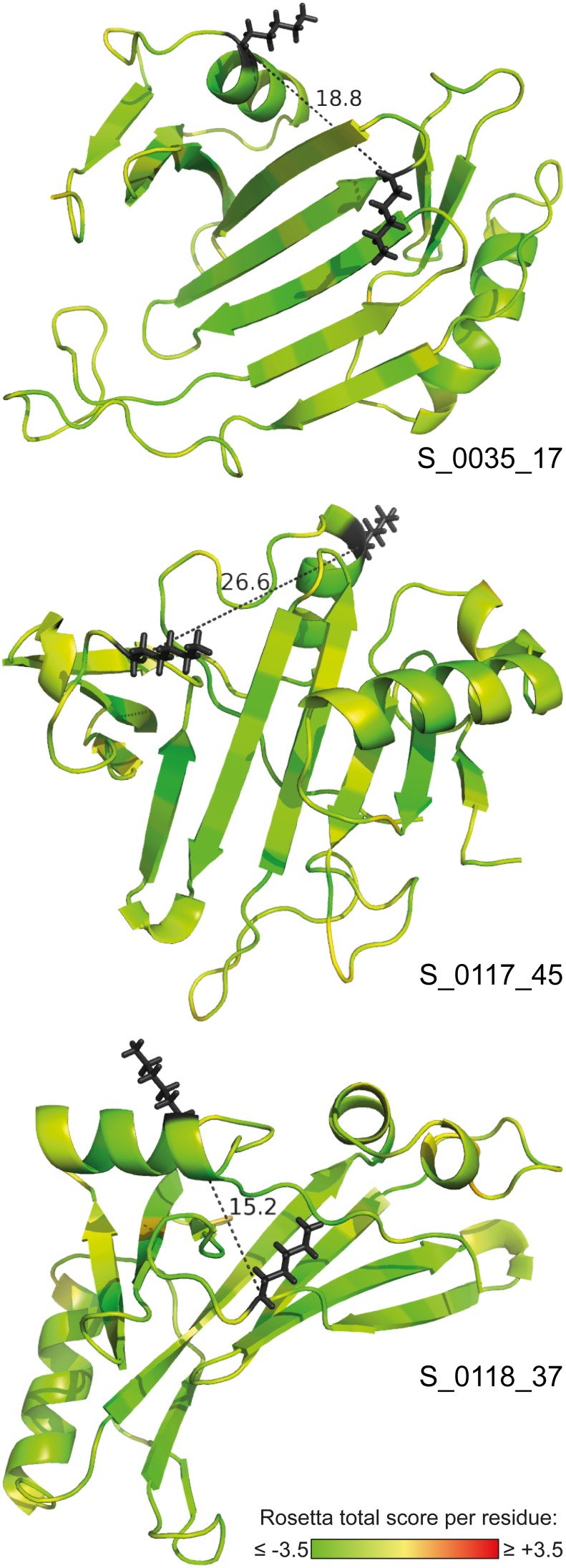
*De novo* folded models of nidogen-1 G1. The ten best-scoring structures among the final models were all derived from four initial centroid models of the G1 domain. Structures representing three of these centroid models are shown here. These models comply with the single distance constraint in this region that was identified by cross-linking/MS. Cross-linked residues are displayed as black sticks. Cα–Cα distances are given in Å. The residues are colored according to their Rosetta total score. Scores below zero (yellow-green color) indicate energetically favorable conformations. The identifiers of the underlying centroid models are indicated.

G1, the *N*-terminal globular region of nidogen-1, essentially consists of a NIDO domain (Figure 1). As neither sequential nor structural homologues were identified for nidogen-1 NIDO we decided for a *de novo* folding strategy. This approach is exceptionally challenging because of NIDO’s size (156 amino acids). Thus, we could not only rely on the Rosetta scoring function, but had to pursue alternative validation strategies [59]. First, we sought to identify known protein folds among the generated models by comparing them to a PISCES library of diverse PDB structures using MAMMOTH structural alignment (see ‘Materials & Methods’). Second, we reasoned that the topologies being sampled during *de novo* folding can be substantially influenced by subtle changes in the protein sequence. However, folding of highly similar sequences should result in identical tertiary structures. Similar topologies, sampled for several closely homologous sequences, are thus more likely to resemble the native structure. Therefore, we performed *de novo* folding not only for the murine NIDO domain, but also for homologues from six additional organisms with sequence identities larger than 75%. Taken together, these strategies resulted in the identification of 132 candidate structures after full-atom refinement and clustering (Table S3) and the ten top-scoring models were found to originate from four initial NIDO centroid models. Three of these models were validated by one BS^2^G cross-link, which had been identified within the NIDO domain, but was not used as a distance constraint during the modeling process. The determined Cα–Cα distances were within the range of the cross-linker, as shown in Figure 5. Additionally, a coarse clustering (radius = 10 Å) was performed to check whether further final candidate structures can be traced back to common centroid models. In view of the variety of conformations being sampled during the generation of centroid models, clusters were merged, when their best-scoring member structures originated from the same centroid model. We identified six additional centroid models that are represented by more than three full-atom refined models among the 132 final candidate structures (Table S8). For two of these centroid models, structural homologues in the PISCES PDB library have been identified. The best-scoring full-atom structural models for the six centroid models are shown in Figure S9. In all these NIDO models the cross-linking distance constraint is fulfilled. Taken together, our *de novo* approach suggests that NIDO adopts a compact topology containing a ß-sheet with at least four strands and two α-helices.

### Incorporation of Cross-Linking Distance Constraints into the Nidogen-1 G3/Laminin γ1 LEb2–4 Core Structure

**Figure 6:**
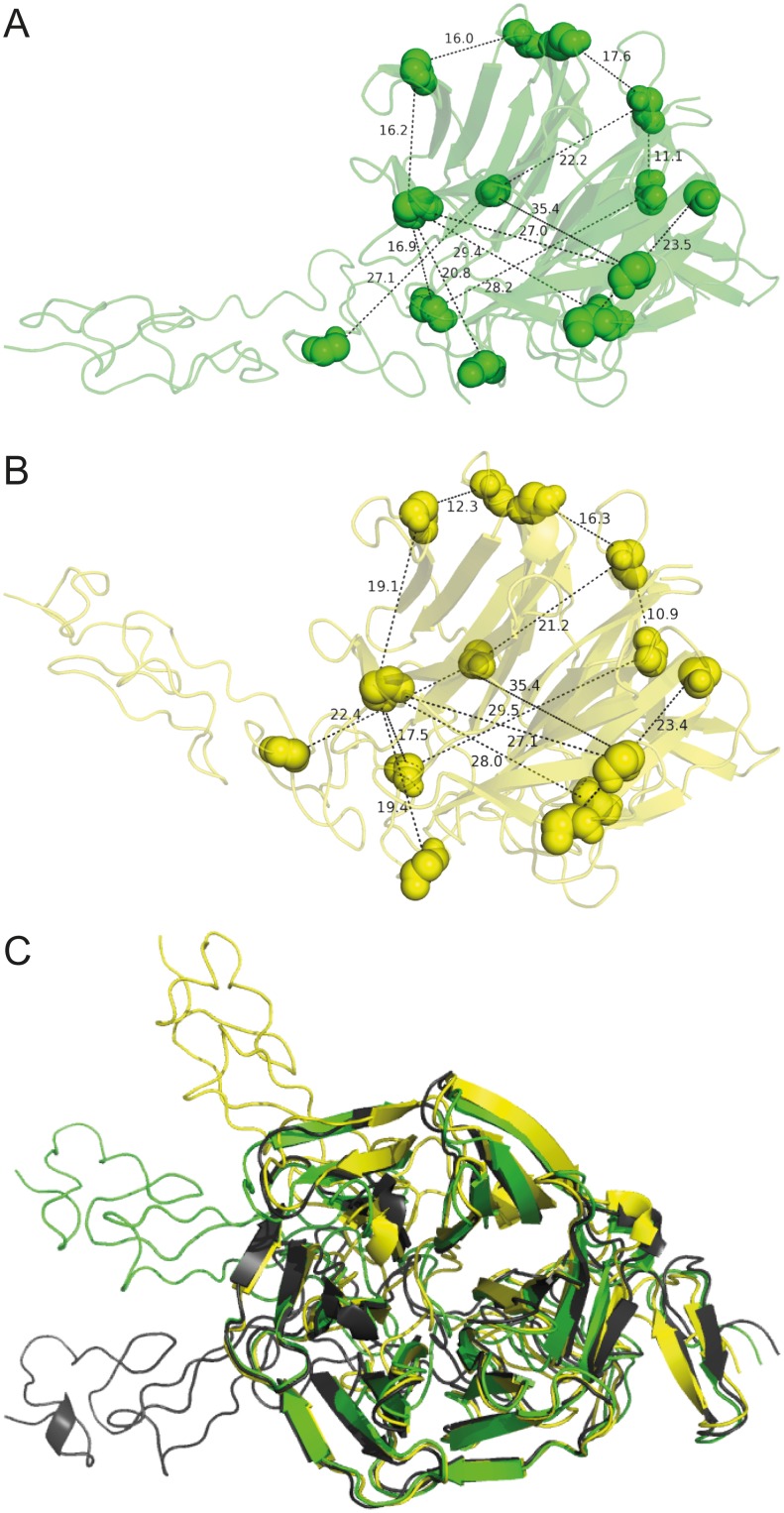
Refined models of the nidogen-1 G3/laminin γ1 LEb2–4 complex. Based on PDB entry 1NPE and the identified cross-links, modified structural models for the high-affinity interaction region of laminin γ1 and nidogen-1 were generated. Cross-linked residues are displayed as spheres. Cα–Cα distances are given in Å. (A) Model with the best Rosetta total score and a Rosetta atom-pair constraint score ranking among the top 2.5%. (B) Model with the best Rosetta atom-pair constraint score and a Rosetta total score ranking among the top 2.5%. (C) Alignment of both models and the unmodified crystal structure 1NPE (black). The orientation of LEb2–4 clearly has changed during structural refinement. The β-propeller fold of the G3 domain is still intact.

Eleven BS^2^G cross-links originate from the known interaction region between the LEb2–4 domains of laminin γ1 and the G3 domain of nidogen-1. The distance constraints obtained by our cross-linking experiments should thus be in agreement with the known 3D structure of the nidogen-1 G3/laminin γ1 LEb2–4 complex (PDB entry 1NPE) [24]. According to the spacer arm length of BS^2^G (7.5 Å [55]) and the length of the cross-linked lysine side chains (2×6.3 Å), the maximum Euclidian Cα–Cα distance between the cross-linked residues should be 20.1 Å. However, longer distances are frequently observed, when cross-links are mapped in structural models. Therefore, it is common practice to grant a distance tolerance of 6–7 Å to account for structural flexibility. Recently, a rationale for this approach was presented by studying the lysine–lysine distances within 807 proteins during molecular dynamics simulations [60]. This analysis gave evidence that the ε-amino groups of lysines, with initial Cα–Cα distances of up to 38 Å, will move inside the range of the amine-reactive cross-linker DSS (spacer arm length 11.4 Å) during a 50 ns-simulation. Considering the shorter spacer arm length of BS^2^G, the maximum Euclidean Cα–Cα distance that is likely to allow cross-linking can, therefore, be estimated to 34 Å. In an earlier study, similar results were obtained by cross-linking seven model proteins and comparing the identified cross-links with their respective crystal structures [61]. Mapping our cross-links into the X-ray structure of the nidogen-1 G3/laminin γ1 LEb2–4 complex resulted in Cα–Cα distances between 10.4 and 35.8 Å. By integrating the crystal structure and our cross-linking data in a Rosetta-based modeling approach, we aimed to derive structural models that better reflect plausible in-solution conformations of the nidogen-1 G3/laminin γ1 LEb2–4 complex, which would be signified by a decrease in observed Cα–Cα distances. The best-scoring models of the complex are depicted in Figure 6. The laminin γ1 fragment is considerably bent compared to the X-ray structure (Figure 6C), which is conceivable as the three LE repeats do not form a compact tertiary structure around a defined hydrophobic core. Both the disulfide bond pattern of the LE domains and the β-propeller structure of the G3 domain are well maintained indicating that the structural rearrangements in our models are reasonable. Notably, the spanned Cα–Cα distances of all except one BS^2^G cross-link are significantly reduced compared to the X-ray structure, suggesting that the models give a better picture of the conformations the nidogen-1 G3/laminin γ1 LEb2–4 complex can adopt in solution (Table 1). However, one has to be aware that the Rosetta models, just as the crystal structure, represent conformational samples and do not reflect the entire conformational space of the protein complex.

In this context, it has to be pointed out that the single cross-link that exceeds the 34 Å distance limit of the X-ray structure is in conflict with the models. Visual inspection of the structural models shows that the respective lysines cannot be cross-linked, since the spacer arm would have to traverse through the center of the β-propeller, which is blocked by residues of the G3 domain. We interpret this cross-link as an intermolecular contact between two nidogen-1 molecules. Forcing Rosetta to fulfill this distance constraint resulted in unfolding of the G3 domain, confirming that a sound model cannot be forced to match the experimental data, but complies only with sterically feasible cross-links. In addition to the BS^2^G cross-links we obtained two distance constraints by photo-cross-linking. The respective Cα–Cα distances of the cross-links, in which photo-Leu is connected to Arg and Lys, were 24.7 and 33.7 Å within the X-ray structure as well as 23.4–23.5 Å and 19.4–20.8 Å within the models (Table 1). This is well above the expected maximum values of 10.4 Å for a photo-Leu/Lys cross-link and 11.4 Å for a photo-Leu/Arg cross-link, which were determined from the side chain lengths within representative amino acid crystal structures deposited in the Cambridge Structural Database [62]. However, in both cases, one of the cross-linked amino acids is located in a loop region. It is conceivable that the obtained Cα–Cα distances are longer than the maximum expected distances as the loop regions are flexible. Granting a similar distance tolerance as for BS^2^G results in maximum allowed Cα–Cα distances of 23.4 Å and 24.4 Å, respectively, both of which are met by our models. Intriguingly, the cross-link between photo-Leu-844 (laminin) and Lys-1072 (nidogen- 1) concurs with a BS^2^G cross-link pointing to the same region (laminin Lys-850 with nidogen-1 Lys-1072).

## Discussion

The primary goal of this work was to gain novel insights into the nidogen-1/laminin γ1 interaction in solution by combining amine-reactive and photo-chemical cross-linking with high-resolution mass spectrometry and computational modeling. Additionally, we probed the affinity of nidogen-1 to different laminin γ1 variants by means of SPR and ELISA-based binding assays.

So far, quantitative analyses of the nidogen-1/laminin affinity have only been performed using the laminin P1 fragment, which is produced by limited pepsin proteolysis and comprises parts of all three chains (α1, β1, γ1) of laminin-111 [9], [11], [13], [63]. Apparent K_d_ values determined in those studies ranged between 0.5 nM and 1 nM, complying with the dissociation constants derived from the ELISA assays (Table S5). SPR analysis yielded similar results for the nidogen-1/laminin γ1 LEb2–4 interaction, but a lower apparent K_d_ value (∼12 nM) for laminin γ1 short arm. In the SPR experiments, nidogen-1 was immobilized in random orientations by covalently linking lysine residues to the sensor chip surface, while the laminin variants were used as mobile analytes. Notably, the lower affinity of laminin γ1 short arm is solely caused by a slower association – the dissociation rate constant is similar for both laminin variants (Table S5). Therefore, we conclude that the deviations in the SPR experiments are caused by different *in solution* properties of laminin γ1 short arm compared to laminin γ1 LEb2–4. This hypothesis is supported by the ELISA experiments where nidogen-1 was used as ligand, while the laminin variants were immobilized through passive adsorption in a 96-well plate [64], resulting in similar nidogen-1-binding affinities. These different experimental setups may introduce different degrees of steric hindrance that probably do not affect the binding of the relatively small laminin γ1 LEb2–4 fragment (25 kDa), but hamper the interaction with the laminin γ1 short arm variant, which is considerably bulkier (113 kDa). In other words, the laminin γ1 short arm molecules might not be equally binding-competent due to different *in solution* conformations. This results in a slower association and consequently, a lower apparent K_d_ value when using laminin γ1 short arm as mobile analyte.

Moreover, interactions between nidogen-1 and laminin γ1 N836D variants were verified by both affinity assays. The ELISA results suggest a ∼30-fold loss in affinity upon mutating Asn-836 to aspartic acid. This finding is in contrast to a previous study by Pöschl *et al.* who found this mutation to cause a 25,000-fold loss in affinity, practically abolishing any interaction [23]. Our data suggest a much less dramatic influence of laminin γ1 Asn-836 on nidogen-1 binding.

The large number of inter-domain contacts verified for nidogen-1 implies a globular conformation rather than a linear domain arrangement. This is in agreement with a previous structural investigation of nidogen-1 by electron microscopy showing a wide range of conformations, including both linear as well as globular structures [13]. This variability within nidogen-1 might be caused by high flexibility of the elongated regions connecting the globular domains (‘link’ and ‘rod’), which could likewise be an explanation for the almost complete lack of cross-links in these regions (Figure 2A). Within laminin γ1 short arm variants, intramolecular contacts were exclusively found between non-adjacent domains supporting a compact globular protein architecture as well. The cross-links also give hints on the existence of an ensemble of defined conformations for both nidogen-1 and laminin γ1 short arm.

**Figure 7:**
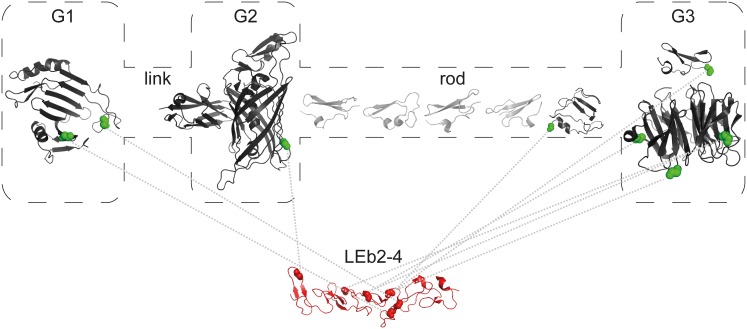
Contacts of laminin γ1 LEb2–4 wild type with nidogen-1. The LEb2–4 structure (red) is taken from PDB entry 1NPE. Nidogen-1 (grey) is schematically depicted as a combination of the crystal structures 1GL4 (G2 domain) and 1NPE (G3 domain) and representative models of the remaining domains. Residues involved in intermolecular contacts are shown as spheres. Gray dotted lines represent verified cross-links.

The intermolecular cross-links between nidogen-1 and the laminin variants strongly indicate additional interaction regions next to the known nidogen-1 G3/laminin γ1 LEb2–4 binding site. Even laminin γ1 LEb2–4 was found to form contacts to all globular nidogen-1 domains and the rod region (Figure 7). We conclude that our cross-linking experiments allowed us to pick up different nidogen-1/laminin γ1 complexes that are present in solution, while the published X-ray structure reflects only one interaction ‘snapshot’ − most likely the best crystallizable conformation.

The existence of additional interaction patterns was further substantiated by cross-linking experiments using nidogen-1 and laminin γ1 N836D variants, which were unanimously shown to interact regardless of the N836D substitution within the laminin γ1 LEb3 domain. Interestingly, contacts of nidogen-1 to LEb3 were almost completely abolished, while alternative laminin γ1 domains were found to be cross-linked to nidogen-1 (Figure 2). Taken together, these results suggest alternative nidogen-1/laminin γ1 binding modes, conceivably as a result of the conformational flexibility of both proteins, which had already been implied by electron microscopy [65]. When performing cross-linking experiments, one has to keep in mind that amine-reactive cross-linking depends on the reactivity of lysines, which is influenced by solvent exposure of their side chains and local pK_a_ values [66]. Considering the spacer length of BS^2^G, the observation of an intermolecular cross-link is not *per se* equivalent to physical binding at exactly that site. While our cross-linking results indeed indicate additional binding sites, it is still imaginable that laminin γ1 LEb2–4 remains the primary anchoring site of nidogen-1. In fact, solid phase binding studies have proven that the nidogen-1 G3 domain is essential for nidogen-1/laminin γ1 interaction [63], [67]. Therefore, nidogen-1 G3/laminin γ1 LEb2–4 binding is most likely crucial for a high-affinity interaction and binding at alternative sites proceeds with substantially lower affinity.

This finding seems reasonable also in view of the stabilizing function of nidogen-1 within basement membranes, connecting the laminin and the type IV collagen network [14]. Given the mechanical stress basement membranes have to withstand [2], secondary interactions at alternative binding sites may occur when the basement membrane is in a more relaxed state. In contrast, the high-affinity anchoring interaction between nidogen-1 G3 and laminin γ1 LEb2–4 is likely to be continuously present, thereby ensuring a high mechanical stability. Although this binding region represents one of the smallest high-affinity interfaces known so far [24], our SPR, ELISA, and cross-linking/MS data indicate a certain robustness against the single point mutation N836D, which is plausible in light of the physiological importance of the nidogen/laminin interaction [68].

In line with these findings, we were not able to fit all cross-linking distance constraints for nidogen-1 and laminin γ1 into one single model of the protein complex. Assigning the cross-links to defined conformations within an ensemble of co-existing structural arrangements is currently beyond the bounds of the method. Consequently, a computational model of the entire nidogen-1/laminin γ1 short arm complex based on cross-linking distance constraints would be overly speculative. Comparative modeling and *de novo* folding using the Rosetta modeling suite, however, enabled us to create models of all nidogen-1 and laminin γ1 short arm domains that have not been structurally characterized so far. The structural models of laminin γ1 L4 and nidogen-1 NIDO are of particular interest as there are not any sequential homologues with known structures. We were able to identify structural homologues of laminin γ1 L4 suggesting a galactose-binding domain-like fold of this domain. Interestingly, this fold is also adopted by the *N*-terminal LN domains of laminin α5, β1, and γ1 [7], [33], [34]. To date, experimental evidence for carbohydrate-binding activity of laminins only exists for *C*-terminal laminin α LG domains [69].

Structures of the nidogen-1 NIDO domain were obtained by *de novo* folding. The proposed models either exhibit structural similarities to known PDB structures or were consistently modeled based on six NIDO sequences of evolutionary closely related organisms. All but one of the final models were further validated as they fulfill the only cross-linking constraint identified in this region. These models exhibit a compact topology with a β-sheet surrounded by two α-helices as central elements.

Finally, integrating the cross-links found within the nidogen-1 G3/laminin γ1 LEb2–4 complex and the known X-ray structure confirms the main structural features suggested by X-ray crystallography, yet indicating a more bent topology of the LEb2–4 domains (Figure 6C). Again, this finding depicts the flexibility of laminin γ1 resulting in overlying conformations of the nidogen-1/laminin γ1 complex, which can be captured by chemical cross-linking and thus reflect the whole picture of nidogen-1/laminin γ1 interaction in solution.

## Conclusions

With our approach integrating chemical cross-linking, mass spectrometry, and computational modeling, we were able to structurally characterize conformations of nidogen-1/laminin γ1 complexes in solution. We applied two complementary cross-linking approaches, one using a classical homobifunctional amine-reactive cross-linker, the other one relying on the incorporation of unnatural photo-reactive amino acids.

Cross-links between laminin γ1 short arm and nidogen-1 were found in all protein regions. Therefore, it is likely that both proteins exhibit several additional contact regions apart from their known interaction site. In addition, different modes of interaction resulting in several distinct protein-protein interfaces can be imagined. Our results indicate that Asn-836 within laminin γ1 LEb3 domain is not essential for complex formation. Conclusively, this work describes the first structural insights into the conformational dynamics of the nidogen-1/laminin γ1 complex and provides, for the first time, structural models of all nidogen-1 and laminin γ1 short arm domains. Chemical cross-linking, MS, and computational modeling allowed elucidating different conformations of the nidogen-1/laminin γ1 complex, which exist simultaneously in solution but are not reflected in the X-ray structure.

## Supporting Information

Figure S1 **Amino acid sequences of (A) nidogen-1 and (B) laminin γ1 short arm.**
(TIF)

Figure S2 **Clustal W2.1 sequence alignments for comparative modeling.** Shown are pairwise sequence alignments of all nidogen-1 and laminin γ1 target sequences to template sequences with sequence identities ≥30%. The scheme for template sequences is termed ‘PDB-entry_domain-name_chain-identifier’. Annotations comply with the Clustal nomenclature with identical (*), conserved (:) and semi-conserved (.) residues being denoted.
(DOC)

Figure S3 **Manual sequence alignments for modeling of the laminin γ1 L4 domain.** Shown are pairwise sequence alignments to all template sequences. Alignments are manually optimized to obtain maximum overlap of secondary structure elements. The scheme for template sequences is termed ‘PDB-entry_chain-identifier’.
(DOC)

Figure S4 **Jalview sequence alignment of nidogen-1 NIDO domains from different organisms.** All NIDO domains share sequence identities >75% but exhibit short sequence stretches that are diverse.
(DOC)

Figure S5 **Probing the nidogen-1/laminin γ1 interaction with SPR and ELISA assays.** (A) Single-cycle kinetic experiments were performed by injecting mobile analyte (laminin) at increasing concentrations followed by partial dissociation. Initially, experiments were carried out with 6.25 nM, 12.5 nM, 25 nM, 50 nM and 100 nM laminin γ1 LEb2–4 wild type and N836D. Binding of laminin γ1 LEb2–4 N836D was additionally probed with 100-fold increased concentrations. System artefact signals (∼30 min after each injection) were removed from the sensorgrams. (B) ELISA assays were performed in 96-well plates with immobilized laminin γ1 variants. Nidogen-1 was added in increasing concentrations (0.03–234 nM) until saturation was reached (incubation time: 1 h). Error bars represent standard deviations.
(TIF)

Figure S6 **Incorporation efficiency of photo-amino acids into nidogen-1 and laminin γ1 short arm.** (A) Met and Leu variants that were considered during MS analysis, including the reaction products of the photo-amino acids identified in [35] (1: photo-Leu, 5: photo-Met, 2 and 6: alkene; 3 and 7: alcohol; 4: unmodified Leu; 8 and 9: unmodified and oxidized Met). (B and C) MS-based label-free quantification of photo-amino acid incorporation. The pie charts show the number of leucines (blue) and methionines (red) within nidogen-1 (B) and laminin γ1 short arm (C) that remained unmodified (light shades) or were partially replaced by their photo-reactive counterparts (dark shades). The bars represent the relative abundance of partially modified peptides, containing the Leu (blue) and Met (red) variants listed in (A) [70].
(TIF)

Figure S7 **Homology models of (A) nidogen-1 and (B) laminin γ1 short arm domains.** Alignments of the best-scoring models representing the top three clusters are shown. Disulfide bridges are depicted as black sticks. All models were generated based on X-ray structures sharing more than 30% sequence identity with the respective domains.
(TIF)

Figure S8 **Comparative modeling of laminin γ1 L4.** (A) PSIPRED secondary structure prediction for the L4 domain. A β-sheet-rich fold and one long α-helix are predicted. (B) MUSTANG alignment of 13 potential structural homologs of L4 identified by fold recognition using several threading servers. All template candidates exhibit a β-sandwich topology. The number of β-strands is in line with the predicted secondary structure of L4. Instead of an α-helix, all structures contain a long loop region. (C) Rosetta total score of the top 10% of all generated models plotted against their RMSD from the best-scoring structure. Only α-helices, β-sheets and short loops (≤5 residues) were included in RMSD calculations. The models are converging to a minimum in score and RMSD indicating that the best-scoring models are valid. The two best-scoring models shown in Figure 4 are marked with red circles.
(TIF)

Figure S9 **Best-scoring nidogen-1 NIDO models originating from common centroid models.** The full-atom candidate structures of the NIDO domain were examined for common initial centroid models. Next to the centroid models underlying the structures depicted in Figure 5, we identified six centroid models that form the basis for more than three full-atom refined candidate structures. Shown are the best-scoring final candidate structures representing these initial centroid models. The Cα–Cα distances corresponding to the cross-link located within the models are given in Å. The residues are colored according to their Rosetta total score. Scores below zero (yellow-green color) indicate energetically favorable conformations. The identifiers of the underlying centroid models are given.
(TIF)

Table S1 **Results of Rosetta clustering of comparative nidogen-1 and laminin γ1 domain models.** The best 10% of all generated models were clustered. The ideal clustering radius was automatically determined by the Rosetta algorithm. Shown are clusters with a size >1.
(DOC)

Table S2 **Results of Rosetta clustering of the laminin γ1 L4 domain models.** The best 10% of all generated models were clustered using a clustering radius of 1 Å. Shown are clusters with a size >1. The best-scoring models of clusters 1 and 2 were chosen as final models of the L4 domain.
(DOC)

Table S3 **Scores of the final nidogen-1 NIDO domain models.** Models sharing the ‘centroid model identifier’ originate from the same initial low-resolution centroid model. Models, for which structural homologues within the PDB have been identified, are listed in italics. The remaining models share a similar topology to models generated based on highly homologous sequences of NIDO domains derived from related organisms.
(DOC)

Table S4 **Scores of the final models of the nidogen-1 G3/laminin γ1 LEb2–4 complex.** The listed models rank among the top 20 of 800 generated models considering both Rosetta total score and atom pair constraint score, which reflects their compliance with the cross-linking distance constraints.
(DOC)

Table S5 **Affinities and kinetic parameters of the nidogen-1/laminin γ1 interaction.** For SPR measurements, given values for k_a_ and k_d_ are the weighted mean from two individual measurements and K_d_ was calculated from these values as K_d_ = k_d_/k_a_. All ELISA-based measurements were performed in triplicates and K_d_ values were determined by non-linear regression of the saturation binding curves. The values in parentheses represent standard deviations.
(DOC)

Table S6 **Verified products of BS^2^G-mediated cross-linking.** Peptide sequences written in parentheses are part of the protein affinity tags and do thus not belong to the native amino acid sequences of the proteins studied. Oxidized methionines within the peptide sequences are denoted with ‘m’. Loss of water or ammonia is indicated by addition of ‘−H_2_O’ or ‘−NH_3_’ to the fragment ion.
(DOC)

Table S7 **Verified products of UV A-induced cross-linking.** Peptide sequences written in parentheses are part of the protein affinity tags and do thus not belong to the native amino acid sequences of the proteins studied. For ambiguous cross-links, all potential cross-linked amino acids are listed. Within the peptide sequences, photo-leucine and photo-methionine are assigned with ‘z’ and ‘o’, respectively. Oxidized methionines are denoted with ‘m’. Loss of water or ammonium is indicated by addition of ‘−H_2_O’ or ‘−NH_3_’ to the fragment ion.
(DOC)

Table S8 **Rosetta clustering results of the final nidogen-1 NIDO domain models.** The clustering radius was set to 10 Å. Clusters represented by models originating from the same initial low-resolution centroid model were merged. Shown are clusters with more than three member structures. Models, for which structural homologues within the PDB have been identified, are listed in italics. The remaining models share a similar topology to models generated based on highly homologous sequences of NIDO domains derived from related organisms.
(DOC)

File S1 **Command line execution commands and flags used for computational modeling with Rosetta.**
(DOC)

File S2 **Rosetta loops files generated for comparative modeling.** The position of the loops was determined based on the sequence alignments of the target sequences (listed in italics) to the respective templates. The scheme for template sequences is termed ‘PDB-entry_domain-name_chain-identifier’.
(DOC)
