# Supplementary figures and images for: Analysis of Nidogen-1/Laminin γ1 Interaction by Cross-Linking, Mass Spectrometry, and Computational Modeling Reveals Multiple Binding Modes
Source: PLoS One. 2014 Nov 11;9(11):e112886. doi: 10.1371/journal.pone.0112886 (PMC4227867; doi:10.1371/journal.pone.0112886)

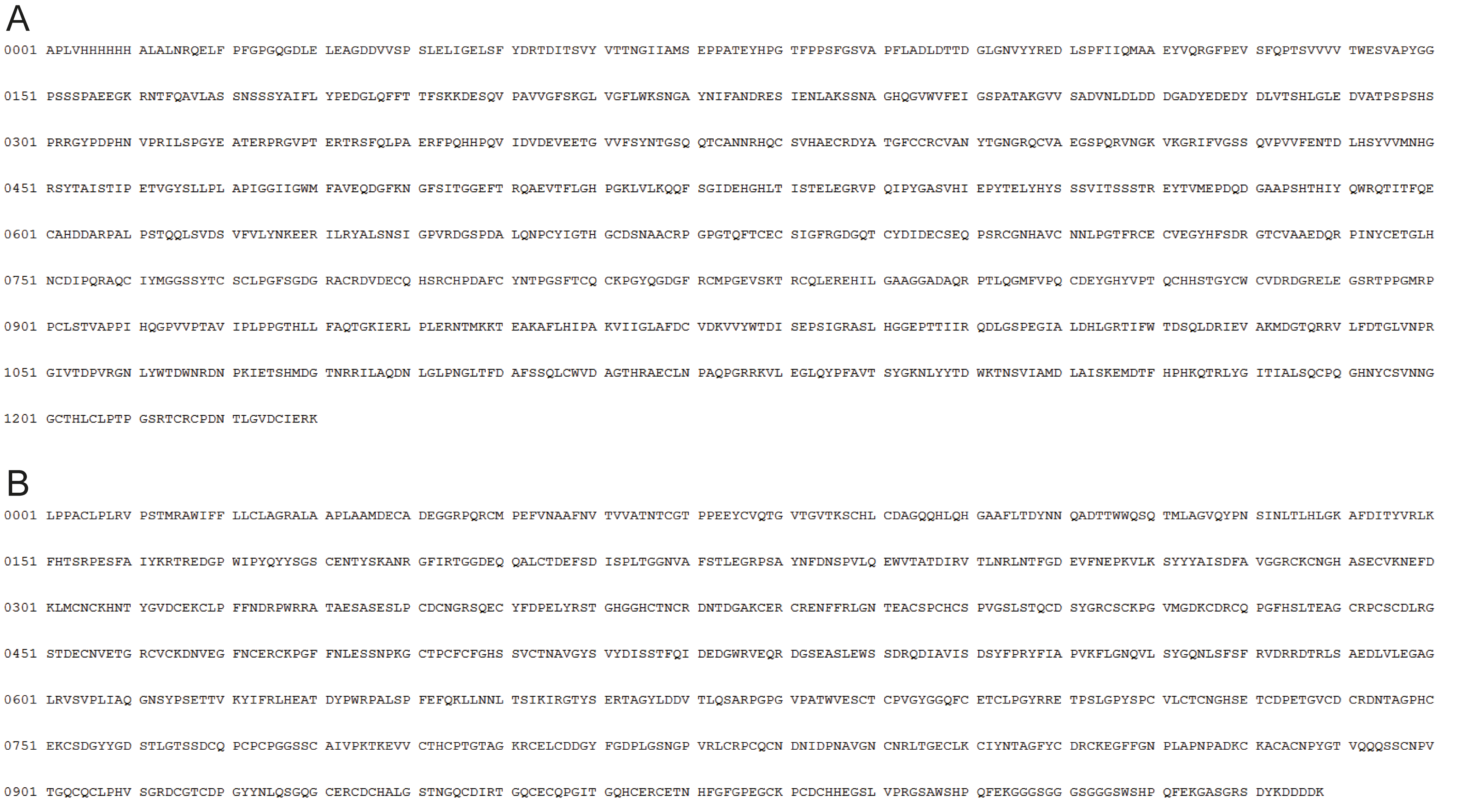

Supplement: Figure S1 — Amino acid sequences of (A) nidogen-1 and (B) laminin γ1 short arm. (TIF) [file pone.0112886.s001.tif]

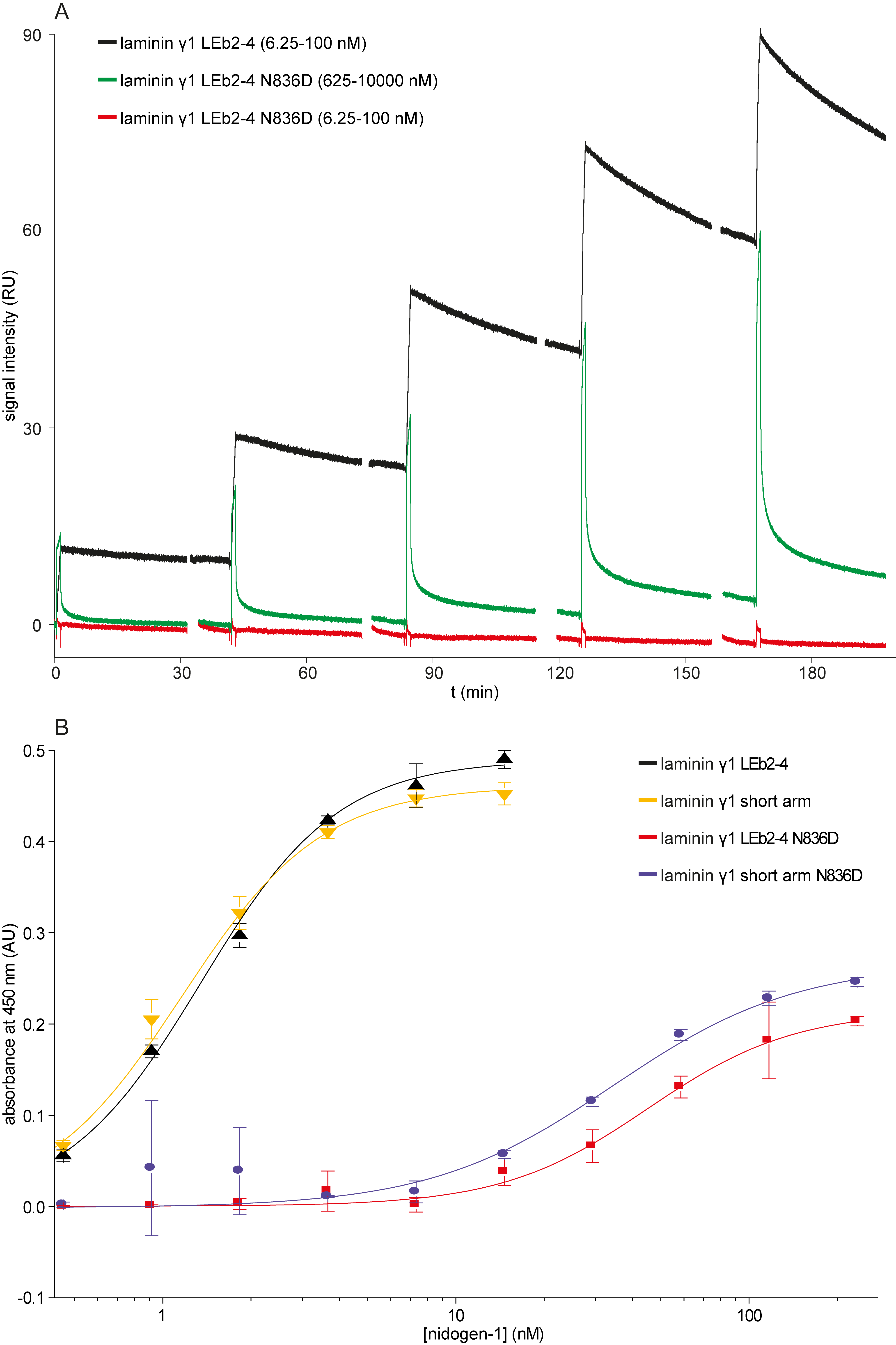

Supplement: Figure S5 — Probing the nidogen-1/laminin γ1 interaction with SPR and ELISA assays. (A) Single-cycle kinetic experiments were performed by injecting mobile analyte (laminin) at increasing concentrations followed by partial dissociation. Initially, experiments were carried out with 6.25 nM, 12.5 nM, 25 nM, 50 nM and 100 nM laminin γ1 LEb2–4 wild type and N836D. Binding of laminin γ1 LEb2–4 N836D was additionally probed with 100-fold increased concentrations. System artefact signals (∼30 min after each injection) were removed from the sensorgrams. (B) ELISA assays were performed in 96-well plates with immobilized laminin γ1 variants. Nidogen-1 was added in increasing concentrations (0.03–234 nM) until saturation was reached (incubation time: 1 h). Error bars represent standard deviations. (TIF) [file pone.0112886.s005.tif]

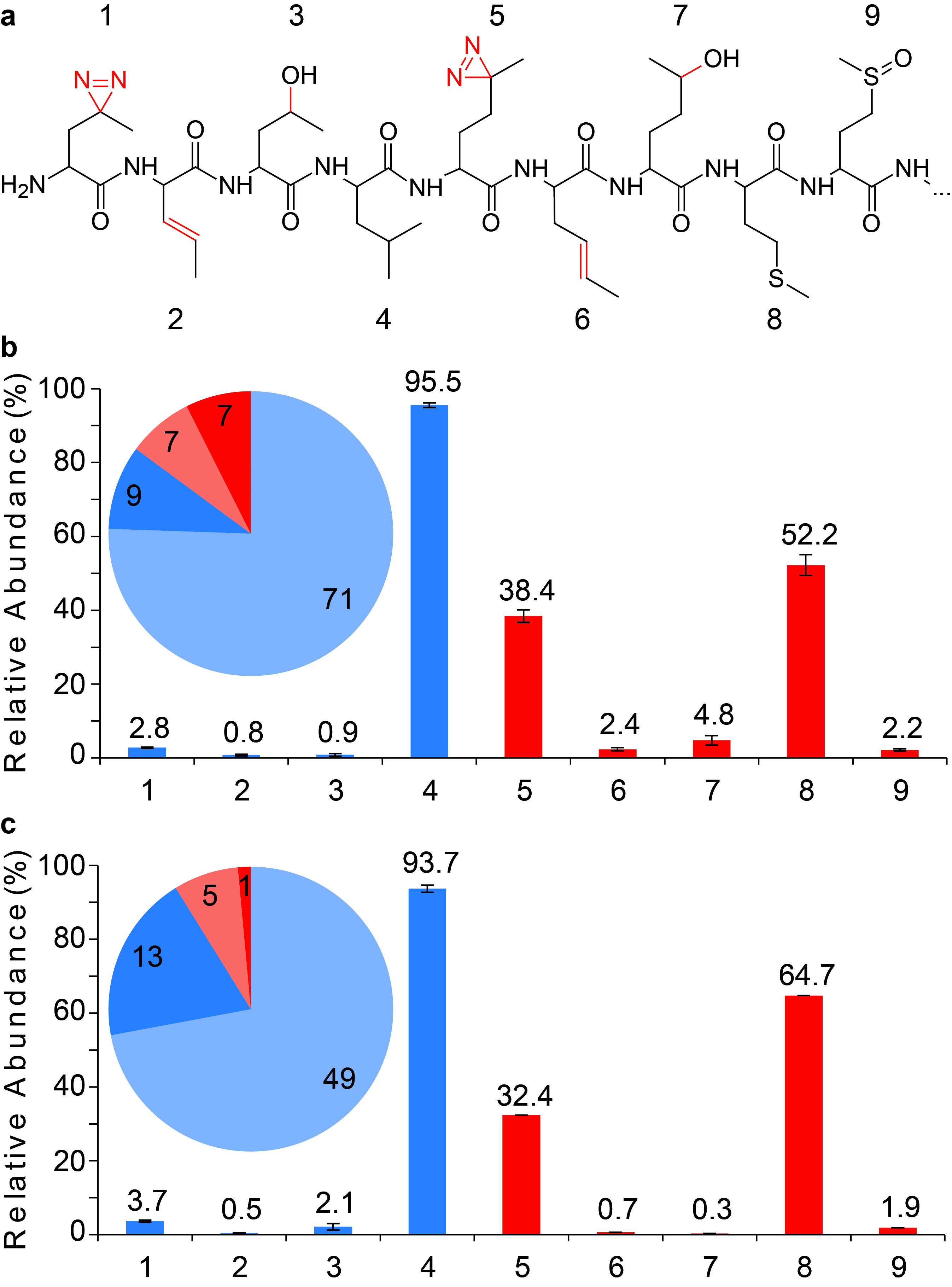

Supplement: Figure S6 — Incorporation efficiency of photo-amino acids into nidogen-1 and laminin γ1 short arm. (A) Met and Leu variants that were considered during MS analysis, including the reaction products of the photo-amino acids identified in [35] (1: photo-Leu, 5: photo-Met, 2 and 6: alkene; 3 and 7: alcohol; 4: unmodified Leu; 8 and 9: unmodified and oxidized Met). (B and C) MS-based label-free quantification of photo-amino acid incorporation. The pie charts show the number of leucines (blue) and methionines (red) within nidogen-1 (B) and laminin γ1 short arm (C) that remained unmodified (light shades) or were partially replaced by their photo-reactive counterparts (dark shades). The bars represent the relative abundance of partially modified peptides, containing the Leu (blue) and Met (red) variants listed in (A) [70]. (TIF) [file pone.0112886.s006.tif]

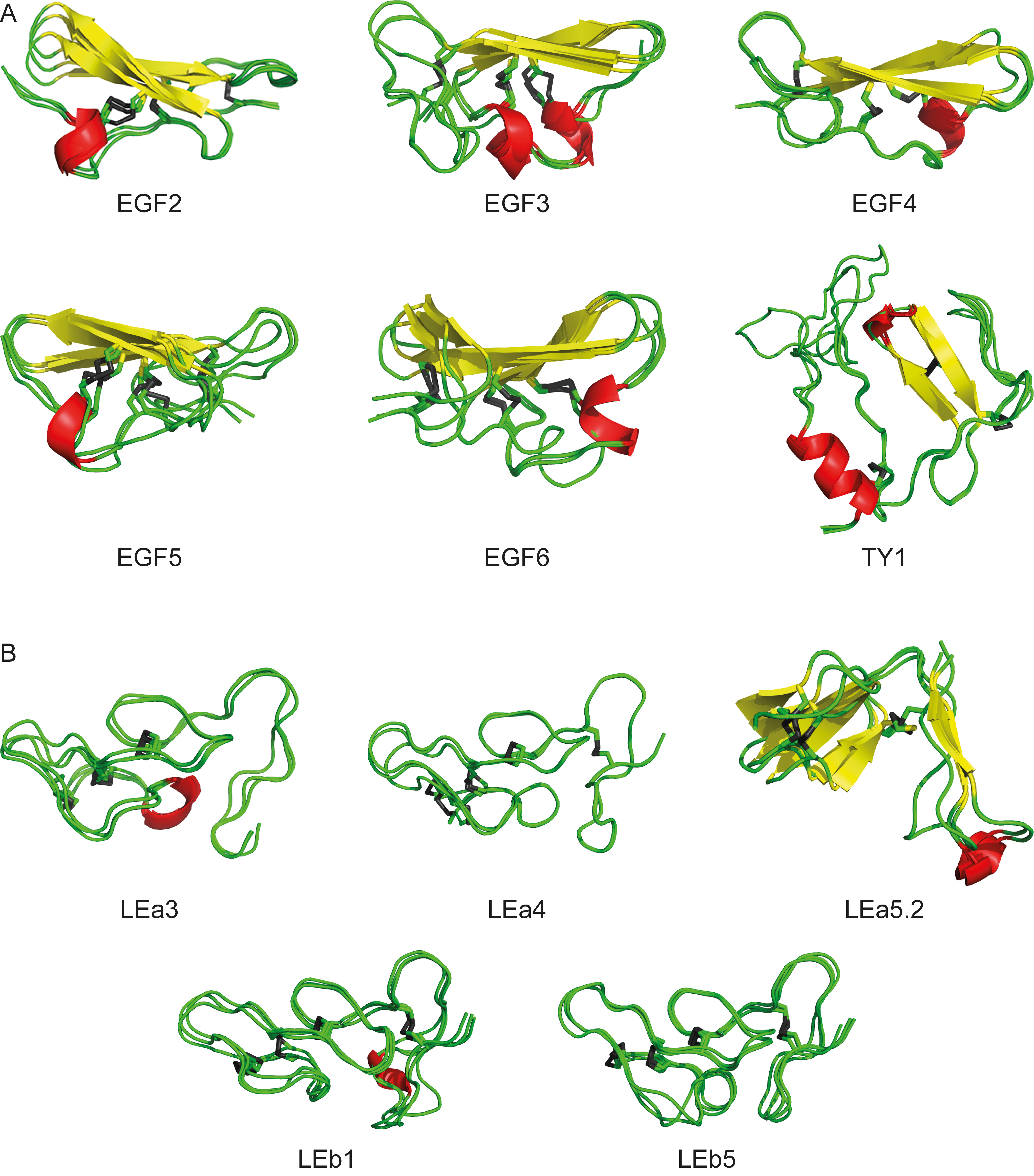

Supplement: Figure S7 — Homology models of (A) nidogen-1 and (B) laminin γ1 short arm domains. Alignments of the best-scoring models representing the top three clusters are shown. Disulfide bridges are depicted as black sticks. All models were generated based on X-ray structures sharing more than 30% sequence identity with the respective domains. (TIF) [file pone.0112886.s007.tif]

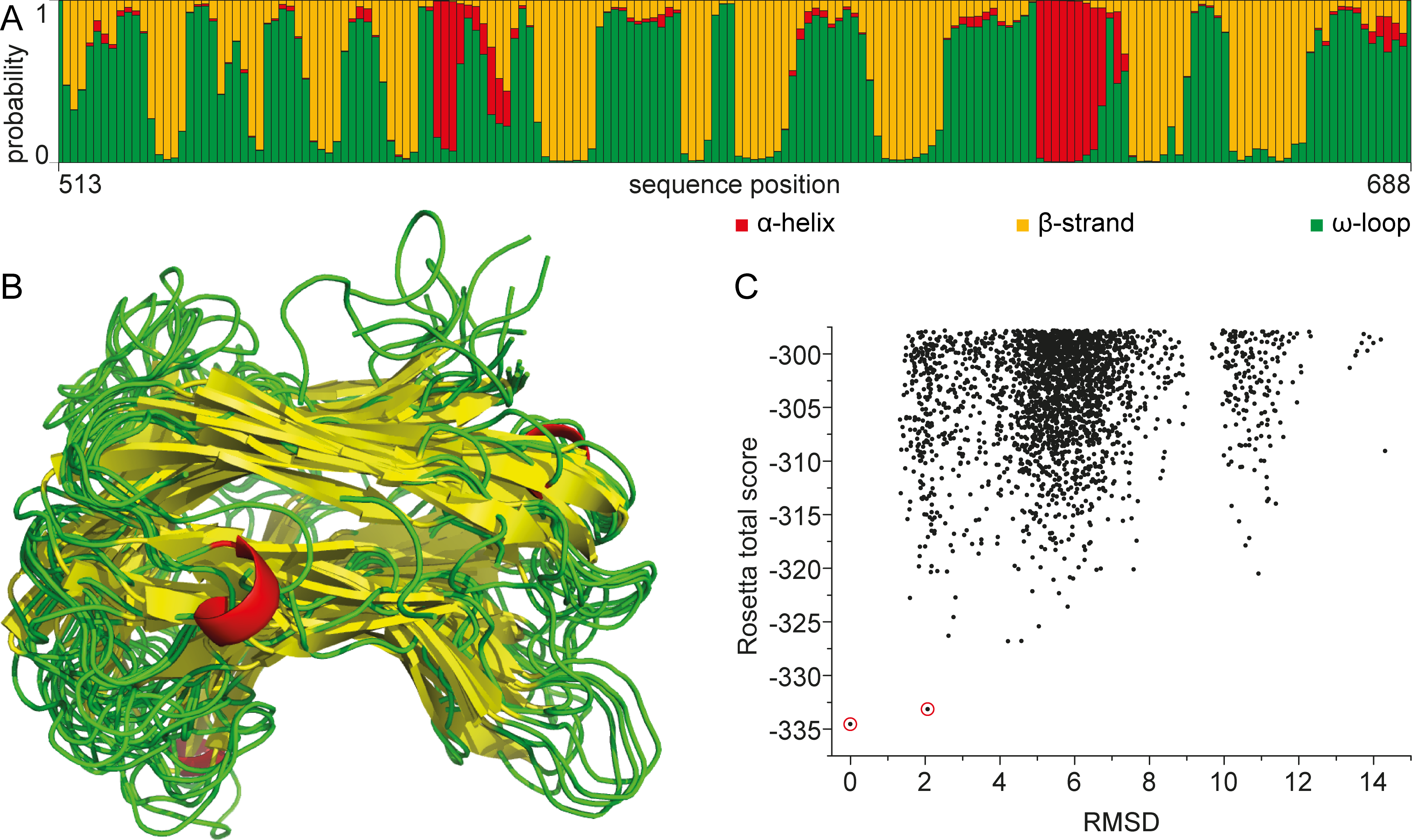

Supplement: Figure S8 — Comparative modeling of laminin γ1 L4. (A) PSIPRED secondary structure prediction for the L4 domain. A β-sheet-rich fold and one long α-helix are predicted. (B) MUSTANG alignment of 13 potential structural homologs of L4 identified by fold recognition using several threading servers. All template candidates exhibit a β-sandwich topology. The number of β-strands is in line with the predicted secondary structure of L4. Instead of an α-helix, all structures contain a long loop region. (C) Rosetta total score of the top 10% of all generated models plotted against their RMSD from the best-scoring structure. Only α-helices, β-sheets and short loops (≤5 residues) were included in RMSD calculations. The models are converging to a minimum in score and RMSD indicating that the best-scoring models are valid. The two best-scoring models shown in Figure 4 are marked with red circles. (TIF) [file pone.0112886.s008.tif]

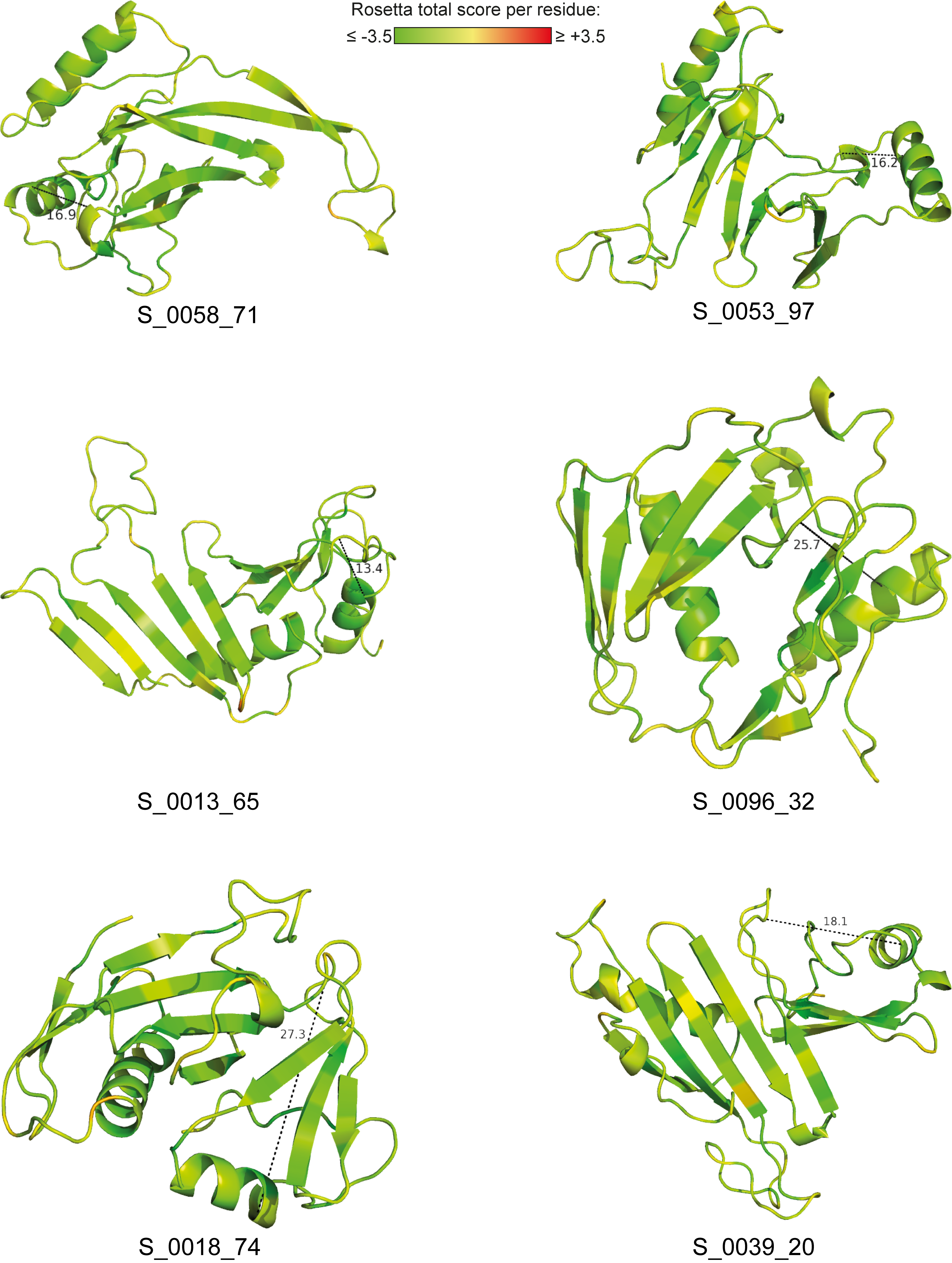

Supplement: Figure S9 — Best-scoring nidogen-1 NIDO models originating from common centroid models. The full-atom candidate structures of the NIDO domain were examined for common initial centroid models. Next to the centroid models underlying the structures depicted in Figure 5, we identified six centroid models that form the basis for more than three full-atom refined candidate structures. Shown are the best-scoring final candidate structures representing these initial centroid models. The Cα–Cα distances corresponding to the cross-link located within the models are given in Å. The residues are colored according to their Rosetta total score. Scores below zero (yellow-green color) indicate energetically favorable conformations. The identifiers of the underlying centroid models are given. (TIF) [file pone.0112886.s009.tif]
