# Supplementary material for: Analysis of Nidogen-1/Laminin γ1 Interaction by Cross-Linking, Mass Spectrometry, and Computational Modeling Reveals Multiple Binding Modes
Source: PLoS One. 2014 Nov 11;9(11):e112886. doi: 10.1371/journal.pone.0112886 (PMC4227867; doi:10.1371/journal.pone.0112886)
Supplement: Figure S2 — Clustal W2.1 sequence alignments for comparative modeling. Shown are pairwise sequence alignments of all nidogen-1 and laminin γ1 target sequences to template sequences with sequence identities ≥30%. The scheme for template sequences is termed ‘PDB-entry_domain-name_chain-identifier’. Annotations comply with the Clustal nomenclature with identical (*), conserved (:) and semi-conserved (.) residues being denoted. (DOC) [file pone.0112886.s002.doc]

Nidogen-1 EGF-like2

----------------------------------------------------------------------

EGF-like2 LQNPCYIGTHGCDSNAACRPGPGTQFTCECSIGFRGDGQTCY

1GL4_EGF1_A ---TCANNRHQCSVHAECR-DYATGFCCRCVANYTGNGRQCV

.* . * *. :* ** . .* * *.* .: *:*: *

----------------------------------------------------------------------

EGF-like2 ---LQNPCYIGTHGCDSNAACRPGPGTQFTC-ECSIGFRGDGQTCY

1YO8_EGF1_A DGCLSNPCFPG-------AQCSSFPDGSWSCGFCPVGFLGNGTHCE

*.***: * * * . *. .::* *.:** *:* *

----------------------------------------------------------------------

EGF-like2 LQNPCYIGTHGCDSNAACRPG---PGTQFTCECSIGFRGDGQTCY

1YO8_EGF2_A PENPCKDKTHNCHKHAECIYLGHFSDPMYKCECQTGYAGDGLICG

:*** **.*..:* * ... :.***. *: *** *

----------------------------------------------------------------------

EGF-like2 LQNPCYIGTHGCDSNAACRPG-PGTQFTCECSIGFRGDGQTCY

3S94_EGF1_A ATNPCGIDNGGCSHLCLMSPVKPFYQCACPTGVKLLENGKTCK

*** *.. **. . * * * :* .: : :*:**

----------------------------------------------------------------------

EGF-like2 LQNPCYIGTHGCDSNAACRPGPGTQFTCECSIGFR-GDGQTCY

3V64_EGF3_D GKNRCGDNNGGCTH--LCLPS-GQNYTCACPTGFRKINSHACA

:* * .. ** * *. * ::** *. *** :.::*

----------------------------------------------------------------------

----------------------------------------------------------------------

Nidogen-1 EGF-like3

----------------------------------------------------------------------

EGF-like3 DIDECSEQP---SRCGNHAVCNNLPGTFRCECVEGYHFS-DRGTCV

1SZB_EGF_A DIDECQVAPGEAPTCDHH--CHNHLGGFYCSCRAGYVLHRNKRTCS

*****. * . *.:* *:* * * *.* ** : :: **

----------------------------------------------------------------------

EGF-like3 -DIDECSEQPSRCGNHAVCNNLPGTFRCECVEGYHFSDRGTCV

1TOZ_EGF11_A QDVDECSLGANPCEHAGKCINTLGSFECQCLQGYTG---PRCE

*:**** .. * : . * * *:*.*:*::** *

----------------------------------------------------------------------

EGF-like3 DIDECSEQPSRCGNHAVCNNLPGTFRCECVEGYHFSDRGTCV

1TOZ_EGF12_A DVNECVSNP--CQNDATCLDQIGEFQCICMPGYEG---VHCE

*::** .:* * *.*.* : * *:* *: **. *

----------------------------------------------------------------------

EGF-like3 DIDECSEQPSRCGNHAVCNNLPGTFRCECVEGYHFSDRG-TCV

1UZJ_EGF27_A DIDECQELPGLCQ-GGKCINTFGSFQCRCPTGYYLNEDTRVCD

*****.* *. * . * * *:*:*.* **::.: .*

----------------------------------------------------------------------

EGF-like3 DIDECSEQ-PSRCGNHAVCNNLPGTFRCECVEGYHFSD---------RGTCV

2BO2_EGF2_A DINECATLSKVSCGKFSDCWNTEGSYDCVCSPGYEPVSGAKTFKNESENTCQ

**:**: **:.: * * *:: * * **. . ..**

----------------------------------------------------------------------

EGF-like3 DIDECSEQPSRCGNHAVCNNLPGTFRCECVEGYHFS-DRGTCV

2W86_EGF13_A DIDECESSPCING---VCKNSPGSFICECSSESTLDPTKTICI

*****...*. * **:* **:* *** . :. : *:

----------------------------------------------------------------------

EGF-like3 DIDECSEQPSRCGNHAVCNNLPGTFRCECVEGYHFSDRG-TCV

2W86_EGF14_A DIDECEVFPGVCKN-GLCVNTRGSFKCQCPSGMTLDATGRICL

*****. *. * * .:* * *:*:*:* .* :. * *:

----------------------------------------------------------------------

EGF-like3 DIDECSEQPSRCGNHAVCNNLPGTFRCECVEGYHFS-DRGTCV--

3S94_EGF2_A GSNPCAEENGGCS--HLCLYRPQGLRCACPIGFELISDMKTCIVP

. : *:*: . *. :* * :** * *:.: * **:

----------------------------------------------------------------------

----------------------------------------------------------------------

Nidogen-1 EGF-like4

----------------------------------------------------------------------

EGF-like4 -PINYCETGLHNCDIPQRAQCIYMGGSSYTCSCLPGFSGDGRACR

1TOZ_EGF11_A QDVDECSLGANPCEHAG--KCINTLG-SFECQCLQGYTG--PRCE

:: *. * : *: . :** * *: *.** *::* *.

----------------------------------------------------------------------

EGF-like4 PINYCETGLHNCDIPQRAQCIYMGGSS---YTCSCLPGFSGDGRACR

1YO8_EGF2_A PENPCKDKTHNCHK--HAECIYLGHFSDPMYKCECQTGYAGDGLICG

* * *: ***. :*:***:* * *.*.* .*::*** *

----------------------------------------------------------------------

----------------------------------------------------------------------

Nidogen-1 EGF-like5

----------------------------------------------------------------------

EGF-like5 DVDECQHSRCHP---DAFCYNTPGSFTCQCKPGYQGDGFR--CM

1SZB_EGF_A DIDECQVAPGEAPTCDHHCHNHLGGFYCSCRAGYVLHRNKRTCS

*:**** : .. * .*:* *.* *.*:.** . : *

----------------------------------------------------------------------

EGF-like5 -DVDECQHSR--CHPDAFCYNTPGSFTCQCKPGYQGDGFRCM

1TOZ_EGF11_A QDVDECSLGANPCEHAGKCINTLGSFECQCLQGYTG--PRCE

*****. . *. . * ** *** *** ** * **

----------------------------------------------------------------------

EGF-like5 DVDECQHSRCHPDAFCYNTPGSFTCQCKPGYQGDGFRCM

1TOZ_EGF12_A DVNECVSNPCQNDATCLDQIGEFQCICMPGYEG--VHCE

**:** . *: ** * : *.* * * ***:* .:*

----------------------------------------------------------------------

EGF-like5 -DVDECQHSRCHPDAFCYNTPGSFTCQCKPGYQGDGFRCM--

1UZJ_EGF26_A TDVNECLDPTTCISGNCVNTPGSYICDCPPDFELNPTRVGCV

**:** .. .. * *****: *:* *.:: : *

----------------------------------------------------------------------

EGF-like5 DVDECQHSR--CHPDAFCYNTPGSFTCQCKPGYQG--DGFRCM

1UZJ_EGF27_A DIDECQELPGLCQ-GGKCINTFGSFQCRCPTGYYLNEDTRVCD

*:****. *: .. * ** *** *:* .** * *

----------------------------------------------------------------------

EGF-like5 DVDECQHSRCHPDAFCYNTP-GSFTC-QCKPGYQGDGFRCM

1YO8_EGF1_A --DGCLSNPCFPGAQCSSFPDGSWSCGFCPVGFLGNGTHCE

* * . *.*.* * . * **::* * *: *:* :*

----------------------------------------------------------------------

EGF-like5 DVDECQHS---RCHPDAFCYNTPGSFTCQCKPGYQ----GDGFR------CM

2BO2_EGF2_A DINECATLSKVSCGKFSDCWNTEGSYDCVCSPGYEPVSGAKTFKNESENTCQ

*::** * : *:** **: * *.***: .. *: *

----------------------------------------------------------------------

EGF-like5 DVDECQHSRCHPDAFCYNTPGSFTCQCKPGYQGDGFR--CM

2W86_EGF13_A DIDECESSPCIN-GVCKNSPGSFICECSSESTLDPTKTICI

*:***: * * ..* *:**** *:*.. * : *:

----------------------------------------------------------------------

EGF-like5 DVDECQHSR-CHPDAFCYNTPGSFTCQCKPGYQGD--GFRCM

2W86_EGF14_A DIDECEVFPGVCKNGLCVNTRGSFKCQCPSGMTLDATGRICL

*:***: :.:* ** ***.*** .* * * *:

----------------------------------------------------------------------

EGF-like5 DVDECQHS-RCHPDAFCYNTPGSFTCQCKPGYQGD--GFRCM

3P5B_EGF2_L DIDECQDPDTCS--QLCVNLEGGYKCQCEEGFQLDPHTKACK

*:****.. * :* * *.:.***: *:* * *

----------------------------------------------------------------------

----------------------------------------------------------------------

Nidogen-1 EGF-like6

----------------------------------------------------------------------

EGF-like6 -GHNYCSVNNGGCTHL--CLPTPGSRTCRCPDNTLGVDCI

1TOZ_EGF11_A QDVDECSLGANPCEHAGKCINTLGSFECQCLQGYTGPRCE

. : **:. . * * *: * ** *:* :. * *

----------------------------------------------------------------------

EGF-like6 GHNYCSVNNGGCTH-LCLPTPGSRTCRCPD----NTLGVDCI

2W86_EGF14_A DIDECEVFPGVCKNGLCVNTRGSFKCQCPSGMTLDATGRICL

. : *.* * *.: **: * ** .*:**. :: * *:

----------------------------------------------------------------------

EGF-like6 GHNYCSVNNGGCTHLCLPTPGSRTCRCPDNTLGVDCI---

3P5B_EGF1_L --NECLDNNGGCSHVCNDLKIGYECLCPDGFQLVAQRRCE

* * *****:*:* . * ***. *

----------------------------------------------------------------------

EGF-like6 GHNYCSVNNGGCTHLCLPTPGS--RTCRCPDNT----LGVDCI

3S94_EGF1_A ATNPCGIDNGGCSHLCLMSPVKPFYQCACPTGVKLLENGKTCK

. * *.::****:**** :* . * ** .. * *

----------------------------------------------------------------------

EGF-like6 GHNYCSVNNGGCTHLCLPTPGSRTCRCPDNTLGVDCI------

3S94_EGF2_A GSNPCAEENGGCSHLCLYRPQGLRCACPIGFELISDMKTCIVP

* * *: :****:**** * . * ** . :. :

----------------------------------------------------------------------

EGF-like6 GHNYCSVNNGGCTHLCLPTPGSRTCRCPDNTLGVDCI---

3V64_EGF3_D GKNRCGDNNGGCTHLCLPSGQNYTCACPTGFRKINSHACA

*:* *. ***********: . ** ** . ::.

----------------------------------------------------------------------

----------------------------------------------------------------------

Nidogen-1 TY1

----------------------------------------------------------------------

TY1 KTRCQLEREHILGAAGGADAQRPTLQGMFVPQCDEYGHYVPTQCHHSTGYCWCVDR

1icf_ty1 LTKCQEEVSHIPAVHP----------GSFRPKCDENGNYLPLQCYGSIGYCWCVFP

*:** * .** .. * * *:*** *:*:* **: * ******

TY1 DGRELEGSRTPPGMRPPC

1icf_ty1 NGTEVPNTRSRGHHNC--

:* *: .:*: .

----------------------------------------------------------------------

TY1 KTRCQLEREHILG-AAGGADAQRPTLQGMFVPQCDEYGHYVPTQCHHST----GYC

2dsr_ty1 -GSCQSELHRALERLAASQSRTHEDLYIIPIPNCDRNGNFHPKQCHPALDGQRGKC

** * .: * *.. . : * : :*:**. *:: *.*** : * *

TY1 WCVDRD-GRELEGSRTPPGMRPPC

2dsr_ty1 WCVDRKTGVKLPGGLEPKGE----

*****. * :* *. * *

----------------------------------------------------------------------

Laminin γ1 LEa3

----------------------------------------------------------------------

LEa3 CHCSPVGSLSTQCDS---YGRCSCKPGVMGDKCDRCQPGFHSLTEAG----

1klo_LEb4_A CACNPYGTVQQQSSCNPVTGQCQCLPHVSGRDCGTCDPGYYNLQSGQGCER

* *.* *::. *... *:*.* * * * .*. *:**::.* ..

----------------------------------------------------------------------

LEa3 CHCSPVGSLSTQCDS---YGRCSCKPGVMGDKCDRCQPGFHSLTEAG----

1npe_LEb4_B CACNPYGTVQQQSSCNPVTGQCQCLPHVSGRDCGTCDPGYYNLQSGQGCER

* *.* *::. *... *:*.* * * * .*. *:**::.* ..

----------------------------------------------------------------------

LEa3 CHCSPVGSLS-TQCDSY---------GRCSCKPGVMGDKCDRCQPGFHSLTEAG--

4aqs_LEa3_A CTCDPAGSENGGICDGYTDFSVGLIAGQCRCKLHVEGERCDVCKEGFYDLSAEDPY

* *.*.** . **.* *:* ** * *::** *: **:.*: .

LEa3 ----

4aqs_LEa3_A GCKS

----------------------------------------------------------------------

----------------------------------------------------------------------

Laminin γ1 LEa4

----------------------------------------------------------------------LEa4 CSCDLRG---STDECNVETGRCV-CKDNVEGFNCERCKPGFFN--LESSNPKGCTP

1klo_LEb3_A CQCNDNIDPNAVGNCNRLTGECLKCIYNTAGFYCDRCKEGFFGNPLAPNPADKCKA

*.*: . :..:** **.*: * *. ** *:*** ***. * .. .. *..

----------------------------------------------------------------------

LEa4 CSCDLRGST---DECNVETGRCVCKDNVEGFNCERCKPGFFNLESSNPKGCTP

1klo_LEb4_A CACNPYGTVQQQSSCNPVTGQCQCLPHVSGRDCGTCDPGYYNLQSG--QGCER

*:*: *:. ..** **:* * :*.* :* *.**::**:*. :**

----------------------------------------------------------------------

LEa4 CSCDLRG---STDECNVETGRCV-CKDNVEGFNCERCKPGFFN--LESSNPKGCTP

1npe_LEb3_B CQCNDNIDPNAVGNCNRLTGECLKCIYNTAGFYCDRCKEGFFGNPLAPNPADKCKA

*.*: . :..:** **.*: * *. ** *:*** ***. * .. .. *..

----------------------------------------------------------------------

LEa4 CSCDLRGST---DECNVETGRCVCKDNVEGFNCERCKPGFFNLESSNPKGCTP

1npe_LEb4_B CACNPYGTVQQQSSCNPVTGQCQCLPHVSGRDCGTCDPGYYNLQSG--QGCER

*:*: *:. ..** **:* * :*.* :* *.**::**:*. :**

----------------------------------------------------------------------

LEa4 CSCDLRGSTD----------ECNVETGRCVCKDNVEGFNCERCKPGFFNLESSNPK

4aqs_LEa3_A CTCDPAGSENGGICDGYTDFSVGLIAGQCRCKLHVEGERCDVCKEGFYDLSAEDPY

*:** ** : . .: :*:* ** :*** .*: ** **::*.:.:*

LEa4 GCTP

4aqs_LEa3_A GCKS

**..

----------------------------------------------------------------------

LEa4 CSCDLRGSTDECNVETGR--------CVCKDNVEGFNCERCKPGFFNLESSNPK

4aqt_LEa2_A CDCNGRSQECYFDPELYRSTGHGGHCTNCRDNTDGAKCERCRENFFRLG--NTE

*.*: *.. : * * *:**.:* :****: .**.* *.:

LEa4 GCTP

4aqt_LEa2_A ACSP

.*:*

----------------------------------------------------------------------

----------------------------------------------------------------------

Laminin γ1 LEa5.2

----------------------------------------------------------------------

LEa5.2 -------------------CT-CPVGYGGQFCETCLPGYR-RETPSLGPYSPCVL

1klo_LEb2_A CPCPGGSSCAIVPKTKEVVCTHCPTGTAGKRCELCDDGYFGDPLGSNGPVRLCRP

** **.* .*: ** * ** * ** *

----------------------------------------------------------------------

LEa5.2 ---------------------CTCPVGYGGQFCETCLPGYRRETPSLGPYSPCVL

1klo_LEb4_A CACNPYGTVQQQSSCNPVTGQCQCLPHVSGRDCGTCDPGYYNLQSGQG----CER

* * .*: * ** *** . .. * *

----------------------------------------------------------------------

LEa5.2 -------------------CT-CPVGYGGQFCETCLPGYR-RETPSLGPYSPCVL

1npe_LEb2_B CPCPGGSSCAIVPKTKEVVCTHCPTGTAGKRCELCDDGYFGDPLGSNGPVRLCRP

** **.* .*: ** * ** * ** *

----------------------------------------------------------------------

LEa5.2 ---------------------CTCPVGYGGQFCETCLPGYRRETPSLGPYSPCVL

1npe_LEb4_B CACNPYGTVQQQSSCNPVTGQCQCLPHVSGRDCGTCDPGYYNLQSGQG----CER

* * .*: * ** *** . .. * *

----------------------------------------------------------------------

----------------------------------------------------------------------

Laminin γ1 LEb1

----------------------------------------------------------------------

LEb1 CTCNGHS----ETCDPETGVCDCRDNTAGPHCEKCSDGYYGD--STLGTSSDCQP

1klo_LEb2_A CPCPGGSSCAIVPKTKEVVCTHCPTGTAGKRCELCDDGYFGDPLGSNGPVRLCRP

*.* * * . *. .* .*** :** *.***:** .: *. *:*

----------------------------------------------------------------------

LEb1 CTCNGHSET-----CDPETGVCD-CRDNTAGPHCEKCSDGYYGDSTLG-TSSDCQP

1klo_LEb3_A CQCNDNIDPNAVGNCNRLTGECLKCIYNTAGFYCDRCKEGFFGNPLAPNPADKCKA

* **.: :. *: ** * * **** :*::*.:*::*:. .:..*:.

----------------------------------------------------------------------

LEb1 CTCNGHS----ETCDPETGVCDCRDNTAGPHCEKCSDGYYGD--STLGTSSDCQP

1npe_LEb2_B CPCPGGSSCAIVPKTKEVVCTHCPTGTAGKRCELCDDGYFGDPLGSNGPVRLCRP

*.* * * . *. .* .*** :** *.***:** .: *. *:*

----------------------------------------------------------------------

LEb1 CTCNGHSET-----CDPETGVCD-CRDNTAGPHCEKCSDGYYGDSTLG-TSSDCQP

1npe_LEb3_B CQCNDNIDPNAVGNCNRLTGECLKCIYNTAGFYCDRCKEGFFGNPLAPNPADKCKA

* **.: :. *: ** * * **** :*::*.:*::*:. .:..*:.

----------------------------------------------------------------------

LEb1 CTCNGHSETCD------PETGVCDCRDNTAGPHCEKCSDGY----YGDSTLGTSS

2y38_LEa1_A CVCHGHADVCDAKDPLDPFRLQCACQHNTCGGSCDRCCPGFNQQPWKPATTDSAN

*.*:**::.** * * *:.**.* *::*. *: : :* .::.

LEb1 DCQP

2y38_LEa1_A ECQS

:**.

----------------------------------------------------------------------

LEb1 CTCNGHSETCDPETG-----VCDCRDNTAGPHCEKCSDGYYG----DSTLGTSSD

4aqs_LEa1_A CFCYGHASECAPVVEGMVHGHCMCRHNTKGLNCELCMDFYHDLPWRPAEGRNSNA

* * **:. * * . * **.** * :** * * *:. : .*.

LEb1 CQP

4aqs_LEa1_A CKK

*:

----------------------------------------------------------------------

LEb1 CTCNGHSETCDP-----------ETGVCD-CRDNTAGPHCEKCSDGYYG--DSTLG

4aqs_LEa2_A CNCNEHSSSCHFDMAVFLATGNVSGGVCDNCQHNTMGRNCEQCKPFYFQHPERDIR

*.** **.:*. . **** *:.** * :**:*. *: : :

LEb1 TSSDCQP

4aqs_LEa2_A DPNLCEP

.. *:*

----------------------------------------------------------------------

LEb1 CTCNGHSETCDP---ETGVCDCRDNTAGPHCEKCS----DGYYGDSTLGTSSDCQP

4aqt_LEa1_A CKCNGHASECVKNEFDKLMCNCKHNTYGVDCEKCLPFFNDRPWRRATAESASECLP

*.****:. * :. :*:*:.** * .**** * : :* ::*:* *

----------------------------------------------------------------------

LEb1 CTCNGHSETC--DPETG--------VCDCRDNTAGPHCEKCSDGYYGDSTLGTSSD

4aqt_LEa2_A CDCNGRSQECYFDPELYRSTGHGGHCTNCRDNTDGAKCERCRENFFR---LGNTEA

* ***:*: * *** :***** *.:**:* :.:: **.:.

LEb1 CQP

4aqt_LEa2_A CSP

*.*

----------------------------------------------------------------------

----------------------------------------------------------------------

Laminin γ1 LEb5

----------------------------------------------------------------------

LEb5 CDCHALGSTN--------GQCDIRTGQCE-CQPGITGQHCERCETNHFGFG-----

4aqs_LEa2_A CNCNEHSSSCHFDMAVFLATGNVSGGVCDNCQHNTMGRNCEQCKPFYFQHPERDIR

*:*: .*: . :: * *: ** . *::**:*:. :* .

LEb5 -PEGCKP

4aqs_LEa2_A DPNLCEP

*: *:*

----------------------------------------------------------------------

LEb5 CDCHALGSTNG---------QCDIRTGQCECQPGITGQHCERCETNHFGFGPE---

4aqs_LEa3_A CTCDPAGSENGGICDGYTDFSVGLIAGQCRCKLHVEGERCDVCKEGFYDLSAEDPY

* *.. ** ** . .: :***.*: : *::*: *: ..:.:..*

LEb5 GCKP

4aqs_LEa3_A GCKS

***.

----------------------------------------------------------------------

LEb5 CDCHALGS---TNGQCDIRTGQ----CECQPGITGQHCERCETNHFGFG-PEGCKP

4aqt_LEa2_A CDCNGRSQECYFDPELYRSTGHGGHCTNCRDNTDGAKCERCRENFFRLGNTEACSP

***:. .. : : **: :*: . * :****. *.* :* .*.*.*

----------------------------------------------------------------------

Figure S 2. Clustal W2.1 sequence alignments for comparative modeling. Shown are pairwise sequence alignments of all nidogen-1 and laminin γ1 target sequences to template sequences with a sequence identity ≥ 30%. The naming scheme for template sequences is ‘PDB entry_domain name_chain identifier’. Annotations comply with the Clustal nomenclature with identical (*), conserved (:) and semi-conserved (.) residues being denoted.
