# Supplementary material for: Analysis of Nidogen-1/Laminin γ1 Interaction by Cross-Linking, Mass Spectrometry, and Computational Modeling Reveals Multiple Binding Modes
Source: PLoS One. 2014 Nov 11;9(11):e112886. doi: 10.1371/journal.pone.0112886 (PMC4227867; doi:10.1371/journal.pone.0112886)
Supplement: Figure S3 — Manual sequence alignments for modeling of the laminin γ1 L4 domain. Shown are pairwise sequence alignments to all template sequences. Alignments are manually optimized to obtain maximum overlap of secondary structure elements. The scheme for template sequences is termed ‘PDB-entry_chain-identifier’. (DOC) [file pone.0112886.s003.doc]

----------------------------------------------------------------------

L4 ---------DISSTFQIDEDGWRVEQRDGSEASLEWSSDRQDIAVISDSYF-----

1CX1_A ASLDSEVELLPHTSFAESLGPWSLYGTSEP------VFADGRMCVDLPGGQGNPWD

L4 PRYFIAPVKFLGNQVLSYGQNLSFSFRVDRRDTRLSAEDLVLEGAG----------

1CX1_A AGLVYNGVPVGE----GESYVLSFTASATP-------DMPVRVLVGEGGGAYRTAF

L4 LRVSVPLIAQGNSYPSETTVKYIFRLHEATDYPWRPALSPFEFQKLLNNLTSIKIR

1CX1_A EQGSAPLT-------GEPATREYAFTSNLTFPPDGDA------------PGQVAFH

L4 GTYSERTAGYLDDVTLQSARPGPGVPATWVES

1CX1_A LGKAGAYEFCISQVSLTTSAT-----------

----------------------------------------------------------------------

L4 --DISSTFQIDEDGWRVEQRDGSEASLEWSSDR-----------QDIAVISDSYFP 1D7B_A PSASQFTDPTTGFQFTGITDPVHDVTYGFVFPPLATSGAQSTEFIGEVVAPIASKW

L4 RYFIAPVKFLGNQVLSYGQNLSFSFRVDRRDTRLSAEDLVLE------------GA 1D7B_A IGIALGGAMNNDL------LLVAWANGN-------QIVSSTRWATGYVQPTAYTGT

L4 GLRVSVPL IAQGNSYPSETTVKYIFRLHEATDYPWRPALSPFEFQKLLNNLTSIK 1D7B_A ATLTTLPE TTI-----NSTHWKWVFRCQGCTEWNNGGGID---------VTSQGV

L4 IRGTYSERTA-------------GYLDDVTLQSARPGPGVPATWVES

1D7B_A LAWAFSNVAVDDPSDPQSTFSEHTDFGFFGIDYSTAHSANYQNYLN-

----------------------------------------------------------------------

L4 ----DISSTFQIDE-DGWRVEQRDGSEASLEWSSDR-----QDIAVISDSY---FP 1DYO_A PDAGYYYHDTFEGSVGQWTAR-----GPAEVLLSGRTAYKGSESLLVRNRTAAWNG

L4 RYFIAPVKFLGNQVLSYGQNLSFSFRVDRRDTRLSAEDLVLEGAG----------L 1DYO_A AQRALNPRTFVP------GNTYCFSVVASFIEGASSTTFCMKLQYVDGSGTQRYDT

L4 RVSVPLIAQGNSYPSETTVKYIFRLHEATDYPWRPALSPFEFQKLLNNLTSIKIRG 1DYO_A IDMKTVGPNQW------VHLYNPQYRIPS-----------------DATDMYVYVE

L4 TYSERTAGYLDDVTLQSARPGPGVPATWVES

1DYO_A TADDTINFYIDEAIGAVAGTVI---------

----------------------------------------------------------------------

L4 DISSTFQIDEDGWRVEQRDGSEASLEWSSDRQDIAVISDS----YFPRYFIAPVKF 1GU3_A ----TFDDGPEGWVAYGTDG-----PLDTSTGALCVAVPAGSAQYGVGVVL-----

L4 LGNQVLSYGQNLSFSFRVDRRDTRLSAEDLVLEGA------GLRVSVPLIAQGNSY 1GU3_A NGVAIEEGTTYTLRYTATASTDV----TVRALVGQNGAPYGTVLDTSPALTS----

L4 PSETTVKYIFRLHEATDYPWRPALSPFEFQKLLNNLTSIKIRGTYSERTA---GYL 1GU3_A --EPRQVTETFTASATYPATPAADDP----------EGQIAFQLGGFSADAWTLCL

L4 DDVTLQSARPGPGVPATWVES

1GU3_A DDVALDSE-------------

----------------------------------------------------------------------

L4 ----------DISSTFQIDEDGWRVEQRDGSE----ASLEWSSDRQDIAVISD--- 1GUI_A SINNGTFDEPIVNDQANNPDEWFIWQAGDYGISGARVSDYGVRDGYAYITIADPGT

L4 -SYFPRYFIAPVKFLGNQVLSYGQNLSFSFRVDRRDTRLSAEDLVLEGA-----GL 1GUI_A DTWHIQFNQWIG-------LYRGKTYTISFKAKAD---TPRPINVKILQNHDPWTN

L4 RVSVPLIAQGNSYPSETTVKYIFRLHEATDYPWRPALSPFEFQKLLNNLTSIKIRG 1GUI_A YFAQTVNLTA-----DWQTFTFTYTHPDDADE-----------------VVQISFE

L4 TYSERTAGYLDDVTLQSARPGPGVPATWVES

1GUI_A LGEGTATTIYFDDVTVSPQ------------

----------------------------------------------------------------------

L4 ---------------DISSTFQIDEDGWRVEQRDGSEASL--EWSSDRQD------ 1K42_A MLVANINGGFESTPAGVVTDLAEGVEGWDLNVGSSVTNPPVFEVLETSDAPEGNKV

L4 IAVISDSY-----FPRYFIAPVKFLGNQVLSYGQNLSFSFRVDRRDTRLSAEDLVL 1K42_A LAVTVNGVGNNPWDIEATAFPVNVRPG----VTYTYTIWARAEQD----GAVVSFT

L4 E-----GAGLRVSVPLIAQGNSYPSETTVKYIFRLHEATDYPWRPALSPFEFQKLL 1K42_A VGNQSFQEYGRLHEQQIT------TEWQPFTFEFTVSDQE----------------

L4 NNLTSIKIRGTYSERTA-GYLDDVTLQSARPGPGVPATWVES

1K42_A -TVIRAPIHFGYAANVGNTIYIDGLAIASQP-----------

----------------------------------------------------------------------

L4 -DISSTFQIDEDGWRVEQRDGSEASLEWSSD------RQDIAVISDSYF-PRYFIA 1WKY_A PTTLYDFEESTQGWTGSSLS---RGPWTVTEWSSKGNHSLKADIQMSSNSQHYLHV

L4 PVKFLGNQVLSYGQNLSFSFRVDRRDTRLSAEDLVLE—GAGLRVSVPLIAQGNSYP 1WKY_A IQNRSLQQN----SRIQATVKHA-----GMTARLYVKTGHGYTWYSGSFVPING-S

L4 SETTVKYIFRLHEATDYPWRPALSPFEFQKLLNNLTSIKIRGTYSERTA---GYLD 1WKY_A S---GTTLSLDLSNVQNLSQ---------------VREIGVQFQSESNSSGQTSIY

L4 DVTLQSARPGPGVPATWVES

1WKY_A IDNVIVE--------------

----------------------------------------------------------------------

L4 --DISSTFQIDEDGWRVEQR---------DGSEASLEWSSDRQDIAVISD---SYF 1WMX_A LLDVQIFKDSPVVGWSGSGMGELETIGDTLPVDTTVTYNGLPTLRLNVQTTVQSGW

L4 PRYFIAPVKFLGNQVLSYGQ--NLSFSFRVDRRDTRLSAEDLVLEG------AGLR 1WMX_A WISLLTLRGWNTHDLSQYVENGYLEFDIKG--KEGGEDFVIGFRDKVYERVYGLEI

L4 VSVPLIAQGNSYPSETTVKYIFRLHEATDYPWRPALSPFEFQKLLNNLTSIKIRGT 1WMX_A DVTTVISNYVTV---TTDWQHVKIPLRDLMKINNGF--------DPSSVTCLVFSK

L4 YSERTAGYLDDVTLQSARPGPGVPATWVES

1WMX_A RYADP-FTVWFSDIKITSE-----------

----------------------------------------------------------------------

L4 DISSTFQIDEDG-------WRVEQRDGSEASLEWSSDRQ----DIAVISDSYFPRY 2ZEW_A -SHMVNMVSNPGFEDGLDSWQDWQQD-----MSAVPEAAHNGALGLKIGGGKAAGG

L4 FIAPVKFLGNQVLSYGQNLSFSFRVDRRDTRLSAEDLVLEG-----AGLRVSVPLI 2ZEW_A GQDIPLK-----PNTTYILGAWAKFDSKPAGT-FDVVVQYHLKDANNTYVQHILNF

L4 AQGNSYPSETTVKYIFRLHEATDYPWRPALSPFEFQKLLNNLTSIKIRGTYSERTA 2ZEW_A N-----ETDWTYKQLLFTTPDVFGST-----------------PQLALWKGDTSKA

L4 GYLDDVTLQSARPGPGVPATWVES

2ZEW_A NLYVDDVYLVEV------------

----------------------------------------------------------------------

L4 DISSTFQIDEDG-------WRVEQRDGSEASLEWSSDRQ----DIAVISDSYFPRY 2ZEW_B --------SNPGFEDGLDSWQDWQQD-----MSAVPEAAHNGALGLKIGGGKAAGG

L4 FIAPVKFLGNQVLSYGQNLSFSFRVDRRDTRLSAEDLVLEG-----AGLRVSVPLI 2ZEW_B GQDIPLK-----PNTTYILGAWAKFDSKPAGT-FDVVVQYHLKDANNTYVQHILNF

L4 AQGNSYPSETTVKYIFRLHEATDYPWRPALSPFEFQKLLNNLTSIKIRGTYSERTA 2ZEW_B N-----ETDWTYKQLLFTTPDVFGST-----------------PQLALWKGDTSKA

L4 GYLDDVTLQSARPGPGVPATWVES

2ZEW_B NLYVDDVYLVEV------------

----------------------------------------------------------------------

L4 DISSTFQIDEDGWRVEQRDGSEASLEWSSDRQD----IAVISDSYFPRYFIAPVKF 2ZEZ_A IVNGTAENGMDGW-----PDWGYPVSAVPEAAYGGTKGFKLSGGKQAGMGQKV---

L4 LGNQVLSYGQNLSFSFRVDRRDTRLSAEDLVLEGAG-----LRVSVPLIAQGNSYP 2ZEZ_A --ALKPNTTYILGAWGKFTAKPGTY-CDVIVQYHLKDANNTYVQNILRF-----TE

L4 SETTVKYIFRLHEATDYPWRPALSPFEFQKLLNNLTSIKIRGTYSERTAGYLDDVT 2ZEZ_A TDWTYKQVVFTTPDAFG----------------SDPEFVLWKDDASNADFYADNIT

L4 LQSARPGPGVPATWVES

2ZEZ_A LVE--------------

----------------------------------------------------------------------

L4 --DISSTFQIDED--GWRVEQ-------------RDGSEASLEWSSD---RQDIAV 3F95_A SDSHPLFVRSLAKNMTWQLADTSTQKVLASGASATSGDKQSLLM-VNLSYQEDGRG

L4 ISDSYFPRYFI------APVKFLGNQVLSYGQNLSFSFRVDRRDTRLSAEDLVLEG 3F95_A F-WRAQAALSLYLEPTPLDSKFS----TGYLELKMRIDKAP-------EQGANLQV

L4 -----AGLRVSVPLIAQGNSYPSETTVKYIFRLHEATDYPWRPALSPFEFQKLLNN 3F95_A MCSESNCLRDIDFSSFS--LMADKSWHTLAIPLHC---------------------

L4 LTSIKIRGTYSERTAGYLDDVTLQSARPGPGVPATWVES

3F95_A -QPITDALRITSQNL-LAIADVALTIKPSDDSI-LTCAK

----------------------------------------------------------------------

L4 DISSTFQIDEDGWRVEQRDGSEASLEWSSDRQ----DIAVISDSYFPRYFIAPVKF 3OEA_A VSNPGFEDGLDSWQDWQQD-----MSAVPEAAHNGALGLKIGGGKAAGGGQDI---

L4 LGNQVLSYGQNLSFSFRVDRRDTRLSAEDLVLEG-----AGLRVSVPLIAQGNSYP 3OEA_A --PLKPNTTYILGAWAKFDSKP-AGTFDVVVQYHLKDANNTYVQHILNFNE-----

L4 SETTVKYIFRLHEATDYPWRPALSPFEFQKLLNNLTSIKIRGTYSERTA-GYLDDV 3OEA_A TDWTYKQLLFTTPDVFGST-----------------PELALWKGDTSKANLYVDDV

L4 TLQSARPGPGVPATWVES

3OEA_A YLVEV-------------

----------------------------------------------------------------------

L4 --DISSTFQIDEDGWRVEQRDGSEASLEWSSDRQDIAVISD---------SYFPRY 3ZXJ_A TDLDEFRGTRFSEHWEWNHNPDTSK-FTLLGGNEGGLILRTATVTGDLFAARNTLT

L4 FIAPVKFLGNQVLSYGQNLSFSFRVDRRDTRLSAED-------------LVLEGAG 3ZXJ_A RRIAGP-----------KASGIFRLDVRGMRDGDRAGAVLFRDRAAYIGVWKQGNE

L4 LRVSVPLIAQGNSYPSET--TVKYIFRLHEATDYPWRPALSPFEFQKLLNNLTSIK 3ZXJ_A ARIVMVDDLRLNEDGWRTASTGRVAANGPVIDTNAQ--------------QDIWLR

L4 IRGTYSERTA------GYLDDVTLQSARPGPGVPATWVES

3ZXJ_A IDADITPAFG----TNTERTTTFYYSIDGGRTYTRLGP—

----------------------------------------------------------------------

Figure S 3. Manual sequence alignments for modeling of the laminin γ1 L4 domain. Shown are pairwise sequence alignments to all template sequences. Alignments are manually optimized to obtain maximum overlap of secondary structure elements. The naming scheme for template sequences is ‘PDB entry_chain identifier’.
