# Supplementary material for: Analysis of Nidogen-1/Laminin γ1 Interaction by Cross-Linking, Mass Spectrometry, and Computational Modeling Reveals Multiple Binding Modes
Source: PLoS One. 2014 Nov 11;9(11):e112886. doi: 10.1371/journal.pone.0112886 (PMC4227867; doi:10.1371/journal.pone.0112886)
Supplement: Figure S4 — Jalview sequence alignment of nidogen-1 NIDO domains from different organisms. All NIDO domains share sequence identities >75% but exhibit short sequence stretches that are diverse. (DOC) [file pone.0112886.s004.doc]

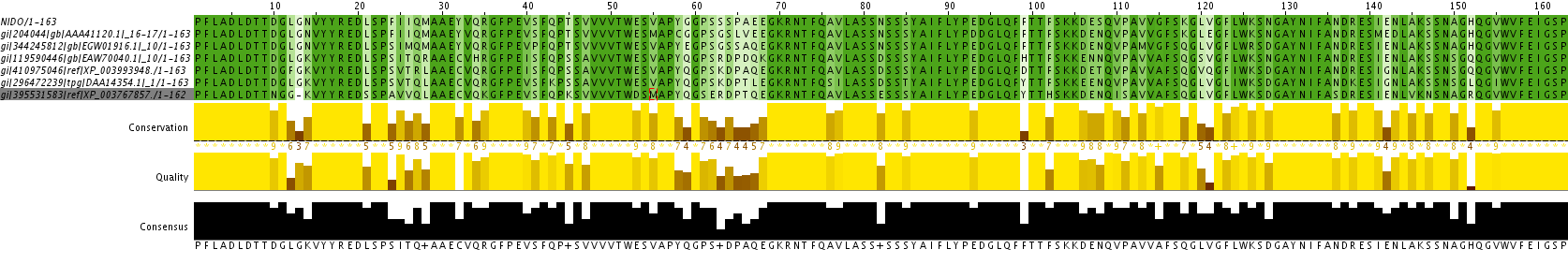


**Figure S 4. Jalview sequence alignment of nidogen-1 NIDO domains from different organisms.** All NIDO domains share a sequence identity >75% but exhibit short sequence stretches that are diverse.
