# Supplementary material for: Analysis of Nidogen-1/Laminin γ1 Interaction by Cross-Linking, Mass Spectrometry, and Computational Modeling Reveals Multiple Binding Modes
Source: PLoS One. 2014 Nov 11;9(11):e112886. doi: 10.1371/journal.pone.0112886 (PMC4227867; doi:10.1371/journal.pone.0112886)
Supplement: Table S1 — Results of Rosetta clustering of comparative nidogen-1 and laminin γ1 domain models. The best 10% of all generated models were clustered. The ideal clustering radius was automatically determined by the Rosetta algorithm. Shown are clusters with a size >1. (DOC) [file pone.0112886.s010.doc]

Table S 1. Results of Rosetta clustering of comparative nidogen-1 and laminin γ1 domain models. The best 10% of all generated models were clustered. The ideal clustering radius was automatically determined by the Rosetta algorithm. Shown are clusters with a size >1.

| **Cluster** | **Rosetta**  **total score** | **Size** |
| --- | --- | --- |
| **nidogen-1 EGF-like2** | | |
| 1 | -65.032 | 3343 |
| 2 | -64.531 | 1885 |
| 3 | -64.315 | 976 |
| 4 | -63.822 | 1196 |
| 5 | -61.734 | 459 |
| 6 | -60.959 | 96 |
| 7 | -60.666 | 54 |
| 8 | -58.555 | 298 |
| 9 | -57.3 | 1071 |
| 10 | -56.562 | 87 |
| 11 | -55.502 | 52 |
| 12 | -55.171 | 121 |
| 13 | -54.498 | 45 |
| 14 | -54.463 | 3 |
| 15 | -54.28 | 21 |
| 16 | -54.202 | 127 |
| 17 | -51.877 | 18 |
| 18 | -51.739 | 11 |
| 19 | -51.56 | 109 |
| 20 | -47.579 | 28 |
| 21 | -42.007 | 20 |
| 22 | -30.339 | 2 |
| 23 | -26.876 | 2 |
| 24 | -26.832 | 2 |
| 25 | -26.714 | 2 |
| 26 | -26.288 | 2 |
| 27 | -23.255 | 2 |
| 28 | -21.104 | 5 |
| 29 | -18.568 | 2 |
| 30 | -17.24 | 2 |
| 31 | -1.654 | 4 |
| **nidogen-1 EGF-like3** | | |
| 1 | -58.568 | 2154 |
| 2 | -58.525 | 232 |
| 3 | -58.469 | 411 |
| 4 | -58.016 | 59 |
| 5 | -57.854 | 273 |
| 6 | -57.641 | 564 |
| **Cluster** | **Rosetta**  **total score** | **Size** |
| 7 | -57.376 | 109 |
| 8 | -56.995 | 26 |
| 9 | -56.859 | 217 |
| 10 | -56.83 | 6 |
| 11 | -56.47 | 104 |
| 12 | -56.076 | 117 |
| 13 | -55.956 | 160 |
| 14 | -55.925 | 82 |
| 15 | -55.578 | 61 |
| 16 | -55.447 | 11 |
| 17 | -55.311 | 162 |
| 18 | -55.164 | 53 |
| 19 | -54.985 | 21 |
| 20 | -54.898 | 159 |
| 21 | -54.894 | 1125 |
| 22 | -54.639 | 11 |
| 23 | -54.639 | 743 |
| 24 | -54.633 | 47 |
| 25 | -54.604 | 227 |
| 26 | -54.371 | 562 |
| 27 | -54.355 | 87 |
| 28 | -54.275 | 242 |
| 29 | -54.244 | 22 |
| 30 | -54.196 | 27 |
| 31 | -53.979 | 38 |
| 32 | -53.949 | 40 |
| 33 | -53.727 | 37 |
| 34 | -53.716 | 244 |
| 35 | -53.681 | 68 |
| 36 | -53.589 | 114 |
| 37 | -53.495 | 69 |
| 38 | -53.332 | 3 |
| 39 | -53.316 | 61 |
| 40 | -53.228 | 246 |
| 41 | -53.222 | 59 |
| 42 | -53.148 | 21 |
| 43 | -53.094 | 146 |
| 44 | -53.052 | 50 |
| 45 | -53.021 | 8 |
| 46 | -52.98 | 10 |
| 47 | -52.902 | 20 |
| 48 | -52.747 | 354 |
| **Cluster** | **Rosetta**  **total score** | **Size** |
| 49 | -52.745 | 55 |
| 50 | -52.71 | 48 |
| 51 | -52.598 | 334 |
| 52 | -52.462 | 54 |
| 53 | -52.453 | 12 |
| 54 | -52.141 | 141 |
| 55 | -52.134 | 37 |
| 56 | -52.1 | 3 |
| 57 | -52.071 | 13 |
| 58 | -51.695 | 20 |
| 59 | -51.695 | 38 |
| 60 | -51.685 | 3 |
| 61 | -51.63 | 8 |
| 62 | -51.556 | 116 |
| 63 | -51.504 | 50 |
| 64 | -51.454 | 10 |
| 65 | -51.412 | 8 |
| 66 | -51.386 | 19 |
| 67 | -51.316 | 3 |
| 68 | -51.05 | 38 |
| 69 | -50.793 | 9 |
| 70 | -50.666 | 69 |
| 71 | -50.616 | 20 |
| 72 | -50.612 | 7 |
| 73 | -50.594 | 20 |
| 74 | -50.506 | 27 |
| 75 | -50.295 | 20 |
| 76 | -50.213 | 13 |
| 77 | -50.195 | 16 |
| 78 | -50.063 | 40 |
| 79 | -49.919 | 5 |
| 80 | -49.758 | 67 |
| 81 | -49.595 | 2 |
| 82 | -49.585 | 13 |
| 83 | -49.416 | 7 |
| 84 | -49.399 | 5 |
| 85 | -48.889 | 25 |
| 86 | -48.819 | 15 |
| 87 | -48.811 | 34 |
| 88 | -48.75 | 8 |
| 89 | -48.681 | 10 |
| 90 | -48.259 | 38 |
| **Cluster** | **Rosetta**  **total score** | **Size** |
| 91 | -48.188 | 20 |
| 92 | -48.083 | 22 |
| 93 | -47.885 | 34 |
| 94 | -47.836 | 20 |
| 95 | -47.8 | 14 |
| 96 | -47.542 | 20 |
| 97 | -47.417 | 19 |
| 98 | -47.199 | 20 |
| 99 | -46.182 | 17 |
| **nidogen-1 EGF-like4** | | |
| 1 | -75.143 | 2109 |
| 2 | -74.336 | 558 |
| 3 | -73.855 | 234 |
| 4 | -72.306 | 80 |
| 5 | -72.165 | 486 |
| 6 | -71.009 | 22 |
| 7 | -70.502 | 78 |
| 8 | -70.187 | 28 |
| 9 | -69.103 | 30 |
| 10 | -69.077 | 13 |
| 11 | -69.032 | 19 |
| 12 | -68.953 | 20 |
| 13 | -68.681 | 115 |
| 14 | -68.576 | 13 |
| 15 | -66.963 | 11 |
| 16 | -61.702 | 18 |
| 17 | -57.488 | 20 |
| **nidogen-1 EGF-like5** | | |
| 1 | -52.236 | 2097 |
| 2 | -52.007 | 220 |
| 3 | -51.222 | 4592 |
| 4 | -48.413 | 1774 |
| 5 | -48.33 | 101 |
| 6 | -47.94 | 52 |
| 7 | -47.416 | 549 |
| 8 | -46.095 | 190 |
| 9 | -45.777 | 35 |
| 10 | -43.962 | 81 |
| 11 | -43.856 | 65 |
| 12 | -43.659 | 182 |
| 13 | -43.639 | 234 |
| 14 | -42.662 | 110 |
| **Cluster** | **Rosetta**  **total score** | **Size** |
| 15 | -41.721 | 114 |
| 16 | -41.685 | 45 |
| 17 | -41.309 | 28 |
| 18 | -41.096 | 44 |
| 19 | -39.817 | 10 |
| 20 | -38.682 | 40 |
| 21 | -38.152 | 91 |
| 22 | -38.044 | 50 |
| 23 | -37.678 | 59 |
| 24 | -36.402 | 13 |
| 25 | -36.191 | 7 |
| 26 | -35.896 | 28 |
| 27 | -35.576 | 81 |
| 28 | -35.383 | 6 |
| 29 | -34.992 | 42 |
| 30 | -34.66 | 14 |
| 31 | -34.54 | 10 |
| 32 | -33.643 | 12 |
| 33 | -33.58 | 60 |
| 34 | -32.447 | 7 |
| 35 | -30.428 | 3 |
| 36 | -29.382 | 6 |
| 37 | -27.312 | 6 |
| 38 | -13.866 | 2 |
| **nidogen-1 EGF-like6** | | |
| 1 | -444.669 | 262 |
| 2 | -442.071 | 134 |
| 3 | -427.306 | 6 |
| 4 | -418.742 | 39 |
| 5 | -415.983 | 9 |
| 6 | -404.062 | 11 |
| 7 | -38.974 | 24 |
| 8 | -387.185 | 5 |
| 9 | -382.462 | 13 |
| 10 | -375.107 | 7 |
| 11 | -368.118 | 3 |
| 12 | -36.798 | 34 |
| 13 | -356.021 | 4 |
| 14 | -354.445 | 2 |
| 15 | -352.512 | 3 |
| 16 | -348.531 | 3 |
| 17 | -341.111 | 2 |
| **Cluster** | **Rosetta**  **total score** | **Size** |
| 18 | -340.537 | 2 |
| 19 | -330.647 | 2 |
| 20 | -287.444 | 9 |
| 21 | -276.732 | 3 |
| 22 | -243.363 | 5 |
| 23 | -232.201 | 3 |
| 24 | -229.486 | 4 |
| **nidogen-1 TY1** | | |
| 1 | -123.779 | 1673 |
| 2 | -120.223 | 108 |
| 3 | -118.078 | 21 |
| 4 | -115.715 | 42 |
| 5 | -115.462 | 38 |
| 6 | -114.68 | 19 |
| 7 | -110.163 | 43 |
| 8 | -109.364 | 8 |
| 9 | -106.916 | 9 |
| 10 | -106.368 | 12 |
| 11 | -105.873 | 19 |
| 12 | -104.06 | 2 |
| 13 | -103.926 | 2 |
| 14 | -101.87 | 16 |
| 15 | -101.739 | 2 |
| 16 | -101.498 | 3 |
| 17 | -101.058 | 20 |
| 18 | -96.986 | 3 |
| 19 | -95.866 | 11 |
| 20 | -65.183 | 2 |
| **laminin γ1 LEa3** | | |
| 1 | -58.143 | 59 |
| 2 | -56.974 | 88 |
| 3 | -56.131 | 171 |
| 4 | -56.016 | 40 |
| 5 | -55.728 | 91 |
| 6 | -55.402 | 34 |
| 7 | -55.247 | 40 |
| 8 | -55.241 | 28 |
| 9 | -55.093 | 3 |
| 10 | -54.808 | 336 |
| 11 | -54.698 | 41 |
| 12 | -54.616 | 668 |
| 13 | -54.493 | 21 |
| **Cluster** | **Rosetta**  **total score** | **Size** |
| 14 | -54.4 | 236 |
| 15 | -54.354 | 2 |
| 16 | -53.575 | 233 |
| 17 | -53.529 | 30 |
| 18 | -53.312 | 388 |
| 19 | -53.297 | 20 |
| 20 | -53.29 | 58 |
| 21 | -53.05 | 64 |
| 22 | -52.953 | 15 |
| 23 | -52.891 | 559 |
| 24 | -52.348 | 20 |
| 25 | -52.3 | 452 |
| 26 | -52.199 | 20 |
| 27 | -52.197 | 99 |
| 28 | -52.097 | 65 |
| 29 | -51.896 | 94 |
| 30 | -51.734 | 113 |
| 31 | -51.663 | 43 |
| 32 | -51.488 | 36 |
| 33 | -51.434 | 56 |
| 34 | -51.298 | 36 |
| 35 | -51.089 | 2 |
| 36 | -51.06 | 52 |
| 37 | -51.048 | 146 |
| 38 | -50.896 | 29 |
| 39 | -50.861 | 31 |
| 40 | -50.421 | 36 |
| 41 | -50.339 | 16 |
| 42 | -49.817 | 2 |
| 43 | -49.595 | 20 |
| 44 | -49.312 | 40 |
| 45 | -48.757 | 12 |
| 46 | -48.754 | 34 |
| 47 | -47.987 | 11 |
| 48 | -46.581 | 26 |
| 49 | -46.457 | 5 |
| 50 | -44.972 | 2 |
| **laminin γ1 LEa4** | | |
| 1 | -78.277 | 191 |
| 2 | -76.895 | 591 |
| 3 | -76.649 | 1083 |
| 4 | -75.974 | 2723 |
| **Cluster** | **Rosetta**  **total score** | **Size** |
| 5 | -75.678 | 155 |
| 6 | -73.538 | 412 |
| 7 | -72.686 | 78 |
| 8 | -72.654 | 5 |
| 9 | -72.64 | 157 |
| 10 | -72.336 | 100 |
| 11 | -72.003 | 10 |
| 12 | -71.863 | 59 |
| 13 | -71.809 | 29 |
| 14 | -71.659 | 7 |
| 15 | -71.034 | 20 |
| 16 | -69.719 | 51 |
| 17 | -69.357 | 107 |
| 18 | -69.305 | 167 |
| 19 | -68.854 | 65 |
| 20 | -66.733 | 5 |
| 21 | -66.286 | 57 |
| 22 | -64.257 | 33 |
| 23 | -63.67 | 24 |
| 24 | -63.068 | 4 |
| 25 | -62.7 | 4 |
| 26 | -61.248 | 8 |
| 27 | -60.166 | 11 |
| 28 | -59.832 | 9 |
| 29 | -59.063 | 5 |
| 30 | -59.046 | 31 |
| 31 | -58.718 | 6 |
| 32 | -57.926 | 21 |
| 33 | -57.476 | 6 |
| 34 | -57.37 | 15 |
| 35 | -57.276 | 10 |
| 36 | -57.226 | 3 |
| 37 | -57.089 | 4 |
| 38 | -57.083 | 3 |
| 39 | -56.642 | 3 |
| 40 | -56.507 | 6 |
| 41 | -56.495 | 7 |
| 42 | -56.16 | 2 |
| 43 | -55.983 | 2 |
| 44 | -55.945 | 6 |
| 45 | -55.782 | 4 |
| 46 | -55.468 | 3 |
| **Cluster** | **Rosetta**  **total score** | **Size** |
| 47 | -55.465 | 2 |
| 48 | -55.25 | 3 |
| 49 | -55.155 | 3 |
| 50 | -54.955 | 2 |
| 51 | -54.852 | 5 |
| 52 | -53.924 | 3 |
| 53 | -53.913 | 6 |
| 54 | -53.877 | 3 |
| 55 | -53.805 | 3 |
| 56 | -53.797 | 2 |
| 57 | -53.773 | 4 |
| 58 | -53.195 | 2 |
| 59 | -53.128 | 2 |
| 60 | -53.099 | 2 |
| 61 | -52.84 | 2 |
| 62 | -52.45 | 2 |
| 63 | -52.323 | 2 |
| 64 | -52.141 | 4 |
| 65 | -52.06 | 4 |
| 66 | -51.84 | 4 |
| 67 | -51.19 | 2 |
| 68 | -51.186 | 2 |
| 69 | -50.787 | 2 |
| 70 | -50.191 | 4 |
| 71 | -49.468 | 2 |
| 72 | -48.671 | 3 |
| 73 | -42.65 | 2 |
| **laminin γ1 LEa5.2** | | |
| 1 | -51.093 | 213 |
| 2 | -49.005 | 3150 |
| 3 | -47.76 | 374 |
| 4 | -47.025 | 154 |
| 5 | -45.572 | 328 |
| 6 | -45.403 | 123 |
| 7 | -45.312 | 556 |
| 8 | -45.3 | 298 |
| 9 | -44.941 | 241 |
| 10 | -44.621 | 62 |
| 11 | -44.3 | 122 |
| 12 | -44.192 | 550 |
| 13 | -44.027 | 57 |
| 14 | -43.773 | 41 |
| **Cluster** | **Rosetta**  **total score** | **Size** |
| 15 | -43.597 | 47 |
| 16 | -43.52 | 136 |
| 17 | -43.274 | 68 |
| 18 | -43.084 | 19 |
| 19 | -42.63 | 159 |
| 20 | -42.292 | 393 |
| 21 | -41.78 | 41 |
| 22 | -41.533 | 194 |
| 23 | -40.815 | 6 |
| 24 | -40.742 | 140 |
| 25 | -40.581 | 116 |
| 26 | -40.333 | 67 |
| 27 | -40.015 | 4 |
| 28 | -39.805 | 40 |
| 29 | -38.968 | 25 |
| 30 | -38.966 | 34 |
| 31 | -36.82 | 44 |
| 32 | -36.745 | 3 |
| 33 | -36.719 | 37 |
| 34 | -36.341 | 12 |
| 35 | -36.192 | 12 |
| 36 | -35.309 | 16 |
| 37 | -34.989 | 5 |
| 38 | -34.287 | 11 |
| 39 | -31.598 | 25 |
| 40 | -30.449 | 6 |
| 41 | -29.886 | 15 |
| 42 | -28.149 | 12 |
| 43 | -27.196 | 19 |
| 44 | -25.043 | 5 |
| 45 | -19.675 | 6 |
| 46 | 1.101 | 2 |
| 47 | 1.535 | 5 |
| **laminin γ1 LEb1** | | |
| 1 | -64.961 | 3381 |
| 2 | -63.707 | 659 |
| 3 | -62.673 | 352 |
| 4 | -61.324 | 5 |
| 5 | -61.109 | 35 |
| 6 | -60.973 | 79 |
| 7 | -60.655 | 145 |
| 8 | -60.314 | 7 |
| **Cluster** | **Rosetta**  **total score** | **Size** |
| 9 | -59.446 | 64 |
| 10 | -59.139 | 113 |
| 11 | -58.717 | 56 |
| 12 | -58.636 | 121 |
| 13 | -58.089 | 264 |
| 14 | -57.682 | 7 |
| 15 | -57.602 | 63 |
| 16 | -57.219 | 23 |
| 17 | -56.593 | 19 |
| 18 | -56.188 | 92 |
| 19 | -55.925 | 54 |
| 20 | -55.745 | 20 |
| 21 | -55.643 | 4 |
| 22 | -54.345 | 30 |
| 23 | -53.166 | 2 |
| 24 | -52.942 | 3 |
| 25 | -52.617 | 31 |
| 26 | -51.676 | 3 |
| 27 | -51.249 | 5 |
| 28 | -51.167 | 13 |
| 29 | -51.13 | 2 |
| 30 | -50.992 | 30 |
| 31 | -50.963 | 3 |
| 32 | -50.876 | 5 |
| 33 | -50.769 | 2 |
| 34 | -50.635 | 2 |
| 35 | -50.488 | 15 |
| 36 | -50.275 | 20 |
| 37 | -50.009 | 5 |
| 38 | -49.99 | 50 |
| 39 | -49.578 | 46 |
| 40 | -49.333 | 30 |
| 41 | -49.25 | 5 |
| 42 | -48.888 | 12 |
| 43 | -48.885 | 17 |
| 44 | -48.654 | 12 |
| 45 | -48.103 | 41 |
| 46 | -47.742 | 4 |
| 47 | -47.54 | 5 |
| 48 | -47.358 | 3 |
| 49 | -47.052 | 4 |
| 50 | -45.633 | 3 |
| **Cluster** | **Rosetta**  **total score** | **Size** |
| 51 | -45.625 | 4 |
| 52 | -45.615 | 2 |
| 53 | -45.191 | 3 |
| 54 | -45.176 | 7 |
| 55 | -45.004 | 11 |
| 56 | -44.727 | 2 |
| 57 | -44.38 | 6 |
| 58 | -44.049 | 4 |
| 59 | -43.928 | 3 |
| 60 | -42.907 | 16 |
| 61 | -42.781 | 2 |
| 62 | -42.542 | 3 |
| 63 | -42.337 | 10 |
| 64 | -41.762 | 3 |
| 65 | -40.707 | 6 |
| 66 | -39.885 | 4 |
| 67 | -39.675 | 5 |
| 68 | -39.26 | 4 |
| 69 | -39.144 | 2 |
| 70 | -38.888 | 4 |
| 71 | -38.792 | 2 |
| 72 | -33.967 | 2 |
| 73 | -31.011 | 8 |
| **laminin γ1 LEb5** | | |
| 1 | -68.966 | 2407 |
| 2 | -68.305 | 259 |
| 3 | -68.12 | 66 |
| 4 | -66.273 | 102 |
| 5 | -65.832 | 407 |
| 6 | -65.77 | 58 |
| 7 | -65.432 | 59 |
| 8 | -65.052 | 10 |
| 9 | -64.376 | 336 |
| 10 | -63.547 | 138 |
| 11 | -63.28 | 151 |
| 12 | -62.321 | 67 |
| 13 | -62.143 | 104 |
| 14 | -61.74 | 91 |
| 15 | -61.068 | 42 |
| 16 | -60.842 | 18 |
| 17 | -59.751 | 18 |
| 18 | -58.946 | 7 |
| **Cluster** | **Rosetta**  **total score** | **Size** |
| 19 | -56.836 | 7 |
| 20 | -56.395 | 37 |
| 21 | -56.2 | 63 |
| 22 | -55.609 | 5 |
| 23 | -55.515 | 21 |
| 24 | -54.243 | 9 |
| 25 | -53.078 | 15 |
| 26 | -52.378 | 15 |
| 27 | -52.356 | 4 |
| 28 | -51.848 | 17 |
| 29 | -51.202 | 2 |
| 30 | -49.306 | 2 |
| 31 | -49.075 | 23 |
| 32 | -48.874 | 20 |
| 33 | -48.685 | 5 |
| 34 | -46.971 | 33 |
| 35 | -45.912 | 4 |
| 36 | -45.161 | 20 |
| 37 | -44.687 | 4 |
| 38 | -42.222 | 2 |
| 39 | -28.486 | 20 |
| 40 | -28.059 | 20 |
| 41 | -18.496 | 19 |
