# Supplementary material for: Analysis of Nidogen-1/Laminin γ1 Interaction by Cross-Linking, Mass Spectrometry, and Computational Modeling Reveals Multiple Binding Modes
Source: PLoS One. 2014 Nov 11;9(11):e112886. doi: 10.1371/journal.pone.0112886 (PMC4227867; doi:10.1371/journal.pone.0112886)
Supplement: Table S2 — Results of Rosetta clustering of the laminin γ1 L4 domain models. The best 10% of all generated models were clustered using a clustering radius of 1 Å. Shown are clusters with a size >1. The best-scoring models of clusters 1 and 2 were chosen as final models of the L4 domain. (DOC) [file pone.0112886.s011.doc]

Table S 2. Results of Rosetta clustering of the laminin γ1 L4 domain models. The best 10% of all generated models were clustered using a clustering radius of 1 Å. Shown are clusters with a size >1. The best-scoring models of clusters 1 and 2 were chosen as final models of the L4 domain.

| **Cluster** | **Rosetta total score** | **Size** |
| --- | --- | --- |
| **1** | -334.551 | 26 |
| **2** | -333.168 | 9 |
| **3** | -326.311 | 5 |
| **4** | -325.444 | 2 |
| **5** | -322.787 | 8 |
| **6** | -322.735 | 12 |
| **7** | -322.153 | 2 |
| **8** | -320.378 | 5 |
| **9** | -319.837 | 31 |
| **10** | -319.825 | 28 |
| **11** | -318.882 | 9 |
| **12** | -318.439 | 22 |
| **13** | -318.322 | 2 |
| **14** | -317.868 | 3 |
| **15** | -317.747 | 7 |
| **16** | -317.734 | 3 |
| **17** | -317.557 | 2 |
| **18** | -317.339 | 6 |
| **19** | -316.799 | 2 |
| **20** | -315.946 | 6 |
| **21** | -315.562 | 3 |
| **22** | -315.482 | 5 |
| **23** | -314.987 | 13 |
| **24** | -314.909 | 2 |
| **25** | -314.827 | 21 |
| **26** | -314.778 | 2 |
| **27** | -314.491 | 4 |
| **28** | -326.83 | 8 |
| **29** | -322.35 | 2 |
| **30** | -320.29 | 4 |
