# Supplementary material for: Analysis of Nidogen-1/Laminin γ1 Interaction by Cross-Linking, Mass Spectrometry, and Computational Modeling Reveals Multiple Binding Modes
Source: PLoS One. 2014 Nov 11;9(11):e112886. doi: 10.1371/journal.pone.0112886 (PMC4227867; doi:10.1371/journal.pone.0112886)
Supplement: Table S3 — Scores of the final nidogen-1 NIDO domain models. Models sharing the ‘centroid model identifier’ originate from the same initial low-resolution centroid model. Models, for which structural homologues within the PDB have been identified, are listed in italics. The remaining models share a similar topology to models generated based on highly homologous sequences of NIDO domains derived from related organisms. (DOC) [file pone.0112886.s012.doc]

Table S 3. Scores of the final nidogen-1 NIDO domain models. Models sharing the ‘centroid model identifier’ originate from the same initial low-resolution centroid model. Models, for which structural homologues within the PDB have been identified, are listed in italics. The remaining models share a similar topology to models generated based on highly homologous sequences of NIDO domains derived from related organisms.

| **Model** | **Centroid model identifier** | **Rosetta total score** |
| --- | --- | --- |
| **1** | S_0035_17 | -319.502 |
| **2** | S_0037_97 | -317.883 |
| **3** | S_0035_17 | -317.623 |
| **4** | S_0035_17 | -317.248 |
| **5** | S_0037_97 | -316.973 |
| **6** | S_0117_45 | -316.904 |
| **7** | S_0035_17 | -315.975 |
| **8** | S_0117_45 | -315.762 |
| **9** | S_0118_37 | -315.713 |
| **10** | S_0037_97 | -315.200 |
| **11** | S_0060_97 | -314.867 |
| **12** | S_0071_9 | -314.258 |
| **13** | S_0118_37 | -313.856 |
| **14** | S_0040_63 | -312.260 |
| **15** | S_0058_71 | -311.148 |
| **16** | S_0053_97 | -310.042 |
| **17** | S_0025_15 | -309.495 |
| **18** | S_0027_64 | -309.394 |
| **19** | S_0017_40 | -309.039 |
| **20** | S_0002_99 | -308.752 |
| **21** | S_0083_76 | -308.191 |
| **22** | S_0013_65 | -307.592 |
| **23** | S_0069_16 | -307.077 |
| ***24*** | *S_0096_32* | *-306.554* |
| ***25*** | *S_0018_74* | *-306.444* |
| ***26*** | *S_0018_74* | *-305.892* |
| ***27*** | *S_0018_74* | *-305.763* |
| **28** | S_0067_3 | -305.196 |
| **29** | S_0003_7 | -304.848 |
| ***30*** | *S_0096_32* | *-304.724* |
| ***31*** | *S_0018_74* | *-304.088* |
| ***32*** | *S_0096_32* | *-303.815* |
| **33** | S_0012_60 | -303.457 |
| **34** | S_0033_98 | -303.376 |
| ***35*** | *S_0018_74* | *-303.298* |
| **36** | S_0011_27 | -303.230 |
| **37** | S_0093_23 | -303.048 |
| **38** | S_0025_76 | -302.989 |
| ***39*** | *S_0096_32* | *-302.912* |
| **Model** | Centroid model identifier | Rosetta total score |
| **40** | S_0137 | -302.337 |
| ***41*** | *S_0096_32* | *-302.254* |
| ***42*** | *S_0096_32* | *-302.222* |
| **43** | S_0001_63 | -302.077 |
| **44** | S_0115_37 | -301.796 |
| **45** | S_0048_18 | -301.499 |
| **46** | S_0138_19 | -301.308 |
| **47** | S_0055_45 | -301.233 |
| **48** | S_0121_44 | -301.020 |
| **49** | S_0046_41 | -300.602 |
| **50** | S_0088_10 | -300.364 |
| **51** | S_0039_20 | -300.332 |
| **52** | S_0006_21 | -300.026 |
| ***53*** | *S_0096_32* | *-299.992* |
| **54** | S_0001_49 | -299.967 |
| **55** | S_0016_7 | -299.462 |
| **56** | S_0019_65 | -299.436 |
| **57** | S_0089_48 | -299.100 |
| **58** | S_0068_47 | -298.835 |
| **59** | S_0115_4 | -298.718 |
| **60** | S_0044_82 | -298.523 |
| **61** | S_0010_14 | -298.332 |
| **62** | S_0073_10 | -298.248 |
| **63** | S_0049_92 | -298.036 |
| **64** | S_0097_34 | -298.017 |
| **65** | S_0002_51 | -297.770 |
| **66** | S_0017_2 | -297.727 |
| ***67*** | *S_0018_74* | *-297.722* |
| **68** | S_0034_97 | -297.400 |
| **69** | S_0096_11 | -296.825 |
| ***70*** | *S_0096_32* | *-296.673* |
| **71** | S_0035_33 | -296.666 |
| **72** | S_0144_6 | -296.475 |
| **73** | S_0137_18 | -296.386 |
| **74** | S_0036_58 | -296.270 |
| **75** | S_0070_45 | -296.229 |
| ***76*** | *S_0018_74* | *-296.226* |
| **77** | S_0039_31 | -295.331 |
| **78** | S_0018_58 | -295.197 |
| **79** | S_0072_58 | -295.185 |
| **80** | S_0109_63 | -294.888 |
| ***81*** | *S_0018_74* | *-294.731* |
| **Model** | Centroid model identifier | Rosetta total score |
| **82** | S_0001 | -294.593 |
| **83** | S_0070_21 | -294.474 |
| **84** | S_0010_11 | -293.891 |
| **85** | S_0006_50 | -293.782 |
| **86** | S_0009_2 | -293.701 |
| **87** | S_0078_27 | -293.667 |
| **88** | S_0139_4 | -293.612 |
| **89** | S_0038_67 | -293.515 |
| **90** | S_0047_77 | -293.285 |
| **91** | S_0078_3 | -293.142 |
| **92** | S_0027_16 | -293.050 |
| **93** | S_0127_21 | -292.603 |
| **94** | S_0046_39 | -292.052 |
| **95** | S_0025_23 | -291.678 |
| **96** | S_0094_54 | -291.548 |
| **97** | S_0095_37 | -291.517 |
| **98** | S_0058_89 | -290.914 |
| **99** | S_0055_94 | -290.565 |
| **100** | S_0092_78 | -290.344 |
| **101** | S_0060_1 | -290.101 |
| **102** | S_0022_70 | -287.532 |
| **103** | S_0086_6 | -287.376 |
| **104** | S_0063_21 | -286.882 |
| **105** | S_0024_96 | -286.562 |
| **106** | S_0002_69 | -286.369 |
| **107** | S_0023_67 | -286.042 |
| **108** | S_0043_68 | -285.961 |
| **109** | S_0055_78 | -285.826 |
| **110** | S_0078_7 | -285.716 |
| **111** | S_0119_5 | -285.147 |
| **112** | S_0101_64 | -285.034 |
| **113** | S_0095_46 | -284.777 |
| **114** | S_0020 | -284.761 |
| **115** | S_0127_45 | -284.255 |
| **116** | S_0016_61 | -284.055 |
| **117** | S_0016_5 | -283.682 |
| **118** | S_0051_92 | -282.692 |
| **119** | S_0055_3 | -280.267 |
| **120** | S_0037_44 | -279.975 |
| **121** | S_0041_63 | -279.309 |
| **122** | S_0059_75 | -278.156 |
| **123** | S_0081_36 | -278.032 |
| **124** | S_0042_22 | -277.905 |
| **Model** | Centroid model identifier | Rosetta total score |
| **125** | S_0091_15 | -277.608 |
| **126** | S_0085_43 | -276.726 |
| **127** | S_0088_22 | -275.940 |
| **128** | S_0004_82 | -274.762 |
| **129** | S_0025_84 | -273.301 |
| **130** | S_0017_29 | -269.681 |
| **131** | S_0056_42 | -267.916 |
| **132** | S_0068_60 | -261.762 |
