# Supplementary material for: Analysis of Nidogen-1/Laminin γ1 Interaction by Cross-Linking, Mass Spectrometry, and Computational Modeling Reveals Multiple Binding Modes
Source: PLoS One. 2014 Nov 11;9(11):e112886. doi: 10.1371/journal.pone.0112886 (PMC4227867; doi:10.1371/journal.pone.0112886)
Supplement: Table S4 — Scores of the final models of the nidogen-1 G3/laminin γ1 LEb2–4 complex. The listed models rank among the top 20 of 800 generated models considering both Rosetta total score and atom pair constraint score, which reflects their compliance with the cross-linking distance constraints. (DOC) [file pone.0112886.s013.doc]

Table S 4. Scores of the final models of the nidogen-1 G3/laminin γ1 LEb2–4 complex. The listed models rank among the top 20 of 800 generated models considering both Rosetta total score and atom pair constraint score, which reflects their compliance with the cross-linking distance constraints.

| **Model** | **Rosetta**  **total score** | **Atom pair**  **constraint score** |
| --- | --- | --- |
| **1** | -863.595 | 26.603 |
| **2** | -862.411 | 25.434 |
| **3** | -854.517 | 27.195 |
| **4** | -853.083 | 27.286 |
| **5** | -852.755 | 21.404 |
| **6** | -852.516 | 26.367 |
| **7** | -849.500 | 30.405 |
| **8** | -848.419 | 26.798 |
| **9** | -848.166 | 26.465 |
| **10** | -845.955 | 24.959 |
