# Supplementary material for: Analysis of Nidogen-1/Laminin γ1 Interaction by Cross-Linking, Mass Spectrometry, and Computational Modeling Reveals Multiple Binding Modes
Source: PLoS One. 2014 Nov 11;9(11):e112886. doi: 10.1371/journal.pone.0112886 (PMC4227867; doi:10.1371/journal.pone.0112886)
Supplement: Table S5 — Affinities and kinetic parameters of the nidogen-1/laminin γ1 interaction. For SPR measurements, given values for ka and kd are the weighted mean from two individual measurements and Kd was calculated from these values as Kd = kd/ka. All ELISA-based measurements were performed in triplicates and Kd values were determined by non-linear regression of the saturation binding curves. The values in parentheses represent standard deviations. (DOC) [file pone.0112886.s014.doc]

Table S 5. Affinities and kinetic parameters of the nidogen-1/laminin γ1 interaction. For SPR measurements, given values for k_a_ and k_d_ are the weighted mean from two individual measurements and K_d_ was calculated from these values as K_d_=k_d_/k_a_. All ELISA-based measurements were performed in triplicates and K_d_ values were calculated based on a sigmoidal fit of the saturation binding curves. The values in parentheses represent standard deviations.

| **Nidogen-1 interaction partner** | **SPR** | | | **ELISA** |
| --- | --- | --- | --- | --- |
|  | **k_d_ (s^-1^)** | **k_a_ (M^-1^ * s^-1^)** | **K_d_ (nM)** | **K_d_ (nM)** |
| Laminin γ1  short arm | 1.3 (± 0.1) * 10^-4^ | 1.1 (± 0.0) * 10^4^ | 12 (± 1) | 1.1 (± 0.1) |
| Laminin γ1  LEb2–4 | 9.7 (± 1.3) * 10^-5^ | 3.6 (± 0.2) * 10^5^ | 0.27 (± 0.05) | 1.4 (± 0.1) |
| Laminin γ1  short arm N836D | - | - | - | 34 (± 2) |
| Laminin γ1  LEb2–4 N836D | - | - | - | 45 (± 7) |
