# Supplementary material for: Analysis of Nidogen-1/Laminin γ1 Interaction by Cross-Linking, Mass Spectrometry, and Computational Modeling Reveals Multiple Binding Modes
Source: PLoS One. 2014 Nov 11;9(11):e112886. doi: 10.1371/journal.pone.0112886 (PMC4227867; doi:10.1371/journal.pone.0112886)
Supplement: Table S6 — Verified products of BS2G-mediated cross-linking. Peptide sequences written in parentheses are part of the protein affinity tags and do thus not belong to the native amino acid sequences of the proteins studied. Oxidized methionines within the peptide sequences are denoted with ‘m’. Loss of water or ammonia is indicated by addition of ‘−H2O’ or ‘−NH3’ to the fragment ion. (DOC) [file pone.0112886.s015.doc]

Table S 6. Verified products of BS²G-mediated cross-linking. Peptide sequences written in parentheses are part of the protein affinity tags and do thus not belong to the native amino acid sequences. Oxidized methionines within the peptide sequences are denoted with ‘m’. Loss of water or ammonia is indicated by addition of ‘-H_2_O’ or ‘-NH_3_’ to the fragment ion.

| ***m/z***  **exp.** | **[M+H]^+^**  **calc.** | **charge**  **state** | **Δ [M+H]^+^**  **(ppm)** | **cross-linked**  **lysines** | **peptide sequences** | **identified fragment ions** |
| --- | --- | --- | --- | --- | --- | --- |
| **intra-molecular nidogen -1 cross-links** | | | | | | |
| 511.2829 | 2042.1103 | 4 | -0.2 | K-953 x K-948 | α 949KTEAKAFLHIPAK | b6; b7-H2O; b8; b8-H2O; b9; b9-H2O; b10-NH3; b10; b12-NH3; y1; y3; y4; y5; y10; y10-H2O; y11; y12-NH3 |
|  |  |  |  |  | *β 945NTMK* | *y2; y3; y3-H2O; precursor-NH3-NH3* |
| 515.2818 | 2058.1052 | 4 | 0.1 | K-953 x K-948 | α 949KTEAKAFLHIPAK | b6; b9-NH3; y4-NH3-NH3; y4-H2O-NH3-NH3; y6; y6-H2O; y7-H2O; y8-NH3; y12-NH3 |
|  |  |  |  |  | *β 945NTmK* | *y1; y2; y3; y3-H2O; y4-NH3-NH3; precursor-NH3-NH3; precursor-H2O-NH3-NH3* |
| 653.3301 | 2610.3053 | 4 | -2.5 | K-1072 x K-420 | α 1069DNPKIETSHMDGTNRR | b7-H2O-H2O; b12; y14-H2O |
|  |  |  |  |  | *β 417VNGKVK* | *b5; y4; y4-H2O* |
| 657.3288 | 2626.3002 | 4 | -2.6 | K-1072 x K-420 | α 1069DNPKIETSHmDGTNRR | b7-H2O-H2O; b12; b15; y13-NH3-NH3; y14-H2O |
|  |  |  |  |  | *β 417VNGKVK* | *y4; y4-H2O* |
| 876.1032 | 2626.3002 | 3 | -1.9 | K-1072 x K-1128 | α 1069DNPKIETSHmDGTNRR | b8-NH3; b12; b15; y3-NH3; y5; y14; y14-H2O |
|  |  |  |  |  | *β 1127RKVLE* | *b2; b2-H2O; b3-H2O* |
| 602.9203 | 3010.5673 | 5 | -1.8 | K-420 x K-949 | α 407QCVAEGSPQRVNGK | b7-NH3; b11-NH3-NH3; y1-H2O; y2-H2O; y3; y3-H2O; y4; y5; y8; y10-NH3; y11-NH3; y12; y12-H2O; y13 |
|  |  |  |  |  | *β 939RLPLERNTMKK* | *b5-H2O; b8-NH3; y1; y2; y4; y8; y8-H2O-H2O; y9-H2O; y10-H2O; precursor-H2O-H2O-H2O; precursor-H2O-H2O-H2O-NH3* |

| ***m/z***  **exp.** | **[M+H]^+^**  **calc.** | **charge**  **state** | **Δ [M+H]^+^**  **(ppm)** | **cross-linked**  **lysines** | **peptide sequences** | **identified fragment ions** |
| --- | --- | --- | --- | --- | --- | --- |
| 781.1239 | 3121.4796 | 4 | -1.9 | K-1072 x K-1032 | α 1059GNLYWTDWNRDNPKIE | b12; b14-H2O-NH3-NH3; b15-NH3-NH3-NH3; y6; y7-H2O-NH3; y13; y13-H2O |
|  |  |  |  |  | *β 1030VAKMDGTQR* | *y7-NH3; y8* |
| 831.9376 | 3324.7191 | 4 | 2.8 | K-1128 x K-421 | α 1128KVLEGLQYPFAVTSYGKNLYYTDWK | b7; b10; b13; b15-H2O-NH3; b18-H2O; b20; b22; y12-H2O; y13; y16; y18; y20; y20-NH3; y20-H2O-H2O; y21-NH3-NH3; y22; y24-NH3 |
|  |  |  |  |  | *β 421VK* | *y1-NH3-NH3-NH3* |
| 803.5946 | 4013.9352 | 5 | 2.2 | K-420 x K-1128 | α 397CVANYTGNGRQCVAEGSPQRVNGK | b6; b7; b9; b14; b18; b19; y1; y8-H2O; y12; y14-H2O-H2O; y19-NH3; y21-H2O-H2O |
|  |  |  |  |  | *β 1118CLNPAQPGRRK* | *y3-H2O-NH3; y7* |
| 849.6967 | 3395.7562 | 4 | 2.6 | K-1152 x K-1032 | α 1128KVLEGLQYPFAVTSYGKNLYYTDWK | b9; b14-H2O; b14-NH3; b16-H2O; b17-H2O; b19-H2O; b19-H2O-H2O; b21-NH2; b22-H2O; y8; y16; y16-NH3; y17; y17-NH3; y18-NH3; y20; y22-H2O; y22-NH3 |
|  |  |  |  |  | *β 1030VAK* | *y1-H2O; y2; y2-NH3* |
| 660.9945 | 3960.9284 | 6 | 0.6 | K-627 x K-949 | α 601CAHDDARPALPSTQQLSVDSVFVLYNKEE | b4-NH3; b6-H2O; b15-H2O; b18-H2O; b19-H2O-H2O; b21; b24-NH3; b25-NH3; b26; y12; y15-H2O; y23; y28-H2O |
|  |  |  |  |  | *β 949KTEAK* | *b3; b3-H2O; precursor-NH3* |
| 897.4711 | 5379.7987 | 6 | -1.6 | K-1072 x K-961 | α 1051GIVTDPVRGNLYWTDWNRDNPKIE | b14-H2O; b18-NH3; y5-NH3; y13-H2O; y14-H2O; y16; y19 |
|  |  |  |  |  | *β 952AKAFLHIPAKVIIGLAFDCVDK* | *b16; b17-NH3; y17-NH3; y18-H2O; y21-NH3* |
| 657.3292 | 2626.3002 | 4 | -2.0 | K-1072 x K-1128 | α 1069DNPKIETSHmDGTNRR | b8-NH3; b12; b15; y14; y14-H2O |
|  |  |  |  |  | *β 1127RKVLE* | *b2; b2-H2O; b3-H2O; precursor-NH3-NH3-NH3* |

| ***m/z***  **exp.** | **[M+H]^+^**  **calc.** | **charge**  **state** | **Δ [M+H]^+^**  **(ppm)** | **cross-linked**  **lysines** | **peptide sequences** | **identified fragment ions** |
| --- | --- | --- | --- | --- | --- | --- |
| 792.9920 | 3960.9252 | 5 | 1.4 | K-1165 x K-194 | α 1145NLYYTDWKTNSVIAMDLAISKE | b15; b17-NH3; b18-NH3-NH3; b21; b21-NH3; b21-NH3-NH3; y6-H2O; y16-H2O; y19-H2O-H2O; y20-H2O |
|  |  |  |  |  | *β 184DGLQFFTTFSK* | *y4; y6-H2O; precursor-H2O-NH3-NH3* |
| 990.9879 | 3960.9284 | 4 | 0.4 | K-627 x K-949 | α 601CAHDDARPALPSTQQLSVDSVFVLYNKEE | b23-H2O; y3-H2O; y16-NH3-NH3; y17-H2O; y24-NH3; y26-NH3; y27; y28-NH3 |
|  |  |  |  |  | *β 949KTEAK* | *b2-NH3; b3-NH3; b4; y2* |
| 719.3855 | 2156.1458 | 3 | -1.8 | K-208 x K-420 | α 198SQVPAVVGFSK | b3; b6; b6-NH3; b7; b10; y7; y7-NH3; y7-H2O-NH3; y8; y8-H2O; y9; y9-NH3; y10-NH3-NH3 |
|  |  |  |  |  | *β 412GSPQRVNGK* | *y6-H2O; y7-H2O; precursor-H2O-NH3; precursor-H2O-NH3-NH3* |
| 602.9207 | 3010.5673 | 5 | 2.4 | K-420 x K-949 | α 407QCVAEGSPQRVNGK | b7-NH3; b11-H2O-H2O; y3; y4; y5; y6-NH3; y8; y11-H2O; y12; y13 |
|  |  |  |  |  | *β 939RLPLERNTMKK* | *b5-NH3; y1; y2; y7; y8* |
| 602.9211 | 3010.5673 | 5 | -0.5 | K-420 x K-949 | α 407QCVAEGSPQRVNGK | b7-NH3; b11-H2O-H2O; y3; y4; y5; y6-NH3; y8; y11-H2O; y12; y13 |
|  |  |  |  |  | *β 939RLPLERNTMKK* | *b5-NH3; y1; y2; y7; y8* |
| 803.5951 | 4013.9352 | 5 | 2.8 | K-420 x K-1128 | α 397CVANYTGNGRQCVAEGSPQRVNGK | b7; b9; b11; b14; b17; b19; y1; y8; y14-H2O-H2O; y15; y19 |
|  |  |  |  |  | *β 1118CLNPAQPGRRK* | *y7; y7-H2O* |
| 539.7906 | 2156.1458 | 4 | -2.4 | K-208 x K-420 | α 198SQVPAVVGFSK | b2; b3; b5-H2O; b6-NH3; b7; b8-H2O; y7-H2O-H2O; y8-H2O; y9; y9-H2O; y10-NH3 |
|  |  |  |  |  | *β 412GSPQRVNGK* | *b5; b7-H2O-H2O; b7-H2O-NH3,y1-NH3; y3; y3-NH3; y7-H2O,precursor-H2O-NH3* |

| ***m/z*** | **[M+H]^+^** | **charge**  **state** | **Δ [M+H]^+^** | **cross-linked lysines** | **peptide sequences** | **identified fragment ions** |
| --- | --- | --- | --- | --- | --- | --- |
| 541.7628 | 2164.0307 | 4 | -0.6 | K-1032 x K-829 | α 1030VAKMDGTQRR | b4; b5-H2O; b8-NH3; y3-NH3; y4-NH3-NH3 |
|  |  |  |  |  | *β 822CMPGEVSK* | *b5-H2O; y1-H2O; y3; y6; y6-NH3; y6-H2O-H2O; y7; y7-H2O* |
| 786.9747 | 3930.8466 | 5 | -0.5 | K-829 x K-949 | α 813PGYQGDGFRCMPGEVSKTRCQLER | b9-H2O; b13-H2O; b14-NH3; b18; b18-H2O; b20; b21; y6-NH3; y7-H2O-NH3; y9-H2O-NH3; y9-NH3-NH3; y13; y15; y16-NH3; y16-H2O-NH3; y23-H2O-NH3 |
|  |  |  |  |  | *β 944RNTMKKTE* | *b5; b6-NH3-NH3; b7-H2O; b7-NH3; y3; precursor-H2O-H2O-NH3* |
| 792.9927 | 3960.9252 | 5 | 2.4 | K-1165 x K-194 | α 1145NLYYTDWKTNSVIAMDLAISKE | b12; b15; b17-NH3; b21; b21-NH3; y4; y6-H2O; y10-H2O; y16-H2O; y19-H2O-NH3; y20; y20-H2O |
|  |  |  |  |  | *β 184DGLQFFTTFSK* | *y4; y5; y6-H2O; y9; precursor* |
| 1004.2422 | 4013.9352 | 4 | 3.0 | K-420 x K-1128 | α 397CVANYTGNGRQCVAEGSPQRVNGK | b11-H2O; b13-NH3; b14; b17-H2O-H2O; y4; y5-H2O-NH3; y8; y8-NH3; y15 |
|  |  |  |  |  | *β 1118CLNPAQPGRRK* | *b9-H2O; y3; y5-NH3-NH3; y7; y7-H2O; y7-H2O-H2O* |
| 602.9203 | 3010.5673 | 5 | 1.7 | K-420 x K-949 | α 407QCVAEGSPQRVNGK | b7-NH3; b11-NH3-NH3; y2; y3; y3-H2O; y4; y5; y8; y11-NH3; y12-H2O; y12-NH3; y13 |
|  |  |  |  |  | *β 939RLPLERNTMKK* | *b5-H2O; b8-NH3; y1; y2; y5-H2O; y8; y8-H2O-H2O; y9-NH3* |
| 792.9923 | 3960.9252 | 5 | 1.8 | K-1165 x K-194 | α 1145NLYYTDWKTNSVIAMDLAISKE | b12; b15; b17-NH3; b18-NH3-NH3; b21; b21-H2O; y4; y9-NH3; y10; y16-H2O; y18-NH3-NH3; y19-NH3-NH3; y20; y20-H2O |
|  |  |  |  |  | *β 184DGLQFFTTFSK* | *y4; y5; y6-NH3; y9; y10-H2O* |
| ***m/z*** | **[M+H]^+^** | **charge**  **state** | **Δ [M+H]^+^** | **cross-linked lysines** | **peptide sequences** | **identified fragment ions** |
| 803.5943 | 4013.9352 | 5 | 1.8 | K-420 x K-1128 | α 397CVANYTGNGRQCVAEGSPQRVNGK | b6; b7; b9; b11-NH3; b14; b17-NH3; y1; y5-H2O-NH3; y14-H2O-H2O; y15; y19-H2O; y19-NH3; y21-H2O-H2O |
|  |  |  |  |  | *β 1118CLNPAQPGRRK* | *b9; b9-H2O-H2O; y3-NH3-NH3; y7; y7-NH3* |
| 792.9921 | 3960.9252 | 5 | 1.5 | K-1165 x K-194 | α 1145NLYYTDWKTNSVIAMDLAISKE | b15; b16-NH3; b21; b21-NH3; y6-H2O; y16-H2O; y19-H2O-H2O; y20; y20-H2O |
|  |  |  |  |  | *β 184DGLQFFTTFSK* | *b7-H2O-NH3; y4; y6-H2O; y9* |
| 803.5947 | 4013.9352 | 5 | 2.3 | K-420 x K-1128 | α 397CVANYTGNGRQCVAEGSPQRVNGK | b7; b8; b9; b14; b17; b18; b19; y8; y15; y19-NH3 |
|  |  |  |  |  | *β 1118CLNPAQPGRRK* | *y7; y7-NH3* |
| 687.3571 | 4119.1179 | 6 | -2.8 | K-627 x K-1032 | α 608PALPSTQQLSVDSVFVLYNKEER | b13-H2O; b16-H2O; b20; y5; y13-NH3; y21-NH3 |
|  |  |  |  |  | *β 1028IEVAKMDGTQRR* | *b5-H2O; y6-NH3; y7; y10; y11-NH3* |
| 919.6780 | 4594.3690 | 5 | -1.7 | K-1152 x K-948 | α 1128KVLEGLQYPFAVTSYGKNLYYTDWK | b9-H2O; b10-H2O; b14; b24; y1-H2O-H2O; y3-NH3; y6; y11; y18; y19; y22 |
|  |  |  |  |  | *β 937IERLPLERNTmK* | *y8; y9; y10* |
| 849.9208 | 5094.5014 | 6 | -2.6 | K-1032 x K 489 | α 1008GIALDHLGRTIFWTDSQLDRIEVAKMDGTQR | b16-NH3; b18; b22; b26-H2O; b29; y6-H2O; y15-H2O; y18-NH3; y27 |
|  |  |  |  |  | *β 485QDGFKNGFSITGGE* | *b7-NH3; b10; b11-H2O; b13-NH3; y11* |
| 792.9923 | 3960.9252 | 5 | 1.8 | K-1165 x K-194 | α 1145NLYYTDWKTNSVIAMDLAISKE | b12; b15; b16-NH3; b17-NH3; b18-NH3-NH3; b21-NH3; y4; y6-H2O,y16-H2O; y19-H2O-NH3; y20-H2O |
|  |  |  |  |  | *β 184DGLQFFTTFSK* | *y4; y5; y6-H2O* |
| 988.8016 | 4939.9923 | 5 | -2.7 | K-812 x K-195 | α 785DVDECQHSRCHPDAFCYNTPGSFTCQCKPGYQGDGFR | b9-H2O; b17; b27-H2O; b30; b34-NH3-NH3; y22-NH3; y27; y35-NH3; y35-H2O-NH3 |
|  |  |  |  |  | *β 195KDE* |  |

| ***m/z*** | **[M+H]^+^** | **charge**  **state** | **Δ [M+H]^+^** | **cross-linked lysines** | **peptide sequences** | **identified fragment ions** |
| --- | --- | --- | --- | --- | --- | --- |
| 897.4719 | 5379.7987 | 6 | -0.7 | K-1072 x K-961 | α 1051GIVTDPVRGNLYWTDWNRDNPKIE | b8-H2O; b18-NH3; y13; y13-H2O; y14-H2O; y16; y19; y23 |
|  |  |  |  |  | *β 952AKAFLHIPAKVIIGLAFDCVDK* | *b15-H2O; b16; b17-H2O; y18-H2O; b20; y21-NH3* |
| 919.6790 | 4594.3690 | 5 | -0.7 | K-1144 x K-948 | α 1128KVLEGLQYPFAVTSYGKNLYYTDWK | b10-H2O; b10-NH3; b18; b23; y7; y14-NH3; y14-H2O; y19; y22; y22-H2O; y22-NH3; y23; |
|  |  |  |  |  | *β 937IERLPLERNTmK* | *y1; y1-NH3-NH3; y2; y3; y4-NH3; y5; y8; y9; y10; precursor-H2O-H2O-H2O* |
| 769.4048 | 5379.7987 | 7 | -1.6 | K-1072 x K-961 | α 1051GIVTDPVRGNLYWTDWNRDNPKIE | b8-H2O; b20; b22; y3-NH3; y7; y8; y9-NH3; y12-H2O; y12-H2O-H2O; y13-H2O; y14-H2O; y16; y18; y20; y21; y22-H2O |
|  |  |  |  |  | *β 952AKAFLHIPAKVIIGLAFDCVDK* | *b9; b10; b15; b15-H2O; b15-NH3; b17-H2O; b18; b18-NH3; b19; b21-NH3; y18; y18-H2O; y21-H2O; y21-NH3* |
| 602.9208 | 3010.5673 | 5 | 2.5 | K-420 x K-949 | α 407QCVAEGSPQRVNGK | b7-NH3; b11-NH3-NH3; y3; y3-H2O; y4; y5; y8; y10-NH3; y12; y12-H2O; y13 |
|  |  |  |  |  | *β 939RLPLERNTMKK* | *b5-H2O; y2; y4; y5-H2O; y8; y8-H2O-H2O* |
| 792.9919 | 3960.9252 | 5 | 1.3 | K-1165 x K-194 | α 1145NLYYTDWKTNSVIAMDLAISKE | b6; b12; b15; b16-H2O; b16-NH3; b18-H2O; b21; b21-H2O; y4; y6-H2O; y16-H2O; y20; y20-H2O |
|  |  |  |  |  | *β 184DGLQFFTTFSK* | *y5; y6-H2O; y7; y9* |
| 660.9940 | 3960.9284 | 6 | -0.2 | K-627 x K-949 | α 601CAHDDARPALPSTQQLSVDSVFVLYNKEE | b4-NH3; b6-H2O; b10-NH3; b11-H2O; b14-NH3; b19-H2O-H2O; b21; b25-NH3; b26; y7-NH3; y10; y10-H2O; y13-H2O; y15; y15-H2O; y18-NH3; y23-NH3; y28-H2O; y28-NH3 |
|  |  |  |  |  | *β 949KTEAK* | *b2-NH3; b3-H2O* |

| ***m/z*** | **[M+H]^+^** | **charge**  **state** | **Δ [M+H]^+^** | **cross-linked lysines** | **peptide sequences** | **identified fragment ions** |
| --- | --- | --- | --- | --- | --- | --- |
| 1017.2195 | 5082.0786 | 5 | -2.0 | K-812 x K-420 | α 789CQHSRCHPDAFCYNTPGSFTCQCKPGYQGDGFRCMPGE | b11-H2O-NH3; b18; b29-H2O; b29-H2O-H2O; b33; b36-H2O; y12-H2O; y20-NH3; y23-H2O; y26-NH3; y34-H2O |
|  |  |  |  |  | *β 417VNGK* | *y2-NH3; y3* |
| 758.8790 | 3032.4960 | 4 | -0.6 | K-236 x K-194 | α 217SNGAYNIFANDRESIENLAK | b7; b16; b19-H2O; y6; y6-NH3; y9-H2O; y11; y17; y17-H2O; y19-H2O-H2O-NH3 |
|  |  |  |  |  | *β 190TTFSKK* | *y2; y5; b5-NH3;* |
| 835.1951 | 3337.7501 | 4 | 2.6 | K-1165 x K-1128 | α 1149TDWKTNSVIAMDLAISK | b5-H2O-H2O; b8; b9; b9-H2O-H2O; b10; b11; b14-NH3; b15-NH3; y3; y5-NH3; y8; y8-NH3; y9; y9-H2O |
|  |  |  |  |  | *β 1127RKVLEGLQYPF* | *b2; b3-NH3-NH3; b9-NH3; y9* |
| 954.2695 | 4767.3247 | 5 | -1.3 | K-1165 x K-236 | α 1145NLYYTDWKTNSVIAMDLAISK | b18-H2O-NH3; b20; b20-H2O; y1-NH3; y6-NH3; y2; y8; y8-NH3; y11; y11-H2O; y12-NH3; y13; y14; y16; y17-NH3-NH3; y20-NH3 |
|  |  |  |  |  | *β 217SNGAYNIFANDRESIENLAK* | *b9; b18; y2; y6; y11; y13; y15; y17-H2O; y19* |
| 618.7088 | 3089.5096 | 5 | 1.7 | K-160 x K-949 | α 143ESVAPYGGPSSSPAEEGK | b12-NH3-NH3; y2-H2O; y4; y5; y6; y7-NH3; y11; y16 |
|  |  |  |  |  | *β 940LPLERNTmKK* | *b6; y1; y3; y5; y8-NH3* |
| 661.1152 | 3301.5511 | 5 | -1.2 | K-829 x K-194 | α 813PGYQGDGFRCMPGEVSKTRCQLER | b8; b17; b22; b22-H2O-H2O; y3-NH3; y8; y10; y11; y12; y16; y18; y21; y21-H2O |
|  |  |  |  |  | *β 193SKK* | *b2* |
| 1214.2001 | 6066.9852 | 5 | -2.2 | K-257 x K-1159 | α 222NIFANDRESIENLAKSSNAGHQGVWVFEIGSPATAK | b20; b28-NH3; b31; b32; y4; y16-H2O-H2O; y18-H2O-H2O; y22; y22-NH3; y30-H2O-NH3; y31-H2O |
|  |  |  |  |  | *β 1152KTNSVIAMDLAISKEMDTF* | *b2; b5-H2O; b7; b12-NH3* |

| **laminin γ1 LEb2-4/nidogen 1 cross-links** | | | | | | |
| --- | --- | --- | --- | --- | --- | --- |
| ***m/z*** | **[M+H]^+^** | **charge**  **state** | **Δ [M+H]^+^** | **cross-linked lysines** | **peptide sequences** | **identified fragment ions** |
| 790.1746 | 3946.8377 | 5 | 1.6 | K-850 x K-953 | Lam: α 844LTGECLKCIYNTAGFYCDRCK | b13-NH3; b17-H2O; b19-H2O; y3-H2O; y12-H2O; y13-H2O |
|  |  |  |  |  | *Nid: β 944RNTmKKTEAK* | *y2-H2O; y2-NH3; y3* |
| 576.56867 | 4029.92557 | 7 | 2.8 | K-787 x K-513 | Lam: α (GSAS)771PCPCPGGSSCAIVPKTKE | b21-H2O; y5-H2O; y16-H2O |
|  |  |  |  |  | *Nid: β 499FTRQAEVTFLGHPGK* | *b6; b7; b7-NH3; b14-NH3; y5-HH3; y9-H2O; b11; y5; y8; y10-NH3; y13-H2O; y13-H2O; y14-H2O* |
| 798.1101 | 3189.4115 | 4 | 2.2 | K-829 x K-864 | Nid: α 821RCmPGEVSKTRCQLER | b7-NH3; b8-H2O-H2O; b9; b9-H2O; b12; b13; y9; y12; y13-H2O-H2O; y14; y15 |
|  |  |  |  |  | *Lam: β 860CDRCKEGF* | *b5; b7-NH3; y5; y5-H2O; y5-H2O-H2O-H2O; y6-H2O-H2O-H2O; y7-H2O; precursor-NH3-NH3-NH3* |
| 670.5260 | 3348.6061 | 5 | -1.6 | K-1152 x K-864 | Nid: α 1152KTNSVIAMDLAISKEMDTFHPHK | b4-H2O-H2O-H2O; b8-H2O; b11; b14-NH3; b15; b17-H2O; b20-H2O; b21-H2O; b22; b22-NH3; y5-H2O; y9; y12-NH3; y14-H2O-H2Oy17; y20; y20-NH3; y22 |
|  |  |  |  |  | *Lam: β 863CKEGF* | *b3; b3-H2O; b4-H2O-H2O; y4; precursor-NH3; precursor-NH3-NH3; precursor-NH3-NH3-NH3* |
| 798.1093 | 3189.4115 | 4 | 1.2 | K-829 x K-864 | Nid: α 821RCmPGEVSKTRCQLER | b7-NH3; b13; y12; y13-H2O-H2O; y14-NH3; y15 |
|  |  |  |  |  | *Lam: β 860CDRCKEGF* | *y5; y6; y7-H2O-H2O; precursor-H2O-H2O-H2O* |
| 649.7111 | 3244.5328 | 5 | -1.9 | K-160 x K-881 | Nid: α 149GGPSSSPAEEGKRNTF | b5-NH3; b9; b10-H2O; b12-H2O; b13; b14; b14-NH3; y9-NH3; y15; y15-NH3-NH3 |
|  |  |  |  |  | *Lam: β 868FGNPLAPNPADKCK* | *b5-NH3; y1-H2O; y2-H2O-H2O; y6-NH3; y6-H2O-H2Oy10; y11-NH3* |
| 815.3914 | 3258.5347 | 4 | 2.8 | K-879 x K-216 | Lam: α 863CKEGFFGNPLAPNPADKCK | b4; b9; b13-NH3; b14; b17; b18; y4; y6; y8-H2O; y8-NH3; y10; y10-H2O; y11; y15-H2O |
|  |  |  |  |  | *Nid: β 216KSNGAYNIF* | *b2; b6; b7-H2O* |
| ***m/z*** | **[M+H]^+^** | **charge**  **state** | **Δ [M+H]^+^** | **cross-linked lysines** | **peptide sequences** | **identified fragment ions** |
| 862.6376 | 3447.5276 | 4 | 0.3 | K-1230 x K-864 | Nid: α 1217CPDNTLGVDCIERK (AAAHHHHHH) | b12-H2O; b14-NH3-NH3; b15; b15-H2O-NH3-NH3; b16-H2O-NH3; b19-NH3; b20-NH3; b21; b22-H2O; y6-H2O-H2O; y15-NH3; y17-NH3; y19-NH3 |
|  |  |  |  |  | *Lam: β 863CKEGF* | *b3* |
| 920.1593 | 3677.6168 | 4 | -0.4 | K-850 x K-1072 | Lam: α844LTGECLKCIYNTAGFYCDRCKEGFF | b11-NH3; b13-NH3-NH3; b15; b16-NH3; b19-H2O-H2O; b20; b20-H2O-NH3; b21-H2O; y7; y7-H2O-H2O; y9-H2O-H2O; y11; y19; y21; y21-H2O; y22 |
|  |  |  |  |  | *Nid: β 1069DNPK* | *b3; y1-H2O; y2; y2-H2O; y3* |
| 864.9778 | 4320.8664 | 5 | -1.5 | K-829 x K-864 | Nid: α 802NTPGSFTCQCKPGYQGDGFRCmPGEVSKTR | b10-NH3; b12; b17; b20; b24-H2O-H2O; b27-NH3; y3; y3-NH3-NH3; y6-NH3; y8-H2O; y11; y11-NH3; y12; y14; y19; y20; y28-H2O |
|  |  |  |  |  | *Lam: β 863CKEGFF* | *b3; y5-NH3* |
| **laminin γ1 LEb2-4 N836D/nidogen-1 cross-links** | | | | | | |
| 806.7917 | 4029.9256 | 5 | 1.0 | K-787 x K-513 | Lam: α (GSAS)771PCPCPGGSSCAIVPKTKE | b10; b16; b18; y4; y9-H2O; y10-H2O; y12-H2O; y19-NH3; y21 |
|  |  |  |  |  | *Nid: β 499FTRQAEVTFLGHPGK* | *b5; b5-H2O; b8-H2O; b8-NH3; y4-H2O; y5; y10; y10-H2O-H2O; y13-H2O* |
| 790.1743 | 3946.8377 | 5 | 1.2 | K-850 x K-953 | Lam: α 844LTGECLKCIYNTAGFYCDRCK | b8-H2O-H2O; b13-NH3; b16-NH3; b17-NH3; b19-H2O; y5; y11-NH3-NH3; y12-H2O; y19 |
|  |  |  |  |  | *Nid: β 944RNTmKKTEAK* | *y2-H2O; y2-NH3; y2-H2O-H2O; y3; y5; precursor-NH3-NH3-NH3-NH3* |
| **laminin γ1 short arm/nidogen-1 cross-links** | | | | | | |
| 734.9332 | 3670.6359 | 5 | 0.3 | K-286 x K-829 | Lam:α271SYYYAISDFAVGGRCKCNGHASECVKNE | b13; b15-H2O; b17; b21-H2O; b22; b25; y11-H2O; y20; y22-NH3; y22-H2O-H2O; y23; y25; y26-H2O |
|  |  |  |  |  | *Nid:β827VSK* | *precursor-H2O-H2O* |
| 793.1883 | 3961.907 | 5 | 1.3 | K-961 x K-980 | Nid: α 954AFLHIPAKVIIGLAFDCVDK | b9; b16; b17; b19; b19-H2O; y12; y16-H2O; y18-NH3 |
|  |  |  |  |  | *Lam: β 967CETNHFGFGPEGCK* | *b7; y3; y7; y8; y8-NH3; y13-NH3* |
| ***m/z*** | **[M+H]^+^** | **charge**  **state** | **Δ [M+H]^+^** | **cross-linked lysines** | **peptide sequences** | **identified fragment ions** |
| 746.1791 | 4472.0333 | 6 | 1.1 | K-187 x K-1165 | Lam:α167EDGPWIPYQYYSGSCENTYSKANR | b7-H2O; b22-NH3; y6; y7; y16-NH3; y20; y20-H2O-H2O; y21 |
|  |  |  |  |  | *Nid:β1153TNSVIAMDLAISKE* | *y5-H2O; y6; y11; y12; y12-NH3; y13-NH3* |
| 555.0782 | 3325.4332 | 6 | -0.1 | K-160 x K-377 | Nid: α 144SVAPYGGPSSSPAEEGK | b7; b8-NH3; b13; b13-NH3; y7; y8; y8-H2O; y14; y14-H2O |
|  |  |  |  |  | *Lam: β 371DNTDGAKCERCRE* | *b12-H2O; y9; y10-H2O; y11-H2O* |
| 661.1581 | 3961.9074 | 6 | 1.2 | K-973 x K-980 | Nid: α 954AFLHIPAKVIIGLAFDCVDK | b10; y8; y8-NH3; y9-NH3; y17 |
|  |  |  |  |  | *Lam: β 967CETNHFGFGPEGCK* | *b7; b13; y1; y2-H2O; y6-H2O; y7; y7-NH3; y8; y8-NH3; y10-NH3; y12-NH3; y13; y13-H2O; y13-H2O-NH3; precursor-H2O-H2O-H2O* |
| **laminin γ1 short arm N836D/nidogen-1 cross-links** | | | | | | |
| 793.1893 | 3961.9074 | 5 | 2.5 | K-961 x K-980 | Nid: α 954AFLHIPAKVIIGLAFDCVDK | b14-NH3; b15; b16; b17; b19; b19-NH3; y8-NH3; y12; y18-NH3 |
|  |  |  |  |  | *Lam: β 967CETNHFGFGPEGCK* | *b7; y3-NH3; y7; y8; y8-NH3; y9; y13-H2O* |
| 806.7939 | 4029.9302 | 5 | 2.5 | K-645 x K-829 | Lam: α 622YIFRLHEATDYPWRPALSPFEFQK | b6-H2O-H2O; b7-H2O; b12; b14; b14-H2O; b17; b23; b23-NH3; y8; y11-H2O; y15; y20-H2O |
|  |  |  |  |  | *Nid: β 822CmPGEVSK* | *b7-NH3; y4-H2O* |
| 766.7306 | 4595.3524 | 6 | -1.1 | K-621 x K-829 | Lam: α 593DLVLEGAGLRVSVPLIAQGNSYPSETTVKYIFR | b8; b16; b16-H2O-H2O; b18-NH3; b29; b31-H2O; b31-H2O-H2O; b32-H2O; y7-H2O-H2O; y8; y10; y13-NH3; y14; y27; y27-NH3; y30; y31-NH3 |
|  |  |  |  |  | *Nid: β 822CMPGEVSK* | *b5-NH3; y1-H2O; y2-H2O* |
| 1076.7649 | 5379.8025 | 5 | -1.3 | K-513 x K-163 | Nid: α 505VTFLGHPGKLVLKQQFSGIDEHGHLTISTE | b14; b17-H2O; b20-H2O; b20-NH3; b22; y10 -H2O -NH3; y24; y26 |
|  |  |  |  |  | *Lam: β 151FHTSRPESFAIYKRTR* | *b8-NH3-NH3; b10; b11; b14; y14; b12-H2O* |

| ***m/z*** | **[M+H]^+^** | **charge**  **state** | **Δ [M+H]^+^** | **cross-linked lysines** | **peptide sequences** | **identified fragment ions** |
| --- | --- | --- | --- | --- | --- | --- |
| 831.6915 | 3323.7463 | 4 | -0.6 | K-563 x K-829 | Lam: α 557YFIAPVKFLGNQVLSYGQNLSFSFR | b10-H2O; b12-H2O; b13-H2O; b14-H2O-H2O; b18; b19; b19-H2O; b19-NH3-NH3; b20-H2O-H2O; b23; b24-NH3; y11; y11-H2O-H2O; y13; y14-H2O; y16; y21; y21-NH3-NH3; y24 |
|  |  |  |  |  | *Nid: β 827VSK* | *y1; y2-H2O; y2-NH3-NH3* |
| 909.6478 | 3635.5802 | 4 | -2.9 | K-377 x K-948 | Lam: α 359STGHGGHCTNCRDNTDGAKCER | b10; b10-NH3; b11-NH3; b13; b16; b19; y6; y6-NH3; y12-H2O; y15; y15-NH3; y16-NH3-NH3; y17; y18-NH3; y19; y20-NH3 |
|  |  |  |  |  | *Nid: β 945NTMKKTEAK* | *b4; y7; y7-NH3; y8; precursor-H2O-NH3; precursor-NH3-NH3-NH3* |
| 792.9923 | 3960.9333 | 5 | -0.2 | K-208 x K301 | Nid: α 195KDESQVPAVVGFSKGLVGFLWK | b6-H2O; b9-NH3; b13-NH3; b14-NH3; b18-NH3; b21; b21-NH3; y5-H2O; y8-H2O; y11; y13; y14-H2O; y16; y16-H2O; y17; y20; y20-H2O |
|  |  |  |  |  | *Lam: β 297NEFDKLmCNCK* | *b7; b10; b10-NH3; y8-H2O; y8-H2O-NH3; y9; y9-H2O; y10* |
| 661.1578 | 3961.9074 | 6 | 0.7 | K-961 x K-980 | Nid: α 954AFLHIPAKVIIGLAFDCVDK | b12; b12-NH3; b16; b17; b19; b19-NH3; y5-NH3; y8; y12; y13; y14-NH3; y17; y18-NH3; y19 |
|  |  |  |  |  | *Lam: β 967CETNHFGFGPEGCK* | *b6; b7; y1; y4-H2O; y7; y7-H2O; y8; y8-NH3; y10; y12-H2O-H2O; y13-NH3; precursor-NH3; precursor-H2O-H2O-H2O* |
| 793.1889 | 3961.9074 | 5 | 2.0 | K-961 x K-980 | Nid: α 954AFLHIPAKVIIGLAFDCVDK | b14-H2O; b15; b16; b17; b19; b19-NH3; y12; y15-H2O-H2O; y16-H2O |
|  |  |  |  |  | *Lam: β 967CETNHFGFGPEGCK* | *b7; y1; y3-NH3-NH3; y4-H2O; y5; y6; y7; y8; y9; y13-NH3* |

| ***m/z*** | **[M+H]^+^** | **charge**  **state** | **Δ [M+H]^+^** | **cross-linked lysines** | **peptide sequences** | **identified fragment ions** |
| --- | --- | --- | --- | --- | --- | --- |
| 829.6008 | 4143.9757 | 5 | -0.2 | K-377 x K-948 | Lam: α 371DNTDGAKCERCRENFFR | b7-H2O-NH3; b8; b11; b12; b13; b14-H2O; y11; y12; y14 |
|  |  |  |  |  | *Nid: β 937IERLPLERNTmKKTE* | *b5-H2O; b8-H2O; b13-H2O; b13-H2O-NH3; b14-H2O; b14-H2O-H2O; y6* |
| **intra-molecular laminin γ1 short arm cross-Links** | | | | | | |
| 788.8434 | 4728.0112 | 6 | 2.7 | K-980 x K-787 | α 966RCETNHFGFGPEGCKPCDCHHE | b6; b16-H2O; b18; b20; b20-NH3; y8; y8-NH3; y11-H2O; y13; y16; y17-NH3; y18; y18-NH3; y19-NH3; y20 |
|  |  |  |  |  | *β 786TKEVVCTHCPTGTAGKR* | *b6; b10; b11; b13-NH3; b15; y9-NH3; y11; y16-NH3* |
