# Supplementary material for: Analysis of Nidogen-1/Laminin γ1 Interaction by Cross-Linking, Mass Spectrometry, and Computational Modeling Reveals Multiple Binding Modes
Source: PLoS One. 2014 Nov 11;9(11):e112886. doi: 10.1371/journal.pone.0112886 (PMC4227867; doi:10.1371/journal.pone.0112886)
Supplement: Table S7 — Verified products of UV A-induced cross-linking. Peptide sequences written in parentheses are part of the protein affinity tags and do thus not belong to the native amino acid sequences of the proteins studied. For ambiguous cross-links, all potential cross-linked amino acids are listed. Within the peptide sequences, photo-leucine and photo-methionine are assigned with ‘z’ and ‘o’, respectively. Oxidized methionines are denoted with ‘m’. Loss of water or ammonium is indicated by addition of ‘−H2O’ or ‘−NH3’ to the fragment ion. (DOC) [file pone.0112886.s016.doc]

Table S 7. Verified products of UV A-induced cross-linking. Peptide sequences written in parentheses are part of the protein affinity tags and do thus not belong to the native amino acid sequences. For ambiguous cross-links, all possibly cross-linked amino acids are listed. Within the peptide sequences , photo-leucine and photo-methionine are assigned with ‘z’ and ‘o’, respectively. Oxidized methionines are denoted with ‘m’. Loss of water or ammonia is indicated by addition of ‘-H_2_O’ or ‘-NH_3_’ to the fragment ion.

| ***m/z***  **exp.** | **[M+H]^+^**  **calc.** | **charge**  **state** | **Δ [M+H]^+^**  **(ppm)** | **cross-linked**  **lysines** | **peptide sequences** | **identified fragment ions** |
| --- | --- | --- | --- | --- | --- | --- |
| **intra-molecular nidogen-1 cross-links** | | | | | | |
| 1002.2482 | 4005.9689 | 4 | 0.5 | z-990 x E-357 | α 988ASzHGGEPTTIIRQDLGSPE | b3-NH3-NH3 ; b12 ; b13-NH3 ; b15-H2O ; b17-H2O ; b18 ; b19-H2O-H2O-NH3 ; y8-NH3 ; y11 ; y19 ; y19-H2O |
|  |  |  |  |  | *β 342RFPQHHPQVIDVDEVE* | *b12 ; b14 ; b15 ; y1 ; y3 ; y3-H2O ; y4-NH3 ; y4-H2O-H2O-NH3 ; y5 ; y6 ; y6-H2O ; y11 ; y12-H2O ; precursor-H2O-NH3 ; precursor-NH3-NH3-NH3* |
| 506.2369 | 1516.6921 | 3 | 2.6 | R-1038 x z-990 | α 1033MDGTQRR | b5-H2O-H2O ; b6 ; b6-H2O ; y1 ; y2 ; y4 ; y4-H2O ; y5-H2O ; y6 |
|  |  |  |  |  | *β 988ASzHGGE* | *b3-NH3 ; b3-H2O-H2O ; b4 ; b5 ; y5 ; y6 ; y6-NH3 ; y6-H2O-NH3 ; precursor-NH3 ; precursor-H2O-NH3 ; precursor-H2O-H2O-NH3* |
| 1002.2488 | 4005.9689 | 4 | 1.1 | z-990 x E-357 | α 988ASzHGGEPTTIIRQDLGSPE | b3-NH3-NH3 ; b5 ; b7-NH3-NH3 ; b10 ; b17-H2O ; b19-NH3 ; b19-H2O-H2O-H2O ; y11 ; y11-H2O ; y11-NH3 ; y15 ; y16 ; y19 |
|  |  |  |  |  | *β 342RFPQHHPQVIDVDEVE* | *b7-H2O ; b12 ; y3 ; y3-H2O ; y4-NH3 ; y4-NH3-NH3 ; y4-H2O-H2O-NH3 ; y5 ; y6 ; y7 ; y9 ; y11 ; y12-H2O ; precursor-H2O-NH3 ; precursor-NH3-NH3-NH3* |
| 506.2363 | 1516.6921 | 3 | 1.5 | R-1038 x z-990 | α 1033MDGTQRR | b6 ; b6-H2O ; b6-NH3 ; b6-H2O-H2O ; y1 ; y2 ; y3 ; y4 ; y4-H2O ; y5-H2O |
|  |  |  |  |  | *β 988ASzHGGE* | *b3-NH3 ; b3-H2O-H2O ; b4 ; b4-NH3 ; b5 ; b6 ; y4 ; y5 ; y6 ; y6-NH3 ; y6-NH3-NH3 ; precursor-H2O ; precursor-NH3 ; precursor-H2O-NH3* |

| **inter-molecular laminin γ1 LEb2-4/nidogen-1 cross-links** | | | | | | |
| --- | --- | --- | --- | --- | --- | --- |
| ***m/z***  **exp.** | **[M+H]^+^**  **calc.** | **charge**  **state** | **Δ [M+H]^+^**  **(ppm)** | **cross-linked**  **lysines** | **peptide sequences** | **identified fragment ions** |
| 638.8135 | 1276.6202 | 2 | -0.3 | z-844 x K-1072 | Lam: α 844zTGECLK | b6 ; b6-H2O ; b6-NH3 ; b6-H2O-NH3-NH3 ; y5 ; y5-NH3 |
|  |  |  |  |  | *Nid: β 1069DNPK* | *b3 ; y1 ; y1-H2O ; y1-NH3 ; y2-NH3 ; y3-H2O ; precursor-H2O ; precursor-NH3 ; precursor-H2O-H2O ; precursor-H2O-NH3* |
| 638.8138 | 1276.6202 | 2 | 0.1 | z-844 x K-1072 | Lam: α 844zTGECLK | b6 ; b6-H2O ; b6-NH3 ; b6-H2O-NH3-NH3 ; y4-H2O ; y5 ; y5-NH3 |
|  |  |  |  |  | *Nid: β 1069DNPK* | *b3 ; y1 ; y1-H2O ; y1-NH3 ; y2-NH3 ; precursor-H2O ; precursor-NH3 ; precursor-NH3-NH3* |
| 638.8136 | 1276.6202 | 2 | -0.2 | z-844 x K-1072 | Lam: α 844zTGECLK | b1-H2O ; b6 ; b6-NH3 ; b6-H2O-NH3-NH3 ; y5 ; y5-NH3 |
|  |  |  |  |  | *Nid: β 1069DNPK* | *b3 ; y1 ; y1-H2O ; y1-NH3 ; precursor-NH3 ; precursor-NH3-NH3* |
| **intra-molecular laminin γ1 short arm cross-links** | | | | | | |
| 606.8145 | 2424.2378 | 4 | -0.7 | z-594 x W-327 | α 589LSAEDzVzEGAGLR | b5 ; b9 ; b9-H2O ; b12 ; b13 ; y4-NH3 ; y6-H2O ; y7-H2O-NH3 ; y12 ; y12-NH3 ; y13-NH3-NH3 |
|  |  |  |  |  | *β 326PWRRATAE* | *b2 ; b3-NH3-NH3 ; b7-H2O ; precursor-NH3-NH3 ; precursor-NH3-NH3-NH3* |
| 1128.8072 | 3384.3984 | 3 | 2.5 | z-713 x R-380 | α 711TPSzGPYSPCVLCTCNGHSE | b9-H2O ; b10 ; b13 ; b17 ; b17-H2O ; b18-NH3 ; b18-H2O-H2O-H2O ; y9 ; y11 ; y12-H2O ; y13 ; y13-NH3 ; y15-H2O ; y17-NH3 ; y17-NH3-NH3 ; y18-H2O ; y19-NH3 |
|  |  |  |  |  | *β 371DNTDGAKCER* | *b6-H2O ; b6-H2O-H2O ; b9-NH3-NH3 ; y1 ; y1-H2O-H2O-H2O ; y2-H2O ; y2-H2O-H2O ; y4 ; y4-NH3 ; y4-NH3-NH3 ; y5 ; y6-H2O ; y8 ; y8-NH3 ; y8-NH3-NH3 ; y9 ; y9-NH3 ; precursor-NH3-NH3 ; precursor-NH3-NH3-NH3* |

| ***m/z***  **exp.** | **[M+H]^+^**  **calc.** | **charge**  **state** | **Δ [M+H]^+^**  **(ppm)** | **cross-linked**  **lysines** | **peptide sequences** | **identified fragment ions** |
| --- | --- | --- | --- | --- | --- | --- |
| 1222.8151 | 4888.2523 | 4 | -2.8 | G-988 x z-483 | α 988GSL *(VPRGSAWSHPQFEKGGGSGGGSGGGSWSHPQFE)* | b1-NH3 ; b2-NH3-NH3-NH3 ; b5-NH3-NH3 ; b9-H2O ; b10 ; b12-H2O ; b14 ; b14-NH3 ; b15 ; b19 ; b19-NH3 ; b24 ; b30 ; b30-H2O ; b31-NH3-NH3-NH3 ; y11-NH3 ; y14 ; y17 ; y21 ; y21-NH3 ; y27-NH3-NH3 ; y29-H2O-H2O ; y30-NH3 ; y31 ; y31-NH3 |
|  |  |  |  |  | *β 478PGFFNzESSNPK* | *b9 ; b11 ; b11-H2O ; y7-H2O ; y8 ; y8-NH3 ; y9-NH3 ; precursor-H2O-H2O* |
| 525.7496 | 1050.4925 | 2 | -0.5 | z-638 x C-285/476/863/880 | α 636PAzSPFE | b3 ; b4 ; b4-H2O ; b6 ; b6-H2O ; y3 ; y3-H2O |
|  |  |  |  |  | *β 285/476/863/880 CK* | *b1 ; b1-H2O ; precursor-H2O ; precursor-H2O-NH3* |
| 566.6101 | 1697.8176 | 3 | -1.2 | z-483 x *tag (C-term)* | α 475RCKPGFFNzE | b5-NH3 ; b9 ; b9-NH3 ; y7 ; y8-NH3 |
|  |  |  |  |  | *β (GASGR)* | *y2 ; precursor-H2O-NH3* |
| 1128.8071 | 3384.3984 | 3 | 2.5 | z-713 x R-380 | α 711TPSzGPYSPCVLCTCNGHSE | b17 ; b18-H2O-H2O-H2O ; y9 ; y11 ; y12-H2O ; y13 ; y13-NH3 ; y14-NH3 ; y15-H2O ; y17-NH3 ; y17-NH3-NH3 ; y19-H2O ; y19-NH3 |
|  |  |  |  |  | *β 371DNTDGAKCER* | *b6-H2O ; y1 ; y1-NH3 ; y1-H2O-H2O-NH3 ; y2-H2O ; y2-H2O-H2O ; y4 ; y4-NH3 ; y4-NH3-NH3 ; y8 ; y8-NH3 ; precursor-NH3-NH3-NH3* |
