# Supplementary material for: Analysis of Nidogen-1/Laminin γ1 Interaction by Cross-Linking, Mass Spectrometry, and Computational Modeling Reveals Multiple Binding Modes
Source: PLoS One. 2014 Nov 11;9(11):e112886. doi: 10.1371/journal.pone.0112886 (PMC4227867; doi:10.1371/journal.pone.0112886)
Supplement: Table S8 — Rosetta clustering results of the final nidogen-1 NIDO domain models. The clustering radius was set to 10 Å. Clusters represented by models originating from the same initial low-resolution centroid model were merged. Shown are clusters with more than three member structures. Models, for which structural homologues within the PDB have been identified, are listed in italics. The remaining models share a similar topology to models generated based on highly homologous sequences of NIDO domains derived from related organisms. (DOC) [file pone.0112886.s017.doc]

Table S 8. Rosetta clustering results of the final nidogen-1 NIDO domain models. The clustering radius was set to 10 Å. Clusters represented by models originating from the same initial low-resolution centroid model were merged. Shown are clusters with more than 3 member structures. Models, for which structural homologues within the PDB have been identified, are listed in italics. The remaining models share a similar topology to models generated based on highly homologous sequences of NIDO domains derived from related organisms.

| **Cluster** | **Centroid model identifier** | **Rosetta total score** | **Size** |
| --- | --- | --- | --- |
| **1** | S_0035_17 | -319.502 | 10 |
| **2** | S_0037_97 | -317.883 | 29 |
| **3** | S_0058_71 | -311.148 | 9 |
| **4** | S_0053_97 | -310.042 | 16 |
| **5** | S_0013_65 | -307.592 | 9 |
| ***6*** | *S_0096_32* | *-306.554* | *8* |
| ***7*** | *S_0018_74* | *-306.444* | *8* |
| **8** | S_0039_20 | -300.332 | 9 |
