# Supplementary material for: Analysis of Nidogen-1/Laminin γ1 Interaction by Cross-Linking, Mass Spectrometry, and Computational Modeling Reveals Multiple Binding Modes
Source: PLoS One. 2014 Nov 11;9(11):e112886. doi: 10.1371/journal.pone.0112886 (PMC4227867; doi:10.1371/journal.pone.0112886)
Supplement: File S1 — Command line execution commands and flags used for computational modeling with Rosetta. (DOC) [file pone.0112886.s018.doc]

#### File S1. Command Line Execution Commands and Flags

Generation of Peptide Fragment Libraries

For both comparative modeling and *de novo* folding, fragment libraries consisting of 3mers and 9mers were created. These fragment libraries represent the distribution of conformations the peptide segments are likely to adopt within a protein structure.

First, the following command was used to create a checkpoint file and a PSIPRED secondary structure prediction file.

rosetta-3.4/rosetta_tools/fragment_tools/make_fragments.pl -verbose –nofrags <target.fasta> >& target_fragments.log &

In addition, JUFO9D was used to predict secondary structure elements.

bcl_align/scripts/runblast6 <target.fasta>

bcl.exe Jufo <target.fasta>

rosetta-3.4/rosetta_tools/fragment_tools/ss_pred_converter.py ­‐j <target.jufo9d_ss> > <target.jufo9d_ss2>

Next, a weights file and a configuration file were prepared.

weights file:

SecondarySimilarity 350 0.5 - psipred

SecondarySimilarity 250 0.5 - jufo

RamaScore 150 1.0 - psipred

RamaScore 150 1.0 - jufo

ProfileScoreL1 200 1.0 -

configuration file:

1 psipred 0.6

2 jufo9d 0.4

The Fragment Picker was executed with the below mentioned options summarized in a flag file. 1000 candidate fragments per sequence were generated and scored. The best-scoring 200 were kept and written to fragment files.

-in::file::vall rosetta-3.4/rosetta_tools/fragment_tools/vall.jul19.2011.gz
-in::file::checkpoint <target.checkpoint>
-in::file::fasta <target.fasta>
-frags::ss_pred <target.psipred_ss2> psipred <target.jufo9d_ss2> jufo
-out::file::frag_prefix <target_frags>
-frags::describe_fragments <target_frags.fsc>
-frags::scoring::config <weight_file.wghts>
-frags::frag_sizes 9 3
-frags::n_candidates 1000
-frags::n_frags 200
-frags::picking::quota_config_file <configuration_file.cfg>

Finally, the Fragment Picker was initiated.

rosetta-3.4/rosetta_source/bin/fragment_picker.default.linuxgccrelease -database rosetta-3.4/rosetta_database/ @<picker.options> >& picker.log &

Comparative Modeling

Target sequences were threaded onto template structures using the following command.

rosetta-3.4/rosetta_tools/protein_tools/scripts/thread_pdb_from_alignment.py --template=<template-ID> --target=<target-ID> --chain=<chain-ID> --align_format=clustal <alignment.aln> <template.pdb> <target_on_template.pdb>

The output PDB files were checked manually to identify gaps within the structure. Missing segments were specified in a Rosetta loops file, an example of which is shown here. All loops files are included in File S2. The designation of columns, from left to right, is: LOOP, number of starting loop anchor residue (integer), number of ending loop anchor residue (integer), cutpoint (integer, 0 = automatic cutpoint definition), skip rate probability (float), extend loop (boolean).

LOOP 8 12 0 0 0

LOOP 14 18 0 0 0

Options for the Rosetta Loop Modeling Application were summarized in a flags file. Loops were modeled using the Cyclic Coordinate Descent algorithm. For each template 1000 models were generated.

-loops:input_pdb <target_on_template.pdb>
-loops:loop_file <target_on_template.loops>
-loops:extended true
-loops:idealize_after_loop_close
-loops:relax fastrelax
-loops:fast
-out:[file:fullatom](../../../../%5C%5Cfullatom%5C) 
-out:prefix <target_on_template>
-ex1
-ex2
-nstruct 1000
-loops:frag_sizes 9 3
-loops:frag_files <target_frags.200.9mers> <target_frags.200.3mers>
-loops:remodel quick_ccd
-loops:refine refine_kic

The same approach was used to model the laminin γ1 L4 domain. However, idealizing the loops after closure led to unfolding of the models. Therefore, this option was disabled.

#-loops:idealize_after_loop_close

The Rosetta Loop Modeling application was executed with the following command.

rosetta-3.4/rosetta_source/bin/loopmodel.default.linuxgccrelease @<ccd.options> -database rosetta-3.4/rosetta_database/ >& ccd_target_on_template.log &

In cases, where Rosetta Loop Modeling failed to form the known disulfide bonds, they were built using the Relax application, including the flag "-in:fix_disulf".

rosetta-3.4/rosetta_source/bin/relax.default.linuxgccrelease -database rosetta-3.4/rosetta_database -in:[file:l](../../../../%5C%5Cl%5C) <best10pct_pdbs.list> -in:fix_disulf <domain.disulf> -out:[file:silent](../../../../%5C%5Csilent%5C) <relaxed_disulf_fixed.out> -out:nstruct 20 -out:[file:scorefile](../../../../%5C%5Cscorefile%5C) <relaxed_disulf_fixed.sc>

This flag requires a space-separated file with residue numbers of one disulfide pair per row, as shown in this example.

1 13
3 19
21 30

The 10% best-scoring models were used as input for the Rosetta Clustering application. Options were chosen as follows.

-in:[file:fullatom](../../../../%5C%5Cfullatom%5C)
-run:shuffle 
-cluster:radius -1
-cluster:input_score_filter 0

rosetta-3.4/rosetta_tools/protein_tools/clustering.py --silent=<relaxed_disulf_fixed.out> --rosetta=rosetta-3.4/rosetta_source/bin/cluster.default.linuxgccrelease --database=rosetta-3.4/rosetta_database/ --options=<cluster.options> <cluster_summary.txt> <cluster_histogram.txt>

Setting the clustering radius to -1 evokes automatic radius detection by Rostta. The clustering radii used for the different experiments are stated in the main text. For the laminin γ1 L4 domain, long loop regions (> 5 residues) were not considered for RMSD calculation during clustering. Hence, the following option was added to exclude certain residues.

-cluster:exclude_res <1 5-12 17-24 28-33 38-42 48 49 53-57 71-81 96-106 116-127 147-152 163-176>

Clustering was initialized with a python script.

rosetta-3.4/rosetta_tools/protein_tools/clustering.py --silent=<relaxed_disulf_fixed.out> --rosetta=rosetta-3.4/rosetta_source/bin/cluster.default.linuxgccrelease --database=rosetta-3.4/rosetta_database/ --options=<cluster.options> <cluster_summary.txt> <cluster_histogram.txt>

To generate score-vs-RMSD plots of the laminin γ1 L4 models, PDBs of the best-scoring models within the top 5 clusters were used as ‘native’ structures. All models were re-scored and compared to each of the extracted models. Again, long loop regions (> 5 residues) were disregarded for RMSD calculations.

rosetta-3.4/rosetta_source/bin/score_jd2.default.linuxgccrelease @<scoring.options>

-in:[file:silent](../../../../%5C%5Csilent%5C) <L4_rescored.out>
-in:[file:silent_struct_type](../../../../%5C%5Csilent_struct_type%5C) binary
-in:[file:fullatom](../../../../%5C%5Cfullatom%5C)
-database rosetta-3.4/rosetta_database/
-out:output
-out:[file:silent](../../../../%5C%5Csilent%5C) <L4_rmsd_vs_native_structure.out>
-out:[file:silent_struct_type](../../../../%5C%5Csilent_struct_type%5C) binary
-out:[file:fullatom](../../../../%5C%5Cfullatom%5C)
-evaluation:rmsd _< native_structure.pdb> _core_rmsd <L4core.txt>

To visually inspect models for energetic frustrations of single amino acids in Pymol, the total score of each residue was mapped to the b-factor column of the PDB-formatted model files.

scripts/score_to_b_factor.py --term=total <model.pdb> <model_sc2b.pdb>

De Novo Folding and Subsequent Model Validation

Models for the NIDO domain of nidogen-1 were obtained using Rosetta AbinitioRelax within the Rosetta Topology Broker framework. Before running Rosetta Topology Broker, a setup file had to be created created.

CLAIMER SequenceClaimer

FILE NIDO.fasta

END_CLAIMER

Backbone and side chain relaxation during full atom refinement may lead to the loss of secondary structure elements and distortion of an originally correctly identified protein fold. Thus, low-resolution centroid models of NIDO domain were generated initially.

-in
     -file
          -fasta <NIDO.fasta>
          -frag3 <NIDO_frags.200.3mers>
          -frag9 <NIDO_frags.200.9mers>
-abinitio
     -increase_cycles 10
     -rg_reweight 0.5
     -rsd_wt_helix 0.5
     -rsd_wt_loop 0.5
-kill_hairpins <NIDO.psipred_ss2>
-use_filters true
-psipred_ss2 <NIDO.psipred_ss2>
-broker
     -setup <broker_setup.tpb>
-run
     -protocol broker
     -reinitialize_mover_for_each_job
-score
     -find_neighbors_3dgrid
-out
     -output
     -nstruct 100
-file
     -silent <NIDO.out>
     -silent_struct_type binary
     -scorefile <NIDO.out>
-overwrite

The Topology Broker was initiated with the following command line.

rosetta-3.4/rosetta_source/bin/minirosetta.default.linuxgccrelease @<broker.options> -database rosetta-3.4/rosetta_database -out:[file:silent](../../../../%5C%5Csilent%5C) <NIDO_centroid-$NUM.out> -out:[file:scorefile](../../../../%5C%5Cscorefile%5C) <NIDO_centroid-$NUM.sc>

MAMMOTH was used to compare the structures of the NIDO models to a precompiled PISCES library of PDB structures (soluble proteins, sequence ID <25%, resolution <2.0 Å) or to models of highly similar NIDO domains from other organisms, respectively.

mammoth -e <all_generated_models.list> -p <structures_to_compare_with.list> <comparison.out>

To facilitate MAMMOTH analysis, PDB files must not contain unnatural amino acids and are restricted to 3000 ATOM lines. Therefore, prior to structure comparison, side chain atoms were removed and unnatural amino acids were converted into their naturally occurring counterparts.

bcl.exe PDBConvert <input.pdb> -bcl_pdb -convert_to_natural_aa_type -aaclass AABackBone -output_prefix <output>

Validated NIDO models were subsequently subjected to full-atom refinement. Side chains and backbone were relaxed sequentially to prevent distortion of secondary structure elements.

First, Rosetta Relax was performed on the side chains exclusively.

rosetta-3.4/rosetta_source/bin/relax.default.linuxgccrelease -database rosetta-3.4/rosetta_database -in:[file:l](../../../../%5C%5Cl%5C) <models_with_common_topologies.list> -relax:fast -relax:bb_move false -out:pdb -out:prefix <sidechain_relax_$NUM> -out:nstruct 50 -out:[file:scorefile](../../../../%5C%5Cscorefile%5C) <sidechain_relax_$NUM.sc>

Second, the backbone conformation of the best scoring output model was optimized.

rosetta-3.4/rosetta_source/bin/relax.default.linuxgccrelease -database rosetta-3.4/rosetta_database -in:[file:l](../../../../%5C%5Cl%5C) < best_relaxed_sidechain_models.list> -relax:fast -relax:chi_move false -out:pdb -out:prefix <mainchain_relax_$NUM> -out:nstruct 1 -out:[file:scorefile](../../../../%5C%5Cscorefile%5C) <mainchain_relax_$NUM.sc>

These steps were repeated once and, finally, the entire model was subjected to optimization in ‘thorough relax’ mode.

rosetta-3.4/rosetta_source/bin/relax.default.linuxgccrelease -database rosetta-3.4/rosetta_database -in:[file:l](../../../../%5C%5Cl%5C) < best_relaxed_sidechain_models.list> -relax:thorough -out:pdb -out:prefix <full_relax_$NUM> -out:nstruct 50 -out:[file:scorefile](../../../../%5C%5Cscorefile%5C) <full_relax_$NUM.sc>
