# Supplementary material for: Analysis of Nidogen-1/Laminin γ1 Interaction by Cross-Linking, Mass Spectrometry, and Computational Modeling Reveals Multiple Binding Modes
Source: PLoS One. 2014 Nov 11;9(11):e112886. doi: 10.1371/journal.pone.0112886 (PMC4227867; doi:10.1371/journal.pone.0112886)
Supplement: File S2 — Rosetta loops files generated for comparative modeling. The position of the loops was determined based on the sequence alignments of the target sequences (listed in italics) to the respective templates. The scheme for template sequences is termed ‘PDB-entry_domain-name_chain-identifier’. (DOC) [file pone.0112886.s019.doc]

#### File S2. Rosetta Loops Files for Comparative Modeling

Nidogen-1 EGF-like2

Template 1GL4_EGF1_A

LOOP 19 23 0 0 0

Template 1YO8_EGF1_A

LOOP 8 16 0 0 0

LOOP 27 31 0 0 0

Template 1YO8_EGF2_A

LOOP 20 24 0 0 0

Template 3S94_EGF1_A

LOOP 20 24 0 0 0

Template 3V64_EGF3_D

LOOP 13 17 0 0 0

LOOP 21 25 0 0 0

LOOP 33 38 0 0 0

Nidogen-1 EGF-like3

Template 1SZB_EGF_A

LOOP 6 10 0 0 0

LOOP 14 18 0 0 0

LOOP 35 39 0 0 0

Template 1TOZ_EGF11_A

LOOP 35 39 0 0 0

Template 1TOZ_EGF12_A

LOOP 9 13 0 0 0

LOOP 35 39 0 0 0

Template 1UZJ_EGF27_A

LOOP 13 17 0 0 0

LOOP 36 40 0 0 0

Template 2BO2_EGF2_A

LOOP 6 10 0 0 0

LOOP 36 40 0 0 0

Template 2W86_EGF13_A

LOOP 13 17 0 0 0

LOOP 35 39 0 0 0

Template 2W86_EGF14_A

LOOP 13 17 0 0 0

LOOP 36 40 0 0 0

Template 3S94_EGF2_A

LOOP 13 17 0 0 0

LOOP 35 39 0 0 0

Nidogen-1 EGF-like4

Template 1TOZ_EGF11_A

LOOP 15 19 0 0 0

LOOP 23 27 0 0 0

LOOP 38 42 0 0 0

Template 1YO8_EGF2_A

LOOP 13 18 0 0 0

LOOP 23 28 0 0 0

Nidogen-1 EGF-like5

Template 1SZB_EGF_A

LOOP 8 13 0 0 0

LOOP 34 38 0 0 0

Template 1TOZ_EGF11_A

LOOP 6 10 0 0 0

LOOP 32 37 0 0 0

Template 1TOZ_EGF12_A

LOOP 32 37 0 0 0

Template 1UZJ_EGF26_A

LOOP 8 12 0 0 0

LOOP 34 38 0 0 0

Template 1UZJ_EGF27_A

LOOP 9 13 0 0 0

LOOP 32 36 0 0 0

Template 1YO8_EGF1_A

LOOP 18 22 0 0 0

LOOP 24 29 0 0 0

Template 2BO2_EGF2_A

LOOP 6 10 0 0 0

LOOP 32 38 0 0 0

Template 2W86_EGF13_A

LOOP 11 15 0 0 0

LOOP 33 38 0 0 0

Template 2W86_EGF14_A

LOOP 8 12 0 0 0

LOOP 32 36 0 0 0

Template 3P5B_EGF2_L

LOOP 8 12 0 0 0

LOOP 32 36 0 0 0

Nidogen-1 EGF-like6

Template 1TOZ_EGF11_A

LOOP 13 17 0 0 0

Template 2W86_EGF14_A

LOOP 10 15 0 0 0

LOOP 28 34 0 0 0

Template 3P5B_EGF1_L

LOOP 32 36 0 0 0

Template 3S94_EGF1_A

LOOP 20 24 0 0 0

LOOP 29 34 0 0 0

Template 3S94_EGF2_A

LOOP 32 36 0 0 0

Template 3V64_EGF3_D

LOOP 32 36 0 0 0

Nidogen-1 TY1

Template 1icf_ty1

LOOP 16 27 0 0 0

LOOP 69 73 0 0 0

Template 2dsr_ty1

LOOP 13 17 0 0 0

LOOP 45 50 0 0 0

LOOP 55 59 0 0 0

Laminin γ1 LEa3

Template 1klo_LEb4_A

LOOP 14 18 0 0 0

Template 1npe_LEb4_A

LOOP 14 18 0 0 0

Template 4aqs_LEa3_A

LOOP 8 12 0 0 0

LOOP 14 18 0 0 0

Laminin γ1 LEa4

Template 1klo_LEb3_A

LOOP 7 11 0 0 0

LOOP 19 23 0 0 0

LOOP 39 43 0 0 0

Template 1klo_LEb4_A

LOOP 7 11 0 0 0

LOOP 43 47 0 0 0

Template 1npe_LEb3_B

LOOP 7 11 0 0 0

LOOP 19 23 0 0 0

LOOP 39 43 0 0 0

Template 1npe_LEb4_B

LOOP 7 11 0 0 0

LOOP 43 47 0 0 0

Template 4aqs_LEa3_A

LOOP 9 16 0 0 0

Template 4aqt_LEa2_A

LOOP 8 12 0 0 0

LOOP 14 18 0 0 0

Laminin γ1 LEa5.2

Template 1klo_LEb2_A

LOOP 2 6 0 0 0

LOOP 18 22 0 0 0

Template 1klo_LEb4_A

LOOP 27 32 0 0 0

Template 1npe_LEb2_B

LOOP 2 6 0 0 0

LOOP 18 22 0 0 0

Template 1npe_LEb4_B

LOOP 27 32 0 0 0

Laminin γ1 LEb1

Template 1klo_LEb2_A

LOOP 7 15 0 0 0

LOOP 37 42 0 0 0

Template 1klo_LEb3_A

LOOP 9 19 0 0 0

LOOP 41 45 0 0 0

Template 1npe_LEb2_B

LOOP 7 15 0 0 0

LOOP 37 42 0 0 0

Template 1npe_LEb3_B

LOOP 9 19 0 0 0

LOOP 41 45 0 0 0

Template 2y38_LEa1_A

LOOP 11 15 0 0 0

LOOP 32 39 0 0 0

Template 4aqs_LEa1_A

LOOP 12 16 0 0 0

LOOP 37 45 0 0 0

Template 4aqs_LEa2_A

LOOP 12 19 0 0 0

LOOP 37 42 0 0 0

Template 4aqt_LEa1_A

LOOP 12 16 0 0 0

LOOP 32 37 0 0 0

Template 4aqt_LEa2_A

LOOP 10 17 0 0 0

LOOP 37 42 0 0 0

Laminin γ1 LEb5

Template 4aqs_LEa2_A

LOOP 7 22 0 0 0

LOOP 42 47 0 0 0

Template 4aqs_LEa3_A

LOOP 7 19 0 0 0

LOOP 41 45 0 0 0

Template 4aqt_LEa2_A

LOOP 7 22 0 0 0

LOOP 41 45 0 0 0

Laminin γ1 L4

Template 1CX1_A

LOOP 4 13 0 0 0

LOOP 16 34 0 0 0

LOOP 37 43 0 0 0

LOOP 47 63 0 0 0

LOOP 70 82 0 0 0

LOOP 85 89 0 0 0

LOOP 95 107 0 0 0

LOOP 115 140 0 0 0

LOOP 146 153 0 0 0

LOOP 162 176 162 0 0

Template 1D7B_A

LOOP 4 13 0 0 0

LOOP 16 25 0 0 0

LOOP 27 34 0 0 0

LOOP 37 43 0 0 0

LOOP 47 63 0 0 0

LOOP 70 82 0 0 0

LOOP 85 89 0 0 0

LOOP 95 107 0 0 0

LOOP 115 140 0 0 0

LOOP 146 153 0 0 0

LOOP 162 176 162 0 0

Template 1DYO_A

LOOP 4 13 0 0 0

LOOP 16 25 0 0 0

LOOP 27 34 0 0 0

LOOP 37 43 0 0 0

LOOP 47 63 0 0 0

LOOP 70 82 0 0 0

LOOP 85 89 0 0 0

LOOP 95 107 0 0 0

LOOP 115 140 0 0 0

LOOP 146 153 0 0 0

LOOP 162 176 162 0 0

Template 1GU3_A

LOOP 1 13 13 0 0

LOOP 16 34 0 0 0

LOOP 37 43 0 0 0

LOOP 47 63 0 0 0

LOOP 70 82 0 0 0

LOOP 85 89 0 0 0

LOOP 95 107 0 0 0

LOOP 115 140 0 0 0

LOOP 146 153 0 0 0

LOOP 162 176 162 0 0

Template 1GUI_A

LOOP 1 13 13 0 0

LOOP 16 25 0 0 0

LOOP 27 34 0 0 0

LOOP 37 43 0 0 0

LOOP 47 63 0 0 0

LOOP 70 82 0 0 0

LOOP 85 89 0 0 0

LOOP 95 107 0 0 0

LOOP 115 140 0 0 0

LOOP 146 153 0 0 0

LOOP 162 176 162 0 0

Template 1K42_A

LOOP 1 13 13 0 0

LOOP 16 25 0 0 0

LOOP 27 34 0 0 0

LOOP 37 43 0 0 0

LOOP 47 63 0 0 0

LOOP 70 82 0 0 0

LOOP 85 89 0 0 0

LOOP 95 107 0 0 0

LOOP 115 140 0 0 0

LOOP 146 153 0 0 0

LOOP 162 176 162 0 0

Template 1WKY_A

LOOP 4 13 0 0 0

LOOP 16 25 0 0 0

LOOP 27 34 0 0 0

LOOP 37 43 0 0 0

LOOP 47 63 0 0 0

LOOP 70 82 0 0 0

LOOP 85 89 0 0 0

LOOP 95 107 0 0 0

LOOP 115 140 0 0 0

LOOP 146 153 0 0 0

LOOP 162 176 162 0 0

Template 1WMX_A

LOOP 4 13 0 0 0

LOOP 16 34 0 0 0

LOOP 37 43 0 0 0

LOOP 47 63 0 0 0

LOOP 70 82 0 0 0

LOOP 85 89 0 0 0

LOOP 95 107 0 0 0

LOOP 115 140 0 0 0

LOOP 146 153 0 0 0

LOOP 162 176 162 0 0

Template 2ZEW_A

LOOP 1 13 13 0 0

LOOP 16 25 0 0 0

LOOP 27 34 0 0 0

LOOP 37 43 0 0 0

LOOP 47 63 0 0 0

LOOP 70 82 0 0 0

LOOP 85 89 0 0 0

LOOP 95 107 0 0 0

LOOP 115 140 0 0 0

LOOP 146 153 0 0 0

LOOP 162 176 162 0 0

Template 2ZEW_B

LOOP 1 13 13 0 0

LOOP 16 25 0 0 0

LOOP 27 34 0 0 0

LOOP 37 43 0 0 0

LOOP 47 63 0 0 0

LOOP 70 82 0 0 0

LOOP 85 89 0 0 0

LOOP 95 107 0 0 0

LOOP 115 140 0 0 0

LOOP 146 153 0 0 0

LOOP 162 176 162 0 0

Template 2ZEZ_A

LOOP 1 25 25 0 0

LOOP 27 34 0 0 0

LOOP 37 43 0 0 0

LOOP 47 63 0 0 0

LOOP 70 82 0 0 0

LOOP 85 89 0 0 0

LOOP 95 107 0 0 0

LOOP 115 140 0 0 0

LOOP 146 153 0 0 0

LOOP 162 176 162 0 0

Template 3F95_A

LOOP 8 12 0 0 0

LOOP 17 32 0 0 0

LOOP 37 58 0 0 0

LOOP 71 79 0 0 0

LOOP 85 89 0 0 0

LOOP 98 106 0 0 0

LOOP 116 139 0 0 0

LOOP 150 154 0 0 0

LOOP 168 172 0 0 0

Template 3OEA_A

LOOP 1 13 13 0 0

LOOP 16 25 0 0 0

LOOP 27 34 0 0 0

LOOP 37 43 0 0 0

LOOP 47 63 0 0 0

LOOP 70 82 0 0 0

LOOP 85 89 0 0 0

LOOP 95 107 0 0 0

LOOP 115 140 0 0 0

LOOP 146 153 0 0 0

LOOP 162 176 162 0 0

Template 3ZXJ_A

LOOP 1 13 13 0 0

LOOP 16 25 0 0 0

LOOP 27 34 0 0 0

LOOP 37 43 0 0 0

LOOP 47 63 0 0 0

LOOP 70 82 0 0 0

LOOP 85 89 0 0 0

LOOP 95 107 0 0 0

LOOP 115 140 0 0 0

LOOP 146 153 0 0 0

LOOP 162 176 162 0 0
